# Primary and Secondary Coordination Sphere Effects in the Cobalt Complex-Catalyzed Electrocatalytic O_2_ Reduction to Water

**DOI:** 10.1021/jacs.5c11990

**Published:** 2025-10-21

**Authors:** Abhinav Bairagi, Max T. G. M. Derks, Aleksandr Y. Pereverzev, Jana Roithová

**Affiliations:** Institute for Molecules and Materials, 6029 Radboud University, Heyendaalseweg 135, 6525 AJ Nijmegen, The Netherlands

## Abstract

Molecular cobalt-based complexes generally favor O_2_ reduction to H_2_O_2_. However, a full reduction of O_2_ to H_2_O is desired for fuel cell applications, which drives the current research in oxygen reduction electrocatalysis. We report a new Co­(III) complex based on an N_4_O-coordinating ligand (UMAPA) that electrochemically reduces oxygen to water in the presence of acetic acid in acetonitrile. By combining electrochemistry-coupled electrospray ionization mass spectrometry (EC-ESI-MS), cyclic voltammetry, ultraviolet–visible (UV–vis) spectrophotometry, and rotating ring disk electrode voltammetry analyses, we unraveled the rarely reported integral role of both primary and secondary coordination in a single catalyst. The chronoamperometry EC-ESI-MS experiments allowed us to effectively monitor the catalyst’s activation steps and the formation of the cobalt­(III) hydroperoxo intermediate. Consequently, we deciphered how acetic acid and the secondary coordination sphere of the Co­(III) complex facilitated the subsequent water-forming steps. Furthermore, we characterized the key Co­(III)-hydroperoxo and Co­(III)-hydroxo intermediates by using cryogenic ion spectroscopy. Our experiments also showed that the cobalt complex undergoes ligand hydroxylation under oxygen-rich conditions. Interestingly, chronoamperometry EC-ESI-MS showed that the hydroxylated cobalt complex remains active for electrocatalytic oxygen reduction (ORR). We then explored the significance of different coordination configurations of the same ligand around the Co center in ORR. The Co complex with the protonated UMAPA ligand exhibited N_4_ coordination and slower preactivation steps than the Co complex with the deprotonated N_4_O-coordinating UMAPA. Surprisingly, the CV experiments revealed that these small changes in the ligand configuration around the Co center result in a drastic change in the ORR activity. EC-ESI-MS was instrumental in determining the molecular-level details of the electrocatalytic ORR catalyzed by the Co complex, which conventional electroanalytical techniques alone cannot provide.

## Graphical abstract

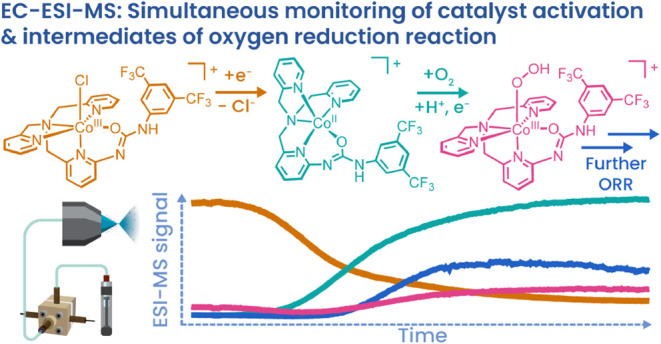

## Introduction

The electrochemical oxygen reduction reaction (ORR) is the key half-reaction for improving the efficiency of fuel cells and aerobic substrate oxidation reactions.^1–3^ Incomplete 2e^–^/2H^+^ oxygen reduction produces hydrogen peroxide, which is an indispensable industrial and laboratory oxidant. Conversely, complete 4e^–^/4H^+^ reduction of O_2_ to water is highly desirable for proper fuel cell function and enzyme-inspired substrate oxidation reactions.^4–6^ Transition-metal-based molecular complexes have emerged as highly tunable and characterizable catalysts for selective electrocatalytic O_2_ reduction.^5,7^ One distinct feature of these molecular catalysts is that their primary and secondary coordination spheres can be tuned to favor one product over another in the electrocatalytic ORR.^8^ However, developing such selective molecular catalysts requires a molecular-level understanding of the electrocatalytic ORR mechanism.

So far, most studies have employed a combination of offline EPR, UV–vis, and conventional electrochemical techniques to investigate the ORR mechanism.^7,9^ These offline approaches usually screen the bulk solution and, therefore, often fall short in providing a molecular-level picture of the reaction.^10^ Recently, the *in situ* surface-enhanced resonance Raman spectroscopy coupled with a rotating disk electrode (SERRS-RDE) method has emerged as a remarkable tool for studying the oxygen reduction reaction at the electrode interface.^11^ However, the *in situ* SERRS-RDE is mainly used with electrode-immobilized ORR catalysts to study steady-state O_2_ reduction. While this method provides molecular-level information, the information obtained can only be partially translated into homogeneous electrocatalytic ORR. Alternatively, Gu et al. showed that electrochemistry coupled with mass spectrometry (EC-MS) is an efficient tool for capturing the homogeneous ORR intermediates and deciphering the ORR mechanisms.^12^ Our group has also developed an integrated electrochemistry-electrospray ionization mass spectrometry technique (EC-ESI-MS) and successfully studied the mechanisms of electrocatalytic CO_2_ and O_2_ reduction reactions.^13–15^ Here, we have applied similar EC-ESI-MS techniques to explain the mechanism of electrocatalytic ORR to water by a new cobalt complex.

Cobalt complexes, especially the ones based on macrocycles, are a distinct class of oxygen reduction reaction (ORR) catalysts that selectively reduce O_2_ to H_2_O_2_.^16,17^ Although many mononuclear and binuclear cobalt complexes based on porphyrins and corroles are reported to reduce O_2_ to H_2_O selectively,^7,18^ the nonheme cobalt complexes capable of selectively reducing O_2_ to H_2_O are rare.^19^ Due to their potential applications, many recent works have aimed to direct the cobalt-catalyzed O_2_ reduction to H_2_O.

One tactic to achieve 4e^–^/4H^+^ O_2_ reduction to water involves adding redox mediators such as benzoquinone/hydroquinone, which provide the extra 2e^–^/2H^+^ to mediate cobalt-catalyzed O_2_ reduction to H_2_O.^20,21^ Additionally, two key approaches have been recently reported to develop nonheme cobalt complexes capable of O_2_ reduction to water via direct catalyst modifications (Scheme 1). The first one directly incorporates quinone or phenol units into the ligand framework, which works in tandem with the cobalt metal center to reduce O_2_ to H_2_O.^22–24^ The second approach takes inspiration from the enzymes and modifies the secondary coordination sphere of ligands to shuttle protons around the cobalt center.^25–27^

**Scheme 1:**
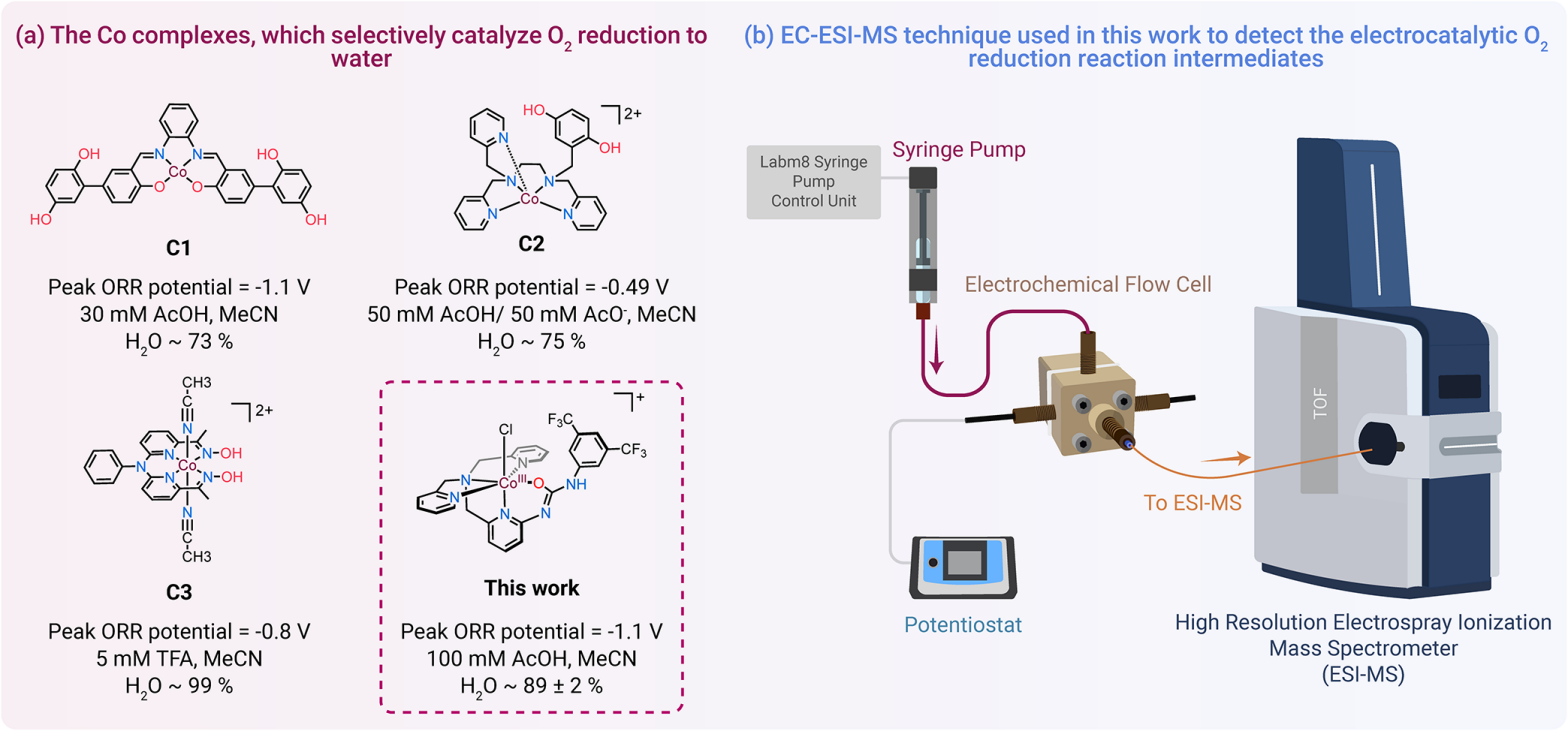
(a) Nonheme Co Complexes Capable of Selective Electrocatalytic O_2_ Reduction to Water^22,23,26^ and (b) Schematics of the Electrochemistry-Electrospray Ionization Hyphenated Mass Spectrometry Technique (EC-ESI-MS)

In this work, we employed the second approach and designed a nonheme Co­(III) complex [(UMAPA)­Co^III^(Cl)]Cl featuring a new N_4_O coordinating anionic UMAPA ligand (Scheme 1). The UMAPA ligand is a urea derivative of the extensively studied TPA (tris­(2-pyridylmethyl)­amine) ligand.^28,29^ Unlike the Co-macrocyclic complexes, [(UMAPA)­Co^III^(Cl)]­Cl was found to be highly selective for the reduction of O_2_ to water in an O_2_-saturated acetonitrile solution with acetic acid. As cyclic voltammetry alone cannot provide molecular-level insights, we used integrated chronoamperometry, voltammetry, and electrospray ionization mass spectrometry (EC-ESI-MS) to monitor key intermediates of electrocatalytic ORR catalyzed by [(UMAPA)­Co^III^(Cl)]­Cl, including species with hydroxylated ligands. By further combining the EC-ESI-MS, cyclic voltammetry, UV–vis spectroscopy, and rotating ring disk electrode voltammetry results, we uncovered how both primary and secondary coordination spheres influence catalyst activity. These findings show that even minor structural modifications can drastically alter the electrocatalytic ORR performance of a catalyst.

## Results

### Electrocatalytic Oxygen Reduction Reaction (ORR) Activity of the [(UMAPA)­Co^III^(Cl)]Cl Complex

The synthesis and characterization data of the UMAPA ligand and cobalt [(UMAPA)­Co^III^(Cl)]Cl complex are presented in the Supporting Information (SI) (for characterization data, see Figures S1–S5). We first probed the electrocatalytic ORR activity of the [(UMAPA)­Co^III^(Cl)]Cl complex using cyclic voltammetry (CV) in acetonitrile with 0.1 M TBAPF_6_ (TBA = tetrabutylammonium) as the supporting electrolyte. Unless stated otherwise, CVs were recorded at a scan rate of 100 mV s^–1^. Under argon, the CV of the [(UMAPA)­Co^III^(Cl)]Cl complex exhibited a reduction feature at *E*_pc_= −0.66 V vs Fc^+^/Fc (Figures 1a and S6–S7), which we attributed to the Co^III/II^ reduction process. On the reverse CV scan, a reoxidation peak appeared at *E*_pa_= −0.14 V vs Fc^+^/Fc, possibly corresponding to the Co^II/III^ oxidation. At higher scan rates (1000–1500 mV s^–1^), this oxidation peak nearly disappeared, while a new oxidation feature emerged at around −0.52 V vs Fc^+^/Fc (Figure S6). Although the Co^III/II^ process at *E*_1/2_ (Co^III/II^) = −0.42 V vs Fc^+^/Fc is diffusion-controlled and homogeneous, the large Δ*E*_p_= 520 mV indicates an irreversible redox nature (Figures 1a and S6). The irreversibility of the Co^III/II^ process possibly arises due to Cl^–^ loss during the Co­(III) to Co­(II) reduction, forming the [(UMAPA)­Co^II^]^+^ complex, which then oxidizes at a more positive potential. In fact, we could track this transformation during EC-ESI-MS experiments, as discussed below (Figure 2). At faster scan rates, the Cl^–^ loss was suppressed, resulting in a new oxidation feature near −0.52 V vs Fc^+^/Fc, possibly corresponding to the oxidation of the [(UMAPA)­Co^II^(Cl)] complex (Figure S6).

**Figure 1:**
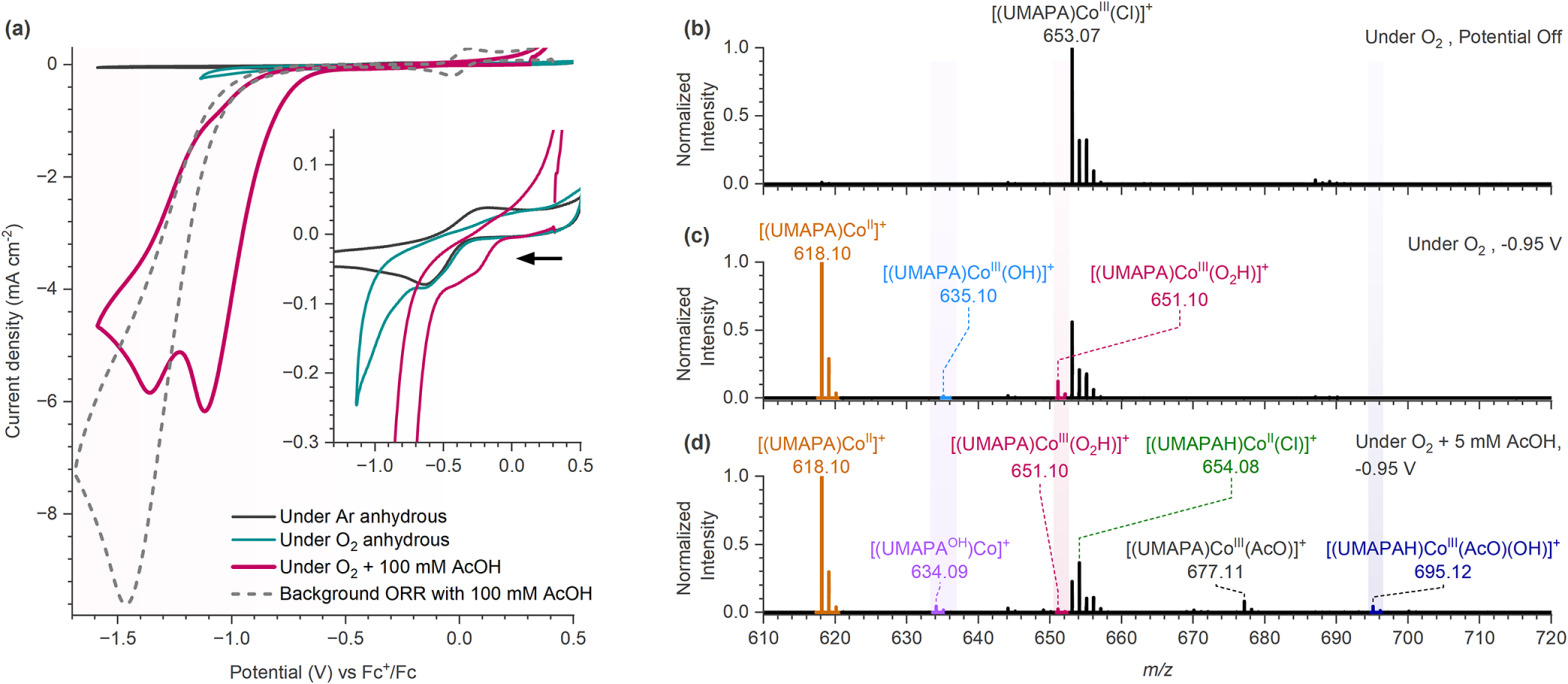
(a) Cyclic voltammetry profile of the [(UMAPA)­Co^III^(Cl)]­Cl complex (0.5 mM) under anhydrous argon (black), O_2_ saturation (green), and O_2_ saturation with 100 mM AcOH (200 equiv, pink). The dashed gray trace shows the background ORR without the cobalt complex. CVs were recorded at a 100 mV s^–1^ scan rate in 0.1 M TBAPF_6_ in anhydrous MeCN as the electrolyte. The inset shows the zoomed-in Co^III/II^ process. Potentials were scanned in the negative direction first. (b) EC-ESI-MS spectrum under O_2_ (anhydrous conditions) without any applied potential, (c) EC-ESI-MS spectrum under O_2_ (anhydrous conditions) at −0.95 V vs Fc^+^/Fc, and (d) EC-ESI-MS spectrum under O_2_ with 5 mM acetic acid (100 equiv) at −0.95 V vs Fc^+^/Fc. The spectra are normalized to the intensity of the base peak. All EC-ESI-MS spectra were acquired from a solution of the 0.05 mM (1 equiv) [(UMAPA)­Co^III^(Cl)]Cl complex in MeCN at a flow rate of 15 μL min^–1^.

**Figure 2:**
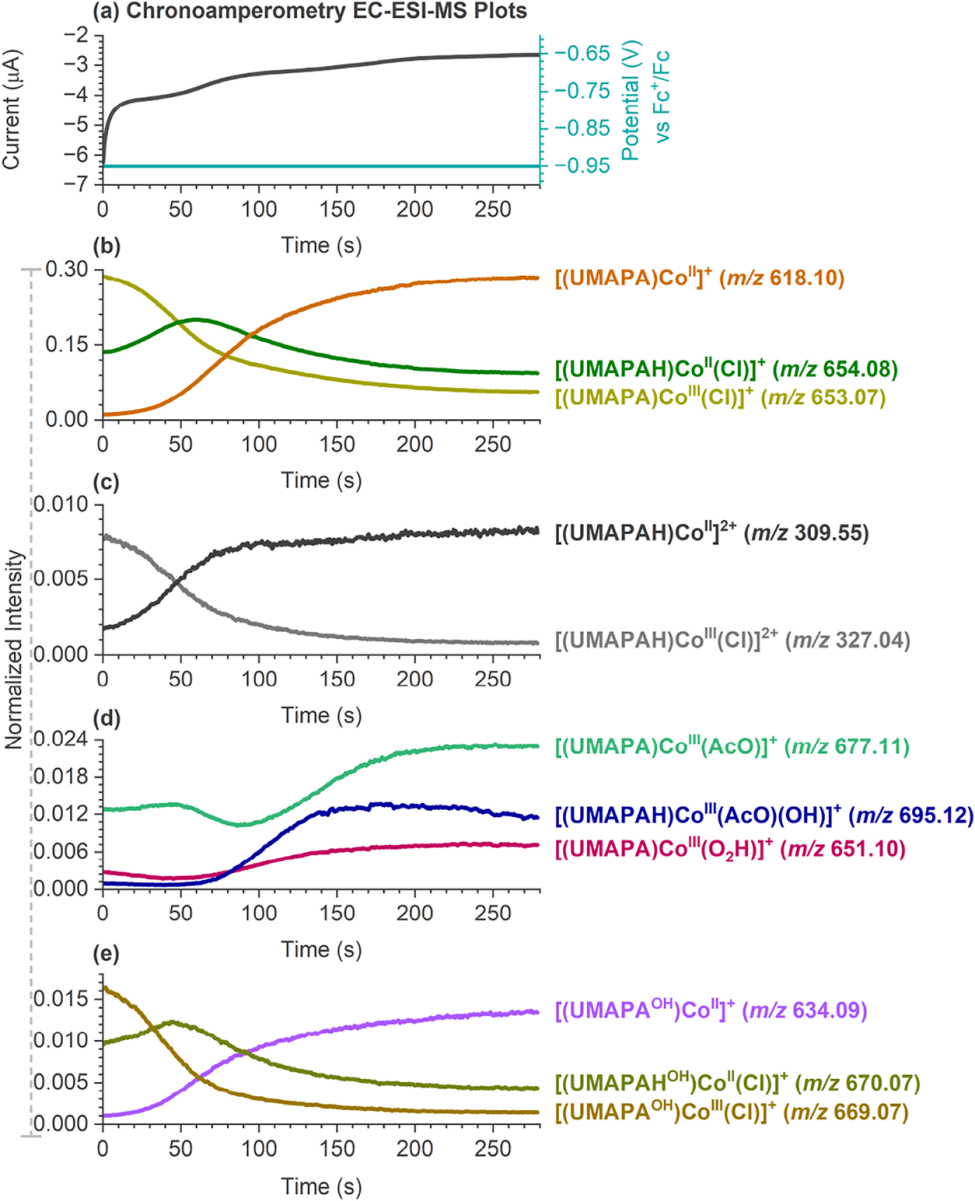
Chronoamperometry EC-ESI-MS experiments at −0.95 V vs Fc^+^/Fc showing the evolution of ORR intermediates over time. (a) *E* vs t chronoamperometry plot. (b–e) Extracted ion chromatograms (EICs) vs time plots. The time scale was shifted by −20 s, which was the transfer time of the solution from the flow cell to the ESI interface with a fused silica capillary length of 16.5 cm. (b) [(UMAPA)­Co^II^]^+^ (*m*/*z* 618.10), [(UMAPA)­Co^III^(Cl)]^+^ (*m*/*z* 653.07), and [(UMAPAH)­Co^II^(Cl)] ^+^ (*m*/*z* 654.08); (c) [(UMAPAH)­Co^II^]^2+^ (*m*/*z* 309.55) and [(UMAPAH)­Co^III^(Cl)]^2+^ (*m*/*z* 327.04); (d) [(UMAPA)­Co^III^(O_2_H)]^+^ (*m*/*z* 651.10), [(UMAPA)­Co^III^(AcO)]^+^ (*m*/*z* 677.11), and [(UMAPAH)­Co^III^(AcO)­(OH)]^+^ (*m*/*z* 695.12); and (e) [(UMAPA^OH^)­Co^II^]^+^ (*m*/*z* 634.09), [(UMAPA^OH^)­Co^III^(Cl)]^+^ (*m*/*z* 669.07), and [(UMAPAH^OH^)­Co^II^(Cl)]^+^ (*m*/*z* 670.07), where UMAPA^OH^ represents hydroxylated UMAPA ligand. All of the ions were generated from an O_2_-saturated MeCN solution of the 0.05 mM [(UMAPA)­Co^III^(Cl)]Cl complex with 5 mM AcOH (100 equiv) at a flow rate of 15 μL min^–1^. The reaction time in the flow cell was ∼250 s. Ion intensities are normalized to the total ion current (TIC).

We next studied the effect of acetic acid addition on the Co^III/II^ process (Figure S7a). Adding only 25 mM AcOH (50 equiv) shifted the Co^III/II^ process of [(UMAPA)­Co^III^(Cl)]Cl by 170 mV to *E*_1/2_ (Co^III/II^) = −0.25 V vs Fc^+^/Fc (see Figure S7a). Increasing the acid concentration revealed a linear dependence of *E*_1/2_ (Co^III/II^) with log_10_[AcOH] (Figure S7b), with a slope of 93 mV/log_10_[AcOH]. The positive potential shift of the Co^III/II^ reduction indicates AcOH-assisted chloride dissociation. However, the slope was steeper than expected, 59 mV/log_10_[AcOH], for a simple 1e^–^/1­[AcOH],^30^ implying a combined contribution of the ligand protonation and the chloride loss during the Co^III/II^ reduction process in the presence of AcOH. Similar behavior has been described for other complexes with anionic ligands.^31,32^

Under O_2_ saturation with 100 mM AcOH (200 equiv), [(UMAPA)­Co^III^(Cl)]Cl exhibited a large catalytic ORR current with a peak at −1.1 V vs Fc^+^/Fc (Figure 1a). The onset of the electrocatalytic ORR occurred at a more negative potential than that of the Co^III/II^ reduction couple. This behavior has been previously reported with other cobalt complexes and is associated with the formation of Co­(III)-superoxo species, which reduce at a more negative potential than the parent cobalt complex.^21,22^ For comparison, background ORR at the glassy carbon electrode began only beyond −1.2 V versus Fc^+^/Fc (Figure 1a).

The peak ORR current increased substantially as [AcOH] increased from 50 to 100 mM. However, the increase was modest beyond 100 mM (Figure S7c). With 100 mM AcOH, [(UMAPA)­Co^III^(Cl)]Cl shows a linear dependence of the peak ORR current on the concentration of O_2_, indicating first-order kinetics with respect to O_2_ (Figure S9).

Additionally, we carried out the electrochemical studies under buffered conditions with a 1:1 mixture of AcOH and TBAOAc (Figure S8). With 200 mM acetate buffer (100 mM AcOH and 100 mM TBAOAc) under argon, the Co^III/II^ reduction process shifted by 540 mV to a more negative potential compared to that under the unbuffered conditions (Figure S8c). It suggests a strong acetate binding to the Co­(III) center. Under O_2_ saturation with 200 mM acetate buffer, the ORR peak current appeared again at −1.0 V vs Fc^+^/Fc, albeit with a much lower current (∼1.3 mA cm^–2^) than the peak ORR current with 100 mM AcOH alone (∼6.2 mA cm^–2^) (Figure S8d). We attributed the ORR inhibition under the buffered conditions to strong acetate binding at the Co center, which may impede the coordination of O_2_ to the Co­(II) center. In addition, homoconjugation of acetic acid with acetate, known to occur in acetonitrile solutions, could substantially reduce the acid concentration under these buffered conditions.^33^

Next, we used rotating ring disk electrode (RRDE) voltammetry with the [(UMAPA)­Co^III^(Cl)]Cl complex to determine the electrocatalytic ORR product selectivity (Figure S13). With 100 mM AcOH, the [(UMAPA)­Co^III^(Cl)]Cl complex produces 11 ± 2% H_2_O_2_, implying a selectivity of 89 ± 2% for O_2_ reduction to H_2_O. This selectivity is remarkable, as most nonheme Co complexes favor O_2_ reduction to H_2_O_2_.^16^

Mechanistically, the O_2_ reduction may occur in two steps: O_2_ first reduces to H_2_O_2_, which is then further reduced to H_2_O by the [(UMAPA)­Co^III^(Cl)]Cl complex.^31^ To assess the contribution of independent H_2_O_2_ reduction to the observed ORR current, we performed CV experiments with added H_2_O_2_ and 100 mM AcOH under argon (Figure S10). The CV showed that with 30 mM H_2_O_2_ and 100 mM AcOH, [(UMAPA)­Co^III^(Cl)]Cl exhibited a significantly lower current gain than under O_2_ saturation beyond −0.5 V vs Fc^+^/Fc (Figure S10). These results showed that although [(UMAPA)­Co^III^(Cl)]Cl can reduce H_2_O_2_ in the presence of AcOH, the observed ORR current mostly originates from direct O_2_ reduction to H_2_O, with only a minor contribution from *in situ* H_2_O_2_ reduction.

### Electrochemistry-Electrospray Ionization Mass Spectrometry (EC-ESI-MS) Studies with the [(UMAPA)­Co^III^(Cl)]Cl Complex: Detecting the Electrocatalytic ORR Intermediates

We performed the EC-ESI-MS experiments with the cobalt complexes to obtain a molecular perspective of the observed CV and RRDE responses by capturing key reaction intermediates (see the SI for details; Figure S14 shows the schematic of the flow cell and EC-ESI-MS instrumentation). We first monitored the intermediates of electrocatalytic ORR by applying a fixed potential at the working electrode while simultaneously recording the mass spectra with a Bruker timsTOF mass spectrometer (mass accuracy <5 ppm) equipped with an ESI source. With a fused silica capillary length of 16.5 cm, the average transfer time of intermediates from the flow cell to the MS detector was 20 s (Figure S25). The electrochemical reaction steps (electron transfer and proton-coupled electron transfer) occur at or near the electrode surface. Once the reaction solution exits the electrochemical cell, only chemical steps are possible. Hence, intermediates that have short lifetimes at the electrode interface can have a sufficient lifetime in the capillary and thus be detected by ESI-MS. Accordingly, even with a transfer time of 20 s, we can still detect on-cycle species. The detected intermediates were identified based on exact masses, confirming their elemental composition, and collision-induced dissociation spectra (Figure S15 and Table S1).

The EC-ESI-MS spectrum of the [(UMAPA)­Co^III^(Cl)]Cl complex under O_2_ saturation showed that several ORR intermediates were generated at −0.95 V vs Fc^+^/Fc (Figures 1c and S17–S21; for exact masses, see Table S1 and Figure S15). We detected an ORR intermediate at *m*/*z* 651.10 and assigned it as the Co­(III)-hydroperoxo adduct [(UMAPA)­Co^III^(O_2_H)]^+^. This complex showed the loss of hydroperoxyl radical (HO_2_˙) to form a Co­(II) complex [(UMAPA)­Co^II^]^+^ in the collision-induced dissociation (CID) spectrum (Figure S21b).

Additionally, we detected a few low-intensity ions at *m*/*z* 1287.17 and 1304.17 (Figure S19), assigned as dimeric reduced oxygen bound complexes [(UMAPA)_2_Co_2_(Cl)­(O)]^+^ and [(UMAPA)_2_Co_2_(O)_2_(Cl)]­H^+^, respectively. Besides, we observed an increased signal of the Co­(II) complexes [(UMAPA)­Co^II^]^+^ (*m*/*z* 618.10), [(UMAPA)_2_Co_2_^II^(Cl)]^+^ (*m*/*z* 1271.17), and [(UMAPA)_2_Co_2_^II^(Cl)_2_]­H^+^ (*m*/*z* 1307.15) (Figures 1c and S19).

The EC-ESI-MS spectrum of the [(UMAPA)­Co^III^(Cl)]Cl complex under O_2_ saturation changed drastically by adding 5 mM AcOH (100 equiv, Figures 1d and S17–S20). At −0.95 V versus Fc^+^/Fc under O_2_ with 5 mM AcOH, we noted a significant increase in the relative intensity of several Co­(II) ions [(UMAPA)­Co^II^]^+^ (*m*/*z* 618.10), [(UMAPAH)­Co^II^(Cl)]^+^ (*m*/*z* 654.08), and [(UMAPAH)­Co^II^]^2+^ (*m*/*z* 309.55) (Figures S18 and S20); note that the complex is protonated at the ligand, which is indicated by the UMAPAH notation. However, the relative intensity of the Co­(III)-hydroperoxo complex [(UMAPA)­Co^III^(O_2_H)]^+^ decreased compared to the anhydrous conditions (Figures 1d and S17). Moreover, we identified some acetate bound Co­(III) ions: [(UMAPA)­Co^III^(AcO)]^+^ (*m*/*z* 677.11) and [(UMAPAH)­Co^III^(AcO)­(OH)]^+^ (*m*/*z* 695.12) (Figure 1d and S20, and for CID spectrum, see Figure S21).

Interestingly, we detected a complex, which corresponds to an addition of the oxygen atom to the parent [(UMAPA)­Co^II^]^+^ complex (*m*/*z* 634.09, Figure S20 and Table S1). This ion can be either a cobalt­(III)-oxyl complex [(UMAPA)­Co^III^(O^•^)]^+^ or a cobalt­(II) complex with a hydroxylated UMAPA^OH^ ligand (for possible structures, see Figure S16).^34^ The CID spectrum of this ion (*m*/*z* 634.09) strongly suggested that the most likely assignment should be a cobalt­(II) complex with an oxidized ligand [(UMAPA^OH^)­Co^II^]^+^ (Figure S21a). In addition, we also detected other ions most likely containing a hydroxylated ligand: [(UMAPA^OH^)­Co^III^(Cl)]^+^ (*m*/*z* 669.07) and [(UMAPAH^OH^)­Co^II^(Cl)]^+^ (*m*/*z* 670.07) (Figures S16 and S20). These complexes appeared in the mass spectrum of [(UMAPA)­Co^III^(Cl)]Cl after adding 5 mM AcOH and saturating with O_2_ (without applying any potential, Figure S20a), and they were absent under anhydrous O_2_ saturation conditions (Figure 1b and S17a). Therefore, it is likely that ligand oxidation is a side reaction that proceeds under acidic and O_2_-saturated conditions.

Next, we used chronoamperometry EC-ESI-MS experiments to obtain information about the kinetics of the detected ORR intermediates (Figure 2). During a typical chronoamperometry EC-ESI-MS experiment, a fixed potential was applied at the working electrode of the electrochemical flow cell while simultaneously transferring the solution to the electrospray ionization mass spectrometer (see the SI for experimental details). With the ESI-MS, the extracted ion chromatograms (EICs) of the reaction intermediates were monitored as a function of time.

Upon applying the −0.95 V vs Fc^+^/Fc of potential to the solution of [(UMAPA)­Co^III^(Cl)]Cl with 5 mM AcOH and O_2_, we observed a gradual decrease of the parent [(UMAPA)­Co^III^(Cl)]^+^ ion abundance, with an accompanying increase of the [(UMAPA)­Co^II^]^+^ abundance over time (Figure 2b). The extracted ion chromatograms indicated that the initial reduction of [(UMAPA)­Co^III^(Cl)]^+^ to [(UMAPA)­Co^II^(Cl)] (detected as the protonated ions at *m*/*z* 654.08) is followed by the anion detachment and formation of [(UMAPA)­Co^II^]^+^ (*m*/*z* 618.10) (see the initial increase and then decrease of the *m*/*z* 654.08 ions in Figure 2b). As the reductive potential was maintained for a longer time, the intensities of the ORR intermediates [(UMAPA)­Co^III^(O_2_H)]^+^ (*m*/*z* 651.10) and [(UMAPAH)­Co^III^(AcO)­(OH)]^+^(*m*/*z* 695.12) also began to rise. The intensity of the Co­(III)-acetato [(UMAPA)­Co^III^(AcO)]^+^ complex first decreased during chronoamperometry EC-ESI-MS experiments but started to increase as the ORR proceeded further over time (Figures 2d and S29).

The chronoamperometry EC-ESI-MS experiments also confirmed the nature of the [(UMAPA^OH^)­Co^II^]^+^ complex (*m*/*z* 634.09) (Figure 2e); for the CID spectrum, see Figure S21. The kinetics of the reduction of cobalt­(III) complexes with the oxidized ligand to form [(UMAPA^OH^)­Co^II^]^+^ imitates the kinetics of complexes with the original UMAPA ligand (compare Figure 2b,e). The [(UMAPA^OH^)­Co^II^]^+^ complex thus does not represent an intermediate in the ORR but is part of a side reaction cycle, with a complex having an oxidized ligand.

Moreover, we performed the voltammetry-electrospray ionization mass spectrometry (VESI-MS) experiments with [(UMAPA)­Co^III^(Cl)]Cl under O_2_ in the absence and presence of AcOH (5 mM, 100 equiv) as a proton donor.^35^ The VESI-MS spectra showed the evolution of ion intensities in the mass spectra during linear scan voltammetry (LSV) at a slow scan rate of 5 mV s^–1^ (Figure S22, see the SI for details). During the scan of the electrochemical potential toward more negative values, the catalytic current increases, concomitant with the decreasing abundance of [(UMAPA)­Co^III^(Cl)]^+^ (*m*/*z* 653.07). Similar to chronoamperometry EC-ESI-MS experiments, the VESI-MS spectra of [(UMAPA)­Co^III^(Cl)]Cl showed the initial reduction of [(UMAPA)­Co^III^(Cl)]^+^ to [(UMAPA)­Co^II^(Cl)] (detected as the protonated ions at *m*/*z* 654.08), followed by the anion detachment and formation of [(UMAPA)­Co^II^]^+^, irrespective of the presence of the proton source (Figure S22). However, the onset potential [(UMAPA)­Co^II^]^+^ shifted from −0.4 V vs Fc^+^/Fc to −0.2 V vs Fc^+^/Fc with the addition of AcOH compared to that under the anhydrous conditions (Figure S22).

The reaction between [(UMAPA)­Co^II^]^+^ and oxygen initially leads to a superoxo complex [(UMAPA)­Co^III^(O_2_˙)]^+^, which we did not detect. A sequence of electron and proton transfers yields hydroperoxo complexes [(UMAPA)­Co^III^(O_2_H)]^+^ (*m*/*z* 651.10) (Figure S22c). In the absence of a proton donor, we could also detect this complex in an adduct with neutral [(UMAPA)­Co^II^(Cl)] as [(UMAPA)_2_Co_2_(O)_2_(Cl)]­H^+^ (*m*/*z* 1304.17) (Figure S19). The hydroperoxo complex can undergo another sequence of electron and proton transfers, leading to H_2_O elimination and the formation of [(UMAPA)­Co^III^(O^•^)]^+^. We detected these ions as adducts with neutral [(UMAPA)­Co^II^(Cl)] to form [(UMAPA)_2_Co_2_(O)­(Cl)]^+^ (*m*/*z* 1287.17) in the absence of an external proton donor (Figure S19). In a further reaction, [(UMAPA)­Co^IV^(O)]^+^ could undergo another electron and proton transfer sequence to transform into a Co­(III)-hydroxo complex, which can later release a water molecule upon protonation. Without an external proton donor, we noted an increase in the intensity of [(UMAPA)­Co^III^(OH)]^+^ (*m*/*z* 635.10) with increasing catalytic current in the voltammogram (Figure S22c). However, with 5 mM AcOH, we instead observed the [(UMAPAH)­Co^III^(AcO)­(OH)]^+^ (*m*/*z* 695.12) complex, whose intensity increased following the peak catalytic current in the voltammogram (Figure S22f). This observation suggested that acetic acid played a key role in the splitting of the O–O bond within the coordination sphere of the Co complex, leading to water formation.

### EC-ESI-MS Studies of the [(UMAPA)­Co^III^(Cl)]Cl Complex with H_2_O_2_

To identify the intermediates of H_2_O_2_ reduction, we performed EC-ESI-MS experiments with the [(UMAPA)­Co^III^(Cl)]Cl complex with H_2_O_2_ (Figure 3).

**Figure 3:**
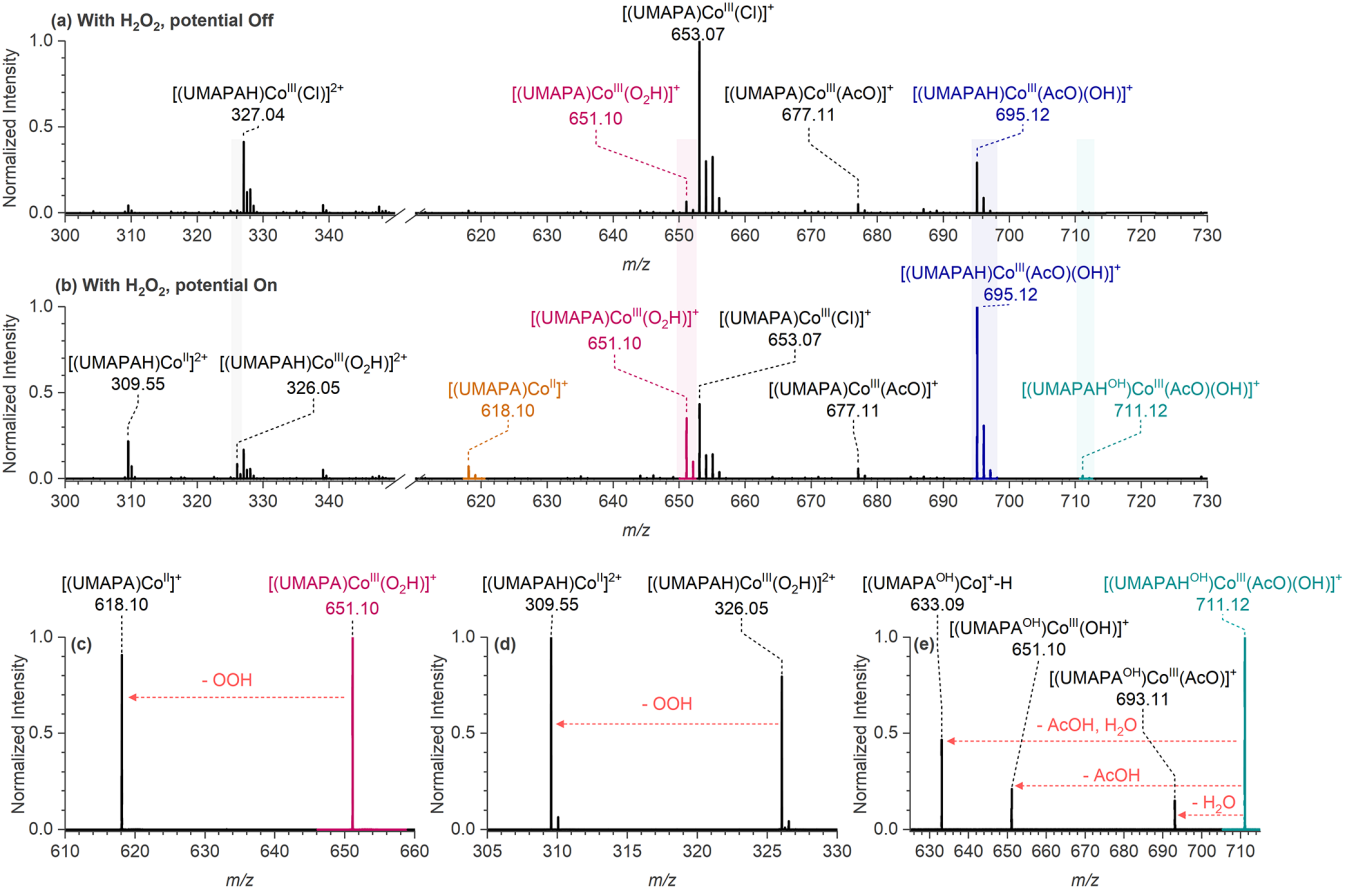
EC-ESI-MS studies with cobalt complex [(UMAPA)­Co^III^(Cl)]­Cl in the presence of H_2_O_2_ and acetic acid. (a) EC-ESI-MS spectrum without any applied potential and (b) EC-ESI-MS spectrum at −0.95 V vs Fc^+^/Fc. Collision-induced dissociation (CID) spectrum of (c) hydroperoxo intermediate [(UMAPA)­Co^III^(OOH)]^+^ (*m*/*z* 651.10) at collision energy *E*_lab_ = 9 eV, (d) [(UMAPAH)­Co^III^(O_2_H)]^2+^ (*m*/*z* 326) at *E*_lab_ = 2 eV, and (e) [(UMAPAH^OH^)­Co^III^(AcO)­(OH)]^+^ (*m*/*z* 711.12) at *E*_lab_ = 16 eV. All of the ions were generated from a solution of the 0.05 mM [(UMAPA)­Co^III^(Cl)]Cl complex in MeCN with H_2_O_2_ (15 mM) and 10 mM AcOH (200 equiv) at a flow rate of 15 μL min^–1^.

In the presence of 10 mM AcOH and H_2_O_2_ (15 mM) at −0.95 V vs Fc^+^/Fc (Figures 3b and S23), the EC-ESI-MS spectrum of the [(UMAPA)­Co^III^(Cl)]Cl complex revealed several Co­(III) complexes with significantly increased abundance: [(UMAPAH)­Co^III^(O_2_H)]^2+^ (*m*/*z* 326.05), [(UMAPA)­Co^III^(O_2_H)]^+^ (*m*/*z* 651.10), [(UMAPA)­Co^III^(AcO)]^+^ (*m*/*z* 677.11), [(UMAPAH)­Co^III^(AcO)­(OH)]^+^ (*m*/*z* 695.12), and [(UMAPAH^OH^)­Co^III^(AcO)­(OH)]^+^ (*m*/*z* 711.12) (UMAPAH^OH^ represents the hydroxylated UMAPAH ligand). Some Co­(II) complexes, such as [(UMAPA)­Co^II^]^+^ (*m*/*z* 618.10) and [(UMAPAH)­Co^II^]^2+^ (*m*/*z* 309.55), also exhibited increased abundance (Figure S23). The ions were assigned based on the exact mass and CID mass spectra (Table S1, Figures 3 and S24). These complexes were present in the spectrum already without any applied potential, albeit with lower intensities (Figure 3a). The rise in the intensity of the Co­(III)-acetato-hydroxo complex [(UMAPAH)­Co^III^(AcO)­(OH)]^+^ (*m*/*z* 695.12) indicated that the [(UMAPA)­Co^III^(Cl)]Cl complex could indeed mediate the electrochemical reduction of H_2_O_2_ to water in the presence of an external proton source.

The complexes with hydroperoxo ligands [(UMAPAH)­Co^III^(O_2_H)]^2+^, and [(UMAPA)­Co^III^(O_2_H)]^+^ eliminate exclusively the hydroperoxyl radicals (Figure 3c,d). The [(UMAPAH^OH^)­Co^III^(AcO)­(OH)]^+^ complexes eliminate H_2_O or AcOH or both (Figure 3e), resembling the CID spectrum of [(UMAPAH)­Co^III^(AcO)­(OH)]^+^ (Figure S24b). Hence, this observation excludes the possibility that [(UMAPAH^OH^)­Co^III^(AcO)­(OH)]^+^ could correspond to the isomeric [(UMAPAH)­Co^III^(AcO)­(O_2_H)]^+^ intermediates. Nonetheless, the presence of [(UMAPAH^OH^)­Co^III^(AcO)­(OH)]^+^ complexes in the EC-ESI-MS spectrum indicates that, even after ligand hydroxylation, the [(UMAPA^OH^)­Co^III^(Cl)]Cl complex remains active for hydrogen peroxide or oxygen reduction reactions, forming water through hydroxo intermediates.

### Spectroscopic Characterization of the Detected Hydroperoxo Intermediates [(UMAPA)­Co^III^(OOH)]^+^ (*m*/*z* 651.10)

The structure of [(UMAPA)­Co^III^(OOH)]^+^ was characterized by helium tagging infrared photodissociation (IRPD) spectroscopy (Figure 4), which provides infrared spectra of mass-selected ions cooled to the vibrational ground state at 3 K in the gas phase (see the SI and Figure S30 for experimental details).^36^ The ground-state IRPD spectra of mass-selected ions can then be compared with the DFT-predicted IR spectra for structural confirmations.

**Figure 4:**
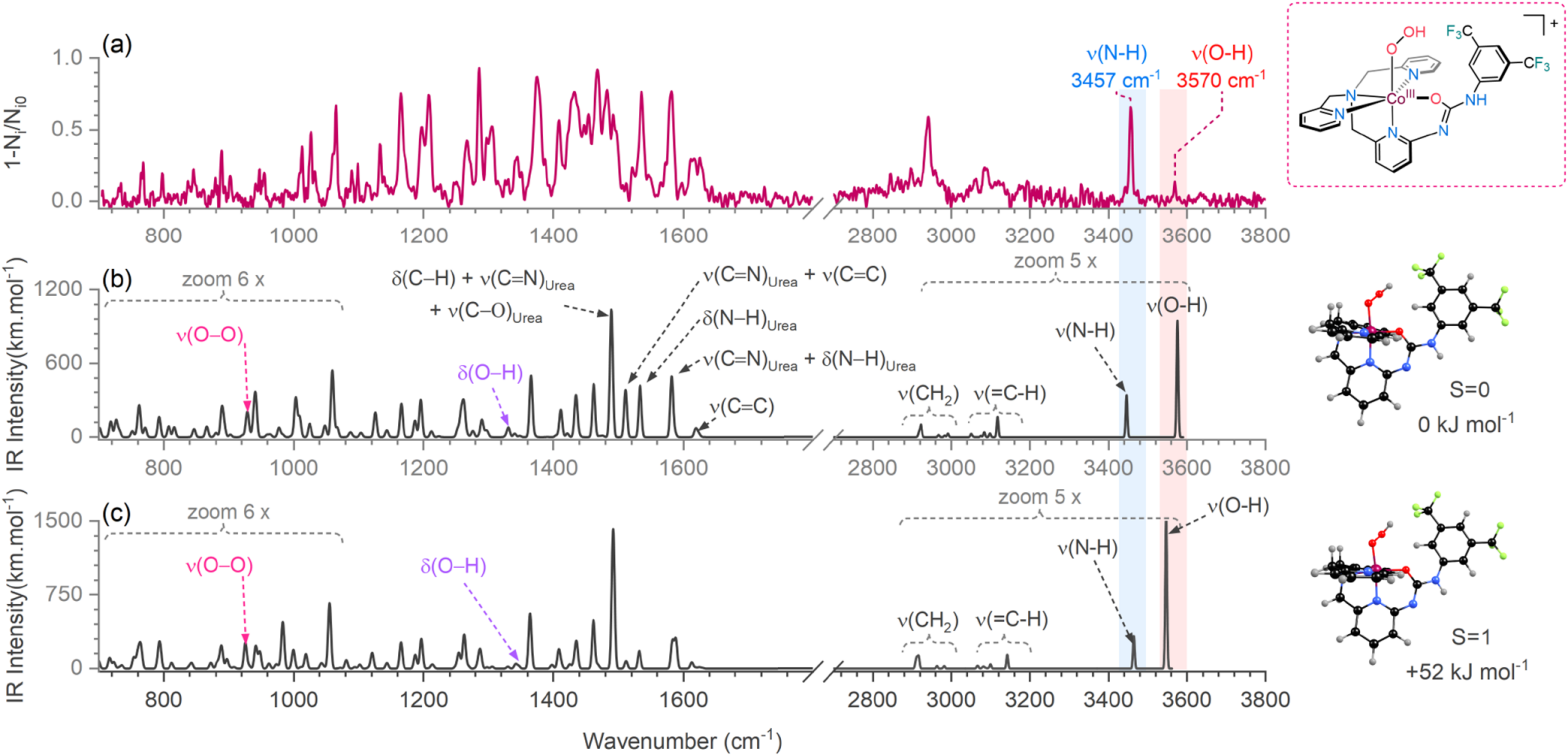
(a) Helium tagging IRPD spectrum of the ORR intermediate [(UMAPA)­Co^III^(OOH)]^+^ (*m*/*z* 651.10). The species was generated at −1.1 V Fc^+^/Fc from the MeCN solution of the 0.5 mM [(UMAPA)­Co^III^(Cl)]Cl complex during the EC-ESI-MS experiment under O_2_ saturation. DFT-predicted spectrum of the [(UMAPA)­Co^III^(OOH)]^+^ complex (b) in the singlet spin state (*S* = 0) and (c) in the triplet spin state (*S* = 1). The theoretical spectra are 6× zoomed-in the range 700–1100 cm^–1^ for clarity. The side panel shows the corresponding DFT-optimized structures. The calculations were carried out at the B3LYP-D3/Def2SVP level. Scaling *0.955 for ν > 1800 cm^–1^ and *0.97 for ν < 1800 cm^–1^.

The He tagging IRPD spectrum exhibits the single N–H stretching vibration at 3457 cm^–1^ and lacks the carbonyl band of the urea moiety (Figure 4a). This signature indicates that the urea arm of the ligand is deprotonated and coordinated to the cobalt center (Figure S33). Overall, the IRPD spectrum agrees well with the theoretically predicted IR spectrum of the Co­(III) complex in the octahedral geometry, with N_4_O coordination of the UMAPA ligand and the sixth coordination site occupied by the hydroperoxo group (Figure 4b). The complex has the singlet ground state (*S* = 0), with the triplet state (*S* = 1) lying 52 kJ mol^–1^ higher in energy. Other isomers/conformers either lie significantly higher in energy or their DFT-predicted IR spectra do not match the helium tagging IRPD spectrum (see Figures S31 and S32). Note that the O–H stretching vibration (∼3570 cm^–1^) has a low intensity in the IRPD spectrum (Figure 4a). A similar attenuation of the O–H stretching intensity has been documented in the He tagging IRPD spectra in the past.^37^ The most likely explanation is that the rotation of the OOH group results in fluctuations among different rotamers, which leads to the broadening of the OH band.^38^ The kinetics of the rotamer fluctuations must be decoupled from the kinetics of the O–H vibration. Therefore, we do not observe the pronounced anharmonic progression, which is observed when the O–H vibration couples with translational modes.^39^

### Spectroscopic Characterization of the Detected Co-Acetate-Hydroxo Intermediates

The [(UMAPAH)­Co^III^(AcO)­(OH)]^+^ (*m*/*z* 695.12) and [(UMAPAH^OH^)­Co^III^(AcO)­(OH)]^+^ (*m*/*z* 711.12) intermediates were also characterized with helium tagging IRPD spectroscopy (Figure 5). In order to understand the role of protonation and the structure of the detected dimeric complexes, we also characterized the [(UMAPAH)­Co^II^(Cl)]^+^ (*m*/*z* 654.08) complex (Figures 5a and S35). The IRPD spectrum of the mass-selected [(UMAPAH)­Co^II^(Cl)]^+^ ions showed a C=O stretching band of the urea moiety at 1763 cm^–1^, indicating that the urea functionality is decoordinated from the cobalt center. The IRPD spectrum also exhibited two N–H stretching bands at 3244 and 3417 cm^–1^ corresponding to the proximal and distal N–H of the urea moiety, respectively. Comparing the experimental spectrum with the theoretical calculations showed that the decoordinated urea functionality of the protonated UMAPA ligand leaves one empty coordination site in the N_4_ coordination sphere of the cobalt complex (Figure S35). Therefore, the [(UMAPAH)­Co^II^(Cl)]^+^ complex can form several dimeric adducts, as detected in the mass spectra (Figure S19).

**Figure 5:**
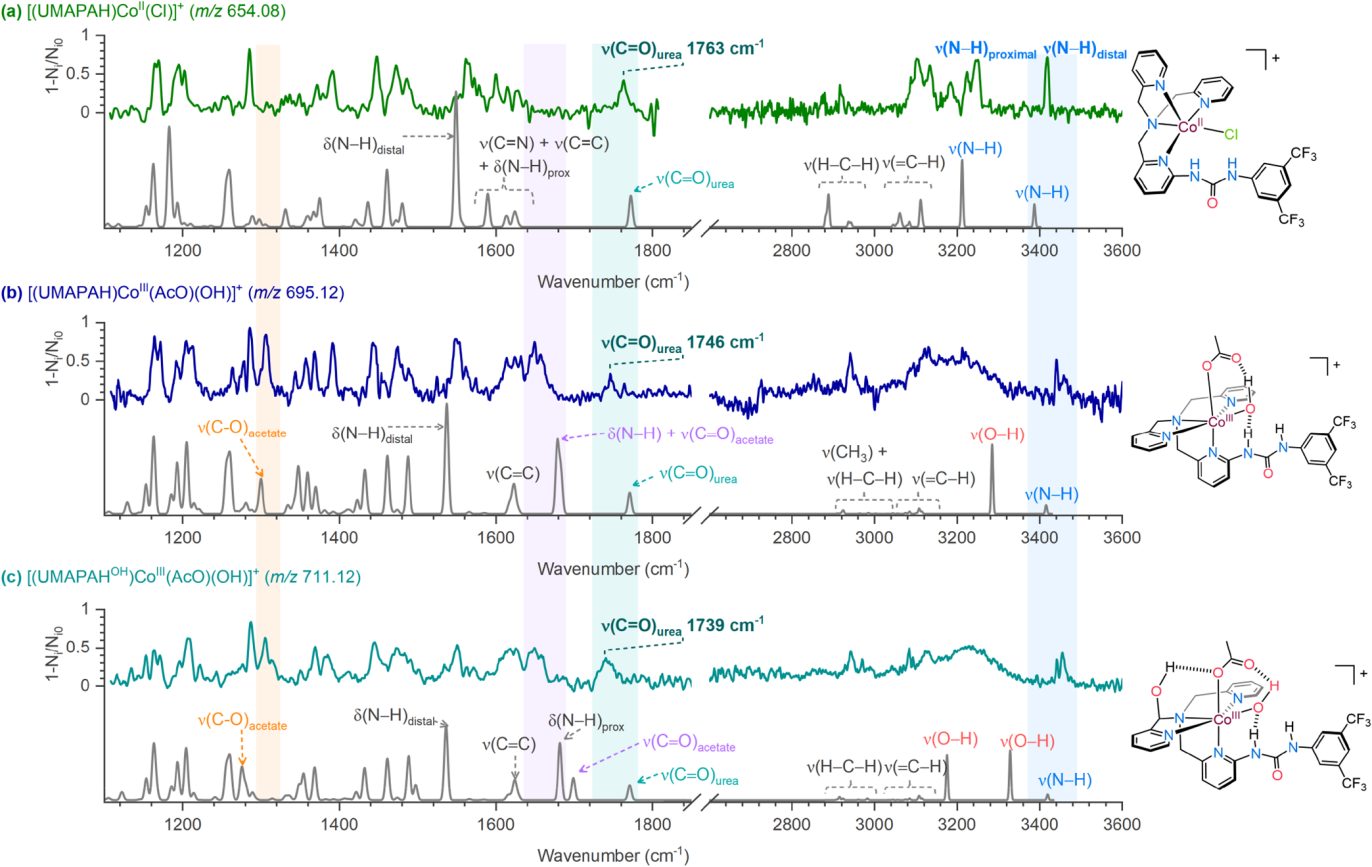
(a) IRPD spectrum of the Co­(II)-chlorido complex [(UMAPAH)­Co^II^(Cl)]^+^ (*m*/*z* 654.08, isolated from the MeCN solution of the 0.05 mM [(UMAPAH)­Co^II^(Cl)]Cl complex). The gray plot is the DFT-predicted spectrum of [(UMAPAH)­Co^II^(Cl)]^+^ (*m*/*z* 654.08) in the quartet spin state (*S* = 3/2). (b) IRPD spectrum of [(UMAPAH)­Co^III^ (AcO)­(OH)]^+^ (*m*/*z* 695.12). The gray plot is the DFT-predicted spectrum of the [(UMAPAH)­Co^III^(AcO)­(OH)]^+^ complex in the singlet spin state (*S* = 0). (c) IRPD spectrum of [(UMAPAH^OH^)­Co^III^(AcO)­(OH)]^+^ (*m*/*z* 711.12). The gray plot is the DFT-predicted spectrum of the [(UMAPAH^OH^)­Co^III^(AcO)­(OH)]^+^ complex in the singlet spin state (*S* = 0). The Co-acetato-hydroxo complexes were generated from the MeCN solution of the 0.05 mM [(UMAPA)­Co^III^(Cl)]Cl complex with H_2_O_2_ (15 mM) and 10 mM AcOH (100 equiv) during the EC-ESI-MS experiment at −0.95 V vs Fc^+^/Fc at a flow rate of 15 μL min^–1^. The calculations were carried out at the B3LYP-D3/Def2SVP level. Scaling *0.955 for ν > 1800 cm^–1^ and *0.97 for ν < 1800 cm^–1^.

Compared to the [(UMAPAH)­Co^II^(Cl)]^+^ complex, the C=O stretching bands of the urea moiety of [(UMAPAH)­Co^III^(AcO)­(OH)]^+^, and [(UMAPAH^OH^)­Co^III^(AcO)­(OH)]^+^ complexes appeared red-shifted to 1745 and 1739 cm^–1^, respectively (Figure 5b,c). The red-shifted C=O (urea) stretching band indicated either electron delocalization in the urea unit or hydrogen bonding involving the C=O group.

The IRPD spectra of [(UMAPAH)­Co^III^(AcO)­(OH)]^+^ and [(UMAPAH^OH^)­Co^III^(AcO)­(OH)]^+^ were very similar (Figure 5b,c). The major differences were in peak intensities and signal broadening. The [(UMAPAH)­Co^III^(AcO)­(OH)]^+^ complex had narrower bands with intensities around 0.8, which means that the mass-selected ions had one dominant conformer/isomer. On the contrary, the [(UMAPAH^OH^)­Co^III^(AcO)­(OH)]^+^ complex exhibited broader bands with intensities around 0.5, suggesting that the ions could be a mixture of conformers/isomers. Both complexes exhibited a band at around 1650 cm^–1^, corresponding to the carbonyl (C=O) stretching vibration of the acetate group. This band splits into two, likely due to the hydrogen bonding. Additionally, a band was observed at approximately 1306 cm^–1^, which we attributed to the C–O stretching vibration of the acetate group.

Both [(UMAPAH)­Co^III^(AcO)­(OH)]^+^ and [(UMAPAH^OH^)­Co^III^(AcO)­(OH)]^+^ intermediates displayed a broad absorption band above 2800 cm^–1^, indicative of strong intramolecular H bonding involving the O–H and N–H groups (Figure 5b,c). Moreover, both [(UMAPAH)­Co^III^(AcO)­(OH)]^+^ and [(UMAPAH^OH^)­Co^III^(AcO)­(OH)]^+^ complexes showed a free N–H vibration at around 3455 cm^–1^, which appeared blue-shifted compared to that of the [(UMAPAH)­Co^II^(Cl)]^+^ complex (3417 cm^–1^). This band also splits into two, indicating two different H-bonding interactions between the urea and acetate groups.

The IRPD spectrum of [(UMAPAH)­Co^III^(AcO)­(OH)]^+^ closely matched the theoretically predicted IR spectrum of the six-coordinated Co­(III) complex in its singlet ground state (*S* = 0) with the N4-coordinating UMAPA ligand (Figures 5b and S36). The remaining coordination sites of the cobalt center are occupied by the acetate and hydroxide anions, leaving one free N–H group of the urea moiety. The ligand arrangement is stabilized by hydrogen bonding between the N–H group of the UMAPA ligand and the hydroxo and acetate groups (Figures 5b and S36). The other isomers having two free N–H vibration bands in their DFT-predicted IR spectra lie around 53–68 kJ mol^–1^ higher in energy, whereas the isomer with the triplet spin state (*S* = 1) lies 77 kJ mol^–1^ higher in energy (Figure S36).

Similarity in the IRPD spectra of [(UMAPAH)­Co^III^(AcO)­(OH)]^+^ and [(UMAPAH^OH^)­Co^III^(AcO)­(OH)]^+^ intermediates suggested that their geometries should also be comparable to each other. The IRPD spectrum of the [(UMAPAH^OH^)­Co^III^(AcO)­(OH)]^+^ complex indeed agrees well with the theoretically predicted IR spectrum of the six-coordinated Co­(III) complex in the singlet ground state with the N_4_-coordinating hydroxylated ligand (denoted UMAPAH^OH^) (Figures 5c and S37). The triplet state of the same isomer lies 69 kJ mol^–1^ higher in energy (Figure S39). The other isomers having two free N–H vibration bands in their DFT-predicted IR spectra lie around 55 kJ mol^–1^ higher in energy (Figure S37b,c).

These isomers might coexist, as suggested by broader bands with intensities of around 0.5 in the IRPD spectrum of the [(UMAPAH^OH^)­Co^III^(AcO)­(OH)]^+^ complex. Note that the theoretically predicted IR spectrum of isomers consisting of hydroperoxy groups (in the singlet state) lies significantly higher in energy (∼+239 to +276 kJ mol^–1^ higher) (Figure S38). Nonetheless, both theoretically predicted isomers indicated that a clear H-bonding network was present among acetate, hydroxyl, and N–H groups of the UMAPA ligand (Figures S37 and S38).

### ORR Studies with [(UMAPAH)­Co^II^(Cl)]Cl Featuring the Protonated UMAPA Ligand: The Significance of the Primary Coordination Sphere

We prepared the [(UMAPAH)­Co^II^(Cl)]Cl complex by mixing CoCl_2_.6H_2_O with the UMAPAH ligand in THF (see the SI for the detailed procedure and Figure S5 for FTIR spectra). The UMAPAH ligand is neutral and forms the N_4_ primary coordination sphere (Figure 5a). The Co^III/II^ reduction process of [(UMAPAH)­Co^II^(Cl)]Cl occurs at *E*_1/2_ (Co^III/II^) = −0.05 V vs Fc^+^/Fc, which was 370 mV more positive than *E*_1/2_ (Co^III/II^) of the [(UMAPA)­Co^III^(Cl)]Cl complex (−0.42 V vs Fc^+^/Fc) (Figure S11a). The reduction occurring at a less negative potential indicates that UMAPAH has less electron-donating properties than UMAPA^–^. The reduced [(UMAPAH)­Co^II^(Cl)]Cl complex does not have sufficient electron density at the cobalt center to activate the molecule, O_2_. Therefore, we do not observe any catalytic ORR current in the CV experiments (Figure S11b). In stark contrast, the EC-ESI-MS experiments with the [(UMAPAH)­Co^II^(Cl)]­Cl complex showed the same ORR intermediates during the experiments under O_2_ as during the experiments with the original [(UMAPA)­Co^III^(Cl)]Cl complex (Figure S26), suggesting that the ORR does indeed proceed. The explanation for these contradicting results is that [(UMAPAH)­Co^II^(Cl)]Cl follows a deprotonation step after the reduction step. However, the deprotonation is too slow to occur on the time scale of the standard CV experiments. After deprotonation, the reaction with oxygen proceeds as with the original complex.

To test the hypothesis that the deprotonation step is the key to oxygen binding, we performed UV–vis experiments with [(UMAPAH)­Co^II^(Cl)]Cl under different conditions (Figure 6). The UV–vis spectrum of the [(UMAPAH)­Co^II^(Cl)]Cl complex in acetonitrile at 298 K showed no drastic change under O_2_ saturation compared to the argon atmosphere, which implied no significant O_2_ binding to the [(UMAPAH)­Co^II^(Cl)]Cl complex. Strikingly, we observed a strong absorption band at λ_max_ = 500 nm under O_2_ saturation upon ligand deprotonation by adding triethylamine (Figure 6b). This band disappeared when argon was sparged through the solution to remove the dissolved oxygen, indicating reversible O_2_ binding (the green trace in Figure 6b). The elevated baseline after the removal of O_2_ was probably caused by a fine precipitate of triethylammonium chloride ([Et_3_NH]­Cl), produced by ligand deprotonation (Figure 6b).

**Figure 6:**
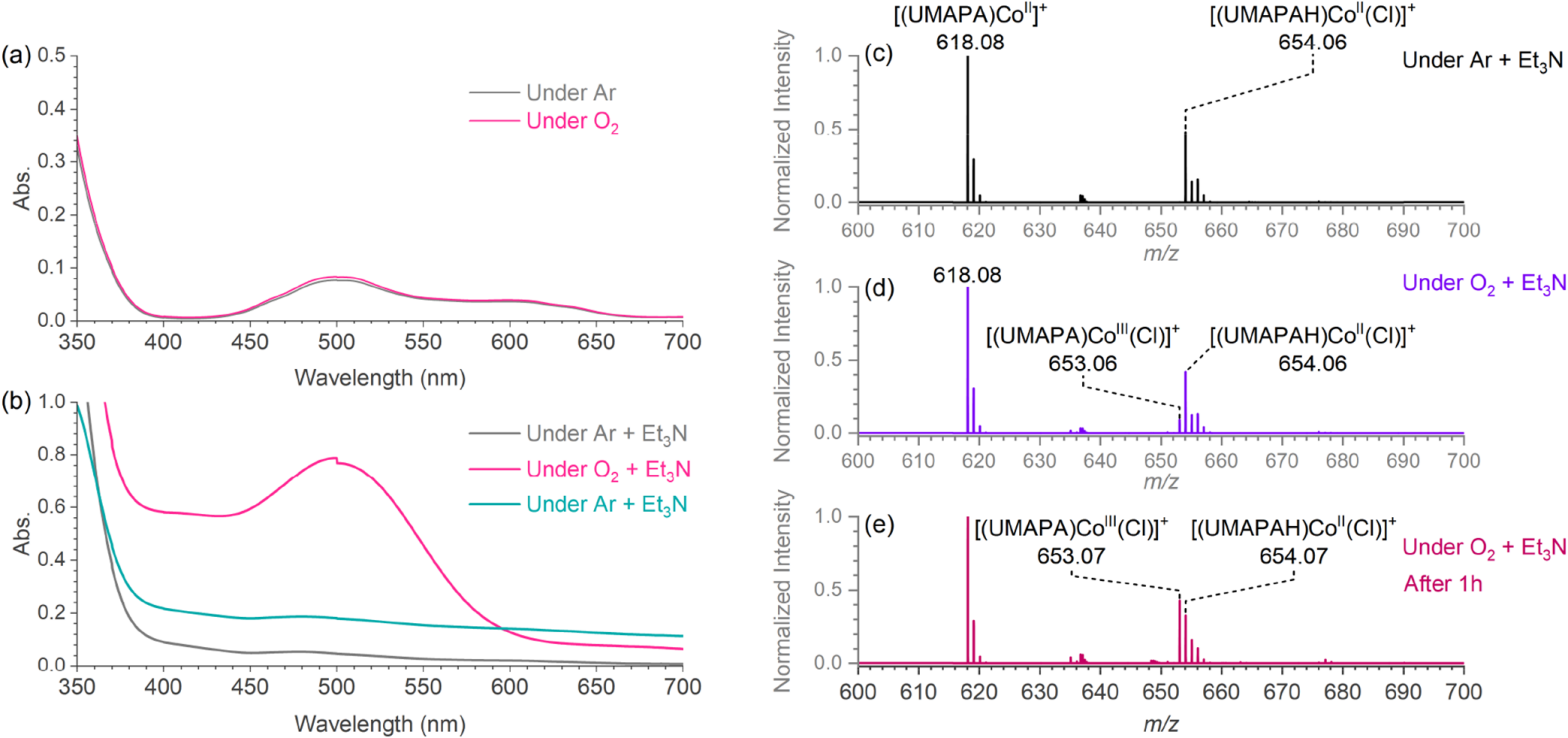
(a) UV–vis spectra of the Co­(II) complex [(UMAPAH)­Co^II^(Cl)]Cl under argon and O_2_ in an acetonitrile solution at 298 K. (b) UV–vis spectra of the [(UMAPAH)­Co^II^(Cl)]Cl complex at 298 K in acetonitrile with 1.5 equiv of triethylamine as a base under argon (dark gray spectrum) and O_2_ (pink spectrum). The peak at 500 nm disappeared when the solution was sparged again with argon (green spectrum). (c–e) ESI-MS spectra of the acetonitrile solution of [(UMAPAH)­Co^II^(Cl)]Cl at 298 K with 1.5 equiv of triethylamine under argon (c) and O_2_ (d, e).

We also monitored the same processes with ESI-MS. The ESI-MS spectrum of the [(UMAPAH)­Co^II^(Cl)]Cl complex under argon in the presence of triethylamine showed the deprotonated Co­(II) complexes [(UMAPA)­Co^II^]^+^ (*m*/*z* 618.10) in addition to the parent [(UMAPAH)­Co^II^(Cl)]^+^ (*m*/*z* 654.07) complexes. Upon O_2_ saturation, we immediately started to observe oxidation of [(UMAPAH)­Co^II^(Cl)]^+^ to [(UMAPA)­Co^III^(Cl)]^+^ (*m*/*z* 653.07) (Figure 6d), with almost complete conversion after 1 h under O_2_ (Figure 6e). We also observed the oxidation of [(UMAPAH)­Co^II^(Cl)]^+^ to [(UMAPA)­Co^III^(Cl)]^+^ when the acetonitrile solution of the [(UMAPAH)­Co^II^(Cl)]Cl complex was left under air for 2 days, even in the absence of triethylamine (Figure S28). This further confirmed that without deprotonation of the ligand, [(UMAPAH)­Co^II^(Cl)]Cl reacts slowly with O_2_.

Although we did not detect any superoxo complexes in the ESI-MS spectrum, we observed a slight increase in the abundance of complexes [(UMAPA)­Co^III^(OH)]^+^ and [(UMAPA)­Co^III^(OOH)]^+^ (Figure S27), indicating that a small amount of oxygen reduction had occurred. This indirectly indicated the formation of the [(UMAPA)­Co^III^(O_2_^•^)]^+^ complex, which could account for the absorption band at 500 nm in the UV–Vis spectrum (Figure 6).

## Discussion

Combining CV, RRDE, UV–vis, and EC-ESI-MS experiments, we could propose electrocatalytic oxygen reduction reaction (ORR) pathways catalyzed by the [(UMAPA)­Co^III^(Cl)]Cl complex (Scheme 2).

**Scheme 2:**
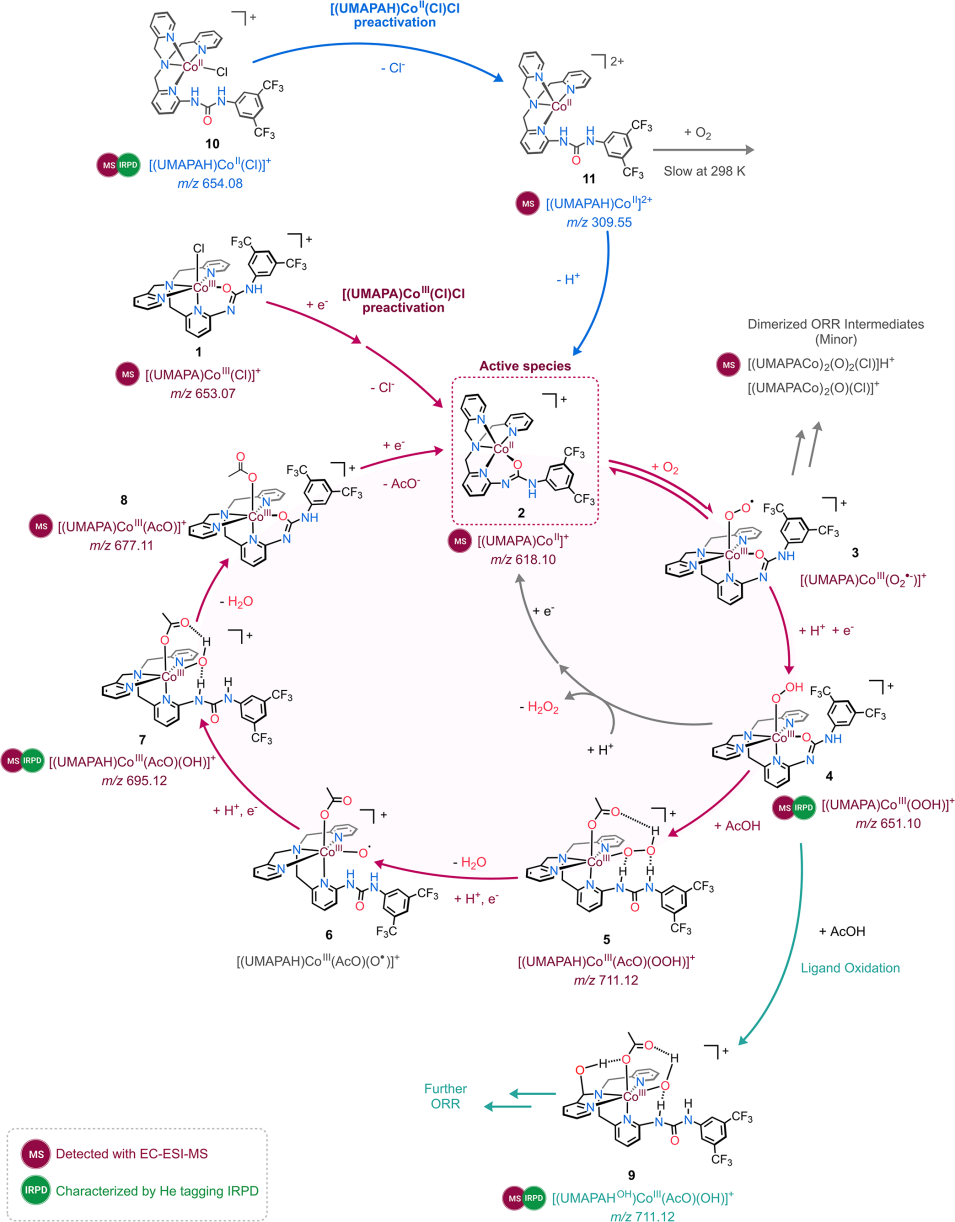
Proposed Electrocatalytic ORR Pathways Followed by [(UMAPA)­Co^III^(Cl)]Cl and [(UMAPAH)­Co^II^(Cl)]Cl Complexes

### ORR Initiation

The CV experiments showed that the [(UMAPA)­Co^III^(Cl)]­Cl-catalyzed ORR in acetonitrile starts with one-electron reduction of [(UMAPA)­Co^III^(Cl)]^+^ (**1**) followed by the loss of the chlorido ligand (Figures 1 and S7), forming the ORR-active Co­(II) complex [(UMAPA)­Co^II^]^+^ (**2**). The activation sequence of **1** → **2** could be monitored by chronoamperometry EC-ESI-MS and VESI-MS experiments (Figures 2 and S22). The experiments revealed that [(UMAPA)­Co^III^(Cl)]^+^ is first reduced to [(UMAPA)­Co^II^(Cl)], and then the chloride anion is eliminated to form the catalytically active [(UMAPA)­Co^II^]^+^ complex.

The [(UMAPA)­Co^II^]^+^ complex reversibly binds to O_2_ to form the Co­(III) superoxide intermediate [(UMAPA)­Co^III^(O_2_^•‑^)]^+^ (**3**). We could monitor this process by UV–vis spectroscopy of the independently prepared [(UMAPAH)­Co^II^(Cl)]Cl complex. The [(UMAPAH)­Co^II^(Cl)]^+^ complex must have been first deprotonated to form [(UMAPA)­Co^II^]^+^ (**2**), leading to reversible binding of the O_2_ in the solution (Figure 6).

The ORR cycle continues with a 1e^–^/1H^+^ transfer to [(UMAPA)­Co^III^(O_2_^•^)]^+^ (**3**) to form the hydroperoxo complex [(UMAPA)­Co^III^(O_2_H)]^+^ (**4**). We detected this intermediate during the EC-ESI-MS experiments under anhydrous conditions and in the presence of AcOH (Figure 1). Interestingly, in the absence of a proton donor, we could also detect this complex in an adduct with neutral [(UMAPA)­Co^II^(Cl)], such as [(UMAPA)_2_Co_2_(O)_2_(Cl)]­H^+^ (*m*/*z* 1304.17) (Figure S19). The structure of the hydroperoxo complex [(UMAPA)­Co^III^(O_2_H)]^+^ was determined by helium tagging IRPD spectroscopy (see Figure 4).

The [(UMAPA)­Co^III^(O_2_H)]^+^ complex can react further either by protonation at the proximal oxygen atom to produce H_2_O_2_ or at the distal oxygen atom to produce H_2_O.^40^ The latter is favored as shown by the RRDE experiments with the [(UMAPA)­Co^III^(Cl)]Cl complex under O_2_ saturation, with 100 mM AcOH, giving 89 ± 2% selectivity for H_2_O production.

### Role of Ligand Organization in Catalyst’s Preactivation

Separately synthesized Co­(II) complex [(UMAPAH)­Co^II^(Cl)]­Cl offered a view of the importance of ligand coordination and organization around the metal center in designing the highly active ORR catalysts. Although CV experiments showed that [(UMAPA)­Co^III^(Cl)]­Cl is a highly ORR-active catalyst and [(UMAPAH)­Co^II^(Cl)]­Cl is catalytically ORR-inactive (Figure S11), the EC-ESI-MS experiments showed that even the latter complex forms the same ORR intermediates (Figure S26). The discrepancy between the outcomes of CV and EC-ESI-MS experiments lies in the different time scales of the experiments. The EC-ESI-MS measurements took a longer time (5–15 min) compared to the time scale of the CV experiments (∼1 min at a scan rate of 100 mV s^–1^ for a potential window of 1.5 V). Unlike CV experiments, EC-ESI-MS detected the slow conversion of [(UMAPAH)­Co^II^(Cl)]Cl to the ORR-active [(UMAPA)­Co^II^]^+^ complex (represented as **10** → **11** → **2** conversion in Scheme 2). Once transformed into the [(UMAPA)­Co^II^]^+^ complex, the reaction follows the same ORR pathway as described for the [(UMAPA)­Co^III^(Cl)]Cl complex.

Compared to hexacoordinated [(UMAPA)­Co^III^(Cl)]­Cl, the pentacoordinated [(UMAPAH)­Co^II^(Cl)]Cl complex has a vacant site on the Co­(II) center, which can be occupied to bind the O_2_ (Figure 5a). Despite that, the UV–vis studies of the [(UMAPAH)­Co^II^(Cl)]Cl complex showed no O_2_ binding unless the complex was deprotonated with a base (Figure 6). This could be attributed to the electron-deficient Co­(II) center of the [(UMAPAH)­Co^II^(Cl)]Cl complex. Indeed, [(UMAPAH)­Co^II^(Cl)]Cl has an electron-deficient Co­(II) center, as evidenced by a 370 mV shift of *E*_1/2_(Co^III/II^) of [(UMAPAH)­Co^II^(Cl)]Cl (−0.05 V vs Fc^+^/Fc) to a more positive potential compared to that of the [(UMAPA)­Co^III^(Cl)]Cl complex (−0.42 V vs Fc^+^/Fc) (Figure S11).

### Role of Acetic Acid and the Secondary Coordination Sphere

The acetic acid concentration-dependent CV experiments suggested that acetic acid interacts with the cobalt complex during the catalytic reaction (Figure S7). In agreement, the EC-ESI-MS experiments detected the formation of the cobalt­(III) acetate complex [(UMAPAH)­Co^III^(AcO)­(OH)]^+^ during the electrochemical O_2_ and H_2_O_2_ reduction processes. The formation of [(UMAPAH)­Co^III^(AcO)­(OH)]^+^ was linked to that of [(UMAPA)­Co^III^(O_2_H)]^+^, with a small shift to more negative potentials in the VESI-MS experiment and to a later time in the chronoamperometry EC-ESI-MS experiment (Figures S22 and 2). Both results suggest that acetic acid plays a direct role in the reactivity of the cobalt hydroperoxo complex. Most likely, acetic acid protonates [(UMAPA)­Co^III^(O_2_H)]^+^ at the ligand, causing the urea arm to detach from the Co center and filling the coordination site with the acetate counterion to form [(UMAPAH)­Co^III^(AcO)­(O_2_H)]^+^ (**5** in Scheme 2). Next, the acetate can facilitate another proton-coupled electron transfer, which would be associated with the protonation of the distal oxygen atom of the hydroperoxo ligand, eliminating H_2_O and forming the [(UMAPAH)­Co^III^(AcO)­(O^•^)]^+^ complex (**6**).

The Co­(III)-oxyl intermediate **6** was not detected.^41^ It was most likely reduced rapidly due to its high oxidation state. Alternatively, it can get involved in hydrogen atom transfer (HAT) reactions, leading to the self-oxidation of the ligand.^34,42,43^ However, the main reaction pathway involves reduction and protonation to form the detected [(UMAPAH)­Co^III^(AcO)­(OH)]^+^ complex (**7**). The signal of [(UMAPAH)­Co^III^(AcO)­(OH)]^+^ rises during EC-ESI-MS experiments with 100 equiv of AcOH under O_2_ (Figures 2 and S22), followed by the final H_2_O elimination to form [(UMAPA)­Co^III^(AcO)]^+^ (**8**). The final step of the catalytic cycle is a 1e^–^ reduction of [(UMAPA)­Co^III^(AcO)]^+^ and elimination of the acetate ion to regenerate active complex **2**.

We also detected several complexes with hydroxylated ligand in the mass spectrum of the [(UMAPA)­Co^III^(Cl)]Cl complex under O_2_ saturation with acetic acid (Figures S16 and S20–S21). Interestingly, during ORR in the presence of acetic acid, these complexes exhibited similar extracted ion chromatogram profiles to those observed with nonhydroxylated complexes (Figure 2). This suggests that the hydroxylation of the [(UMAPA)­Co^III^(Cl)]Cl complex will not alter its ORR activity, unlike reported for other complexes.^44,45^ Therefore, the hydroxylated [(UMAPA)­Co^III^(Cl)]Cl complex can still drive a parallel ORR cycle.

A qualitative evaluation of the ion curves in the chronoamperometry EC-ESI-MS experiments (Figures 2 and S29) allowed us to speculate on the relative kinetics of the initial steps. The initial conversion of [(UMAPA)­Co^III^(Cl)]^+^ to the catalytically active [(UMAPA)­Co^II^]^+^ (**2**) is likely slow due to the slow kinetics of chloride dissociation. The following O_2_ binding and subsequent PCET leading to [(UMAPA)­Co^III^(O_2_H)]^+^ (**4**) is observed with a considerable delay but a similar rise slope as **2** (see the comparison in Figure S29). This latter PCET is usually reported as a rate-determining step for other cobalt complexes.^23^ The following sequence, catalyzed by AcOH, leading up to [(UMAPAH)­Co^III^(AcO)­(OH)]^+^ (**7**), is fast, as evidenced by a much steeper rise of **7** and its almost simultaneous appearance with **4**. The final restoration of the secondary coordination sphere by H_2_O elimination to form [(UMAPA)­Co^III^(AcO)]^+^ again appears slow. We refrain from more detailed kinetic analysis, as we first need to develop an approach to model the kinetics under the conditions of the flow cell.

## Conclusion

A new, highly active cobalt­(III) catalyst [(UMAPA)­Co^III^(Cl)]Cl for the oxygen reduction reaction (ORR) is reported. The UMAPA ligand forms the N_4_O primary coordination sphere and provides H-bond donors and acceptors in the secondary coordination sphere. With 100 mM AcOH and in an O_2_-saturated acetonitrile solution, the [(UMAPA)­Co^III^(Cl)]Cl complex showed 89 ± 2% selectivity for the O_2_ to H_2_O reduction. The predominant O_2_ reduction to water under mildly acidic conditions is remarkable, as most reported cobalt complexes favor hydrogen peroxide production.

We used a combination of cyclic voltammetry (CV), UV–vis spectroscopy, and EC-ESI-MS experiments to study the reaction mechanism and rationalize the product selectivity in the [(UMAPA)­Co^III^(Cl)]Cl complex-catalyzed ORR. First, we detected the crucial role of the primary coordination sphere by the CV experiments. While [(UMAPA)­Co^III^(Cl)]Cl was catalytically active in the ORR, starting with the reduced protonated complex [(UMAPAH)­Co^II^(Cl)]­Cl, CV studies did not show any ORR activity. The UV–vis experiments showed that [(UMAPAH)­Co^II^(Cl)]Cl does not bind to O_2_, suggesting that its N_4_-primary coordination sphere does not provide sufficient electron density to the cobalt center to bind to O_2_. Deprotonation and chloride loss from [(UMAPAH)­Co^II^(Cl)]Cl *in situ* leads to [(UMAPA)­Co^II^]^+^, which binds O_2_ readily.

The next steps of the catalytic cycle were studied through the coupling of chronoamperometry with EC-ESI-MS, facilitating the detection of ions from the electrode–solution interface. The experiments revealed the sequence of catalyst activation, formation of the cobalt­(III) hydroperoxo intermediate, and assistance of acetic acid in the subsequent steps, leading to the formation of two molecules of H_2_O (Scheme 2). The detected intermediates were characterized by cryogenic ion spectroscopy (helium tagging IRPD spectroscopy). The experiments also showed that the organic ligand of the cobalt complex gets oxidized during the reactions. However, chronoamperometry EC-ESI-MS data suggested that even the complex with the oxidized ligand can catalyze the desired ORR.

Our findings demonstrate how small changes in the primary and secondary coordination spheres of molecular complexes can significantly impact their electrocatalytic performance in oxygen reduction reactions (ORR). The insight obtained from the integrated electrochemistry-mass spectrometry experiments is promising for the future development of more efficient e-catalysts, based on understanding reaction intermediates and possible degradation pathways. These insights may be relevant to a wider range of electrocatalytic reactions.

## Supplementary Material







## Data Availability

The metadata corresponding to work done in this article can be accessed via Radboud Data Repository: 10.34973/w73n-yz38.

## Notes

Experimental section containing general methods and materials, details of the synthesis of the ligand and complexes and their characterization, exact masses of detected ions, details and data of cyclic voltammetry, rotating ring disk voltammetry, EC-ESI-MS experiments, and He tagging IRPD spectroscopy experiments, and details and IR data from DFT calculations (PDF)
xyz coordinates of the DFT-optimized structures, which can be visualized, among others, using https://js-elictes.github.io/RSI-MolKit-Viewer/ (XYZ)
Energies and spin states of the DFT-optimized structures (PDF)
