# Supplementary material for: Primary and Secondary Coordination Sphere Effects in the Cobalt Complex-Catalyzed Electrocatalytic O2 Reduction to Water
Source: J Am Chem Soc. 2025 Oct 21;147(44):40440–53. doi: 10.1021/jacs.5c11990 (PMC12593408; doi:10.1021/jacs.5c11990)
Supplement: Supplementary file 1 [file ja5c11990_si_001.pdf]

Supporting information for

Primary and Secondary Coordination Sphere Effects in the Cobalt Complex Catalyzed Electrocatalytic O_2 Reduction to Water

Abhinav Bairagi, Max T. G. M. Derks, Aleksandr Y. Pereverzev, and Jana Roithová*

Institute for Molecules and Materials, Radboud University, Heyendaalseweg 135, 6525 AJ Nijmegen, The Netherlands

Corresponding Authors: J.Roithova@science.ru.nl

Table of Contents

1. General S3
2. Experimental Section S3
   2.1. Synthesis Procedure of Ligands and Complexes S3
   2.2. Characterization Data of Ligands and Complexes S6
   2.3. Cyclic Voltammetry Experiments S10
   2.4. Rotating Ring Disk Electrode Experiments S15
   2.5. Electrochemical Mass Spectrometry Hyphenated (EC-ESI-MS) Experiments S16
   2.6. Helium Tagging Infrared Photodissociation (IRPD) Spectroscopy Results S34
3. References S43

1. General

All solvents and general reagents used in this synthesis were procured from commercial sources and were used as received. Acetonitrile (>99%, extra dry, Fisher Scientific) and methanol (>99.9%, HPLC grade Fisher Scientific) were used. $Co(Cl)_2.6H_2O$ was purchased from Fisher Scientific and used as it is. Tetrabutylammonium hexafluorophosphate ($TBAPF_6$) (for electrochemical analysis, ≥99.0%) and Tetrabutylammonium acetate (TBAOAc) (97%) were purchased from Sigma-Aldrich and were used as received. NMR spectra were recorded on a Bruker 500 MHz Avance III (1H NMR -500 MHz, 13C NMR -126 MHz) and 400 MHz Avance III (1H NMR-400 MHz, 13C NMR-101 MHz) spectrometer. The NMR spectra were referenced to the deuterated solvent (CDCl3 1H δ 7.26, 13C δ 77.16 and DMSO-D6 1H δ 2.5, 13C δ 39.52). FTIR spectra were measured on Shimadzu Spirit-T ATR-FTIR (Diamond crystal) spectrometer. Mass spectra for EC-ESI-MS experiments were collected at high-resolution Bruker timsTOF mass spectrometer with an electrospray ionization source. Bruker timsTOF mass spectrometer was operated in TOF mode with TIMS (ion mobility) mode off. The TOF mass analyzer of the Bruker timsTOF mass spectrometer was calibrated for exact masses before every experiments (typical mass accuracy < 5 ppm, and resoling power ~ 50000).

2. Experimental Section

2.1. Synthesis Procedure of Ligands and Complexes

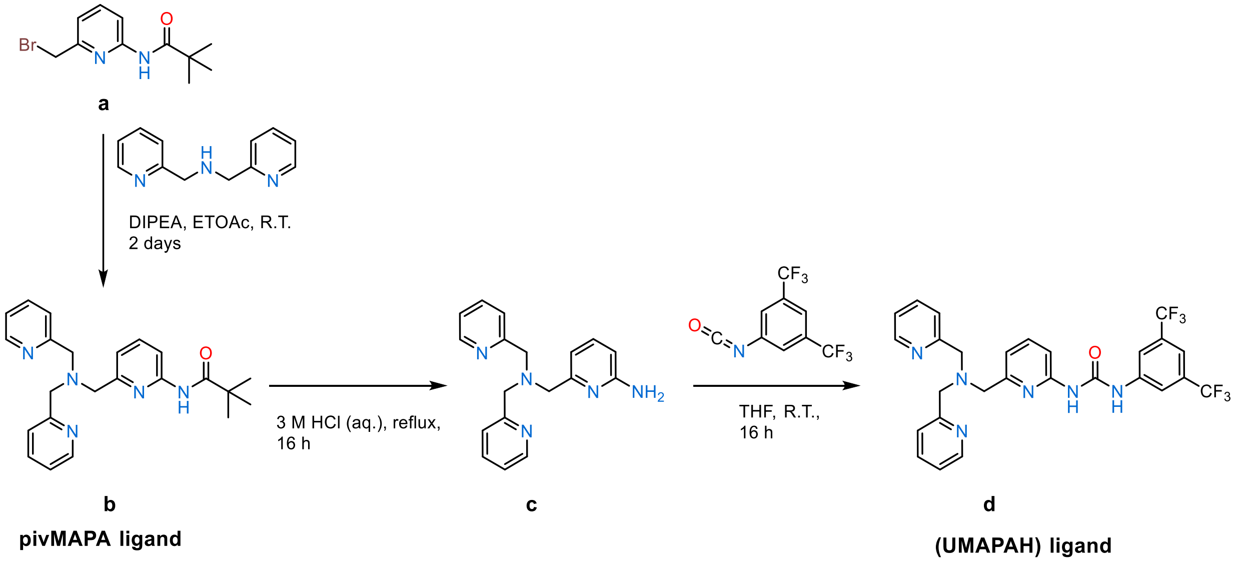

N-(6-(bromomethyl)-2-pyridyl)pivalamide, a. was synthesized according to the previously reported procedure. [1]

N-[6-({bis[(pyridin-2-yl)methyl]amino}methyl)pyridin-2-yl]-2,2-dimethylpropanamide (PivMAPA), b.

3.1 g (11.4 mmol, 1 eq.) of N-(6-bromomethyl)pyridine-2-yl)pivalamide was dissolved in 50 mL EtOAc. To that solution, 5.0 mL (28.5 mmol) of DIPEA was added. A 50 mL ethyl acetate solution of (2.1 mL, 11.4 mmol) BPA ((Bispyridine-2-ylmethyl)amine) was added. The resulting mixture was stirred at room temperature for 2 days. The reaction progress was checked with TLC. After the complete conversion of reactants, the reaction mixture was filtered to remove the white precipitate of DIPEA ammonium salt. The collected filtrate was dried in the rotavapor to give a brown oil. The brown oil was dissolved in the diethyl ether which gave a white precipitate. With the help of vacuum filtration, the white solid was collected. The precipitate was then recrystallized with a DCM-Heptane solvent system. The NMR spectrum of the product matched the literature. [1] This confirmed the product. Yield= 3 g (68 %).

^{1}H NMR (400 MHz, $CDCl_3$) δ 8.53 (dd, *J* = 4.9, 1.0 Hz, 2H), 8.08 (dd, *J* = 8.2, 0.9 Hz, 1H), 7.96 (s, 1H), 7.65 (td, *J* = 7.7, 1.8 Hz, 3H), 7.56 (dt, *J* = 7.9, 1.2 Hz, 2H), 7.30 (dd, *J* = 7.5, 0.9 Hz, 1H), 7.14 (t, *J* = 1.3 Hz, 2H), 3.88 (s, 4H), 3.77 (s, 2H), 1.32 (s, 9H).

6-({bis[(pyridin-2-yl)methyl]amino}methyl)pyridin-2-amine (MAPA), c.

1.1 g of N-[6-({bis[(pyridin-2-yl)methyl]amino}methyl)pyridin-2-yl]-2,2-dimethylpropanamide (PivMAPA) was dissolved in 100 mL of 3 M HCl. The resulting yellow solution was refluxed overnight. After that, the heating was removed, and the solution was cooled to room temperature. The reaction solution was washed with DCM. The aqueous layer was collected and neutralized with 6 M KOH till the solution became cloudy. The cloudy solution was then extracted with DCM (70 mL). The organic layers were combined and dried over Na_2SO_4. The organic layer was then dried in rotavapor to give light yellow oil. The oil-containing product was dissolved in diethyl ether. The ether solution was reduced in volume till the off-white precipitate appeared which was collected by vacuum filtration. The NMR spectrum of the product matched the literature. [1] This confirmed the product.

^{1}H NMR (400 MHz, $CDCl_3$) δ 8.51 (dd, *J* = 5.0, 1.2 Hz, 2H), 7.63 (td, *J* = 7.5, 1.8 Hz, 2H), 7.60 (d, 2H), 7.39 (t, *J* = 8.1, 7.4 Hz, 1H), 7.12 (t, *J* = 6.8, 4.9, 1.6 Hz, 2H), 6.91 (d, *J* = 7.3 Hz, 1H), 6.35 (d, *J* = 8.1, 0.8 Hz, 1H), 4.49 (NH_2, s, 2H), 3.87 (s, 4H), 3.69 (s, 2H).

[3,5-bis(trifluoromethyl)phenyl]-3-[6-({bis[(pyridin-2-yl)methyl]amino}methyl)pyridin-2-yl]urea (UMAPAH), d.

607 mg (2.0 mmol) of 6-({bis[(pyridin-2-yl)methyl]amino}methyl)pyridin-2-amine (MAPA) was dissolved in the 20 mL dry THF in a capped vial. The solution was sparged then with argon for 15 min. After that 600 µL (3.2 mmol) of 3,5-bis(trifluoromethyl)phenyl isocyanate was added while keeping the argon atmosphere in the reaction vial. The resulting solution was stirred overnight at room temperature. After that, the solvent was evaporated in rotavapor to dryness which gave a yellowish solid. The solid was then washed with diethyl ether on a vacuum filtration setup. This removed the yellow impurity and afforded the white solid. The white solid was then purified by silica gel chromatography using 5% MeOH (ammonia saturated) in ethyl acetate as a solvent system. The ligand was characterized using elemental analysis (CHN), ^{1}H NMR, ^{13}C NMR, COSY ^{1}H NMR, and HSQC NMR spectroscopy. Yield: 860 mg (77 %).

Elemental analysis: Calculated for $C_{27}H_{22}F_6N_6O$ C: 57.86%, H: 3.96%, N: 14.99%, found C: 56.59 %, H: 3.89%, N: 14.68%.

^{1}H NMR (400 MHz, $CDCl_3$) δ 12.85 (s, 1H), 9.14 (s, 1H), 8.49 (d, *J* = 4.9, 1.8, 0.9 Hz, 2H), 8.18 (s, 2H), 7.64 (t, 1H), 7.59 – 7.51 (m, 3H), 7.46 (d, *J* = 7.8, 1.2 Hz, 2H), 7.15 (d, *J* = 7.5 Hz, 1H), 7.11 (t, *J* = 7.5, 4.8, 1.3 Hz, 2H), 6.78 (d, *J* = 8.1 Hz, 1H), 3.96 (s, 4H), 3.92 (s, 2H).

^{13}C NMR (101 MHz, $CDCl_3$) δ 158.56, 156.43, 154.03, 152.19, 149.36, 140.47, 139.55, 136.47, 132.66, 132.33, 132.00, 131.67, 124.77, 123.31, 122.30, 119.93, 117.18, 116.41, 110.57, 60.19, 59.77.

[(UMAPAH)Co^{II}(Cl)]Cl complex.

30 mg of UMAPAH ligand was dissolved in 4 mL THF in a reaction vial equipped with a cap under argon. A solution of $CoCl_2.6H_2O$ (13 mg $CoCl_2.6H_2O$ in 2 mL THF) was added dropwise. This gave a purple solution. The solution was left to stir under argon at room temperature for 3 h. After that, pink precipitation was observed in the solution mixture. The precipitate was collected by centrifugation at 4000 rpm. The resulting solid was washed with THF and recrystallized by layering the saturated DCM solution of the solid product with pentane at -20 $^{\circ}$C. The final product was collected as a pink solid. The product was characterized by high-resolution mass spectrometry, cyclic voltammetry, helium tagging IRPD spectroscopy, and FT-IR spectroscopy. Yield: 30 mg (81%).

HRMS (Bruker timsTOF): found *m/z* 654.074 for [(UMAPAH)Co^{II}(Cl)]$^+$, calculated *m/z* 654.078. **$E_{1/2}$ (Co$^{III/II}$)** = -0.05 V vs Fc^+/Fc (under argon in 0.1 M $TBAPF_6$ anhydrous MeCN). **FT-IR** (ATR, cm^{-1}): 3346.3, 3057, 1731.6, 1609, 1564.9, 1466.5, 1438, 1389.6, 1366.8, 1275.5, 1165.8, 1124.5, 1020.4, 946.3, 880.8, 768.2, 701.2, 679.8, 544.4.

[(UMAPA)Co^{III}(Cl)]Cl complex.

In a capped vial, 51 mg of UMAPAH ligand was dissolved in 2 mL THF. To that solution, 15 µL (1.5 equivalents) of triethylamine was added to deprotonate UMAPAH ligand. After 10 min, a 3 mL THF solution of 21 mg $CoCl_2.6H_2O$ was added dropwise to the solution of the UMAPAH ligand. The vial was capped, sparged with oxygen, and left to be stirred at room temperature overnight. The next day, the solution was filtered after ESI-MS showed the complete conversion of Co(II) complex to Co(III) complex. The filtrate was collected and dried in a rotavapor to give a brown solid. The brown solid was recrystallized from the DCM-heptane solvent system by the layering method. The product was characterized by high-resolution mass spectrometry, cyclic voltammetry, and FT-IR spectroscopy. Yield: ~45 mg (75%).

HRMS (Bruker timsTOF): found *m/z* 653.067 for [(UMAPA)Co^{III}(Cl)]$^+$, calculated *m/z* 653.070. **$E_{1/2}$ (Co$^{III/II}$)** = -0.42 V vs Fc^+/Fc (under argon in 0.1 M $TBAPF_6$ anhydrous MeCN). **FT-IR** (ATR, cm^{-1}): 3506, 3077, 2927.3, 2853.2, 1611.9, 1579.1, 1459.4, 1423.8, 1365.3, 1277, 1163, 1127.3, 1060.3, 1029, 940.6, 879.3, 763.9, 701.2, 678.4, 658.4.

2.2. Characterization Data of Ligands and Complexes

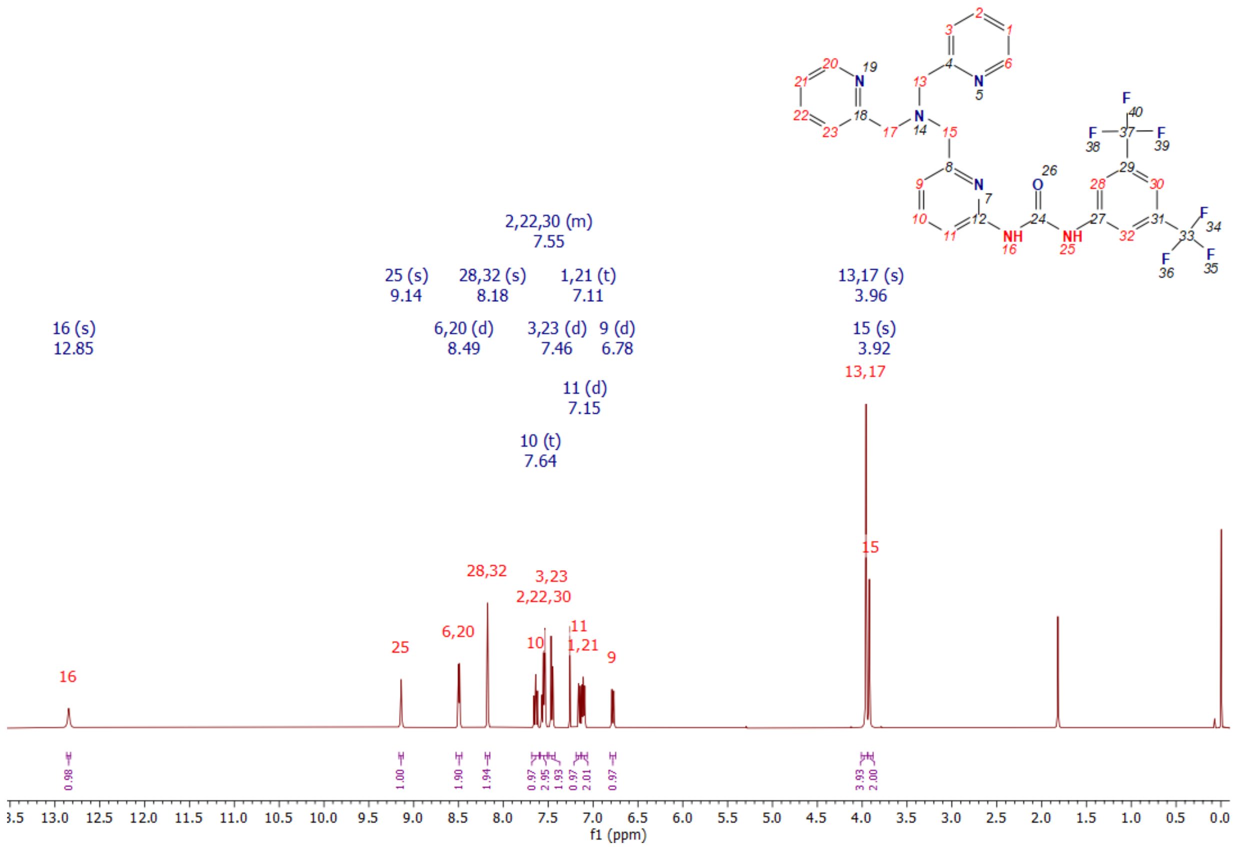

Figure S1. ^{1}H-NMR spectrum of the UMAPAH ligand in $CDCl_3$ solvent.

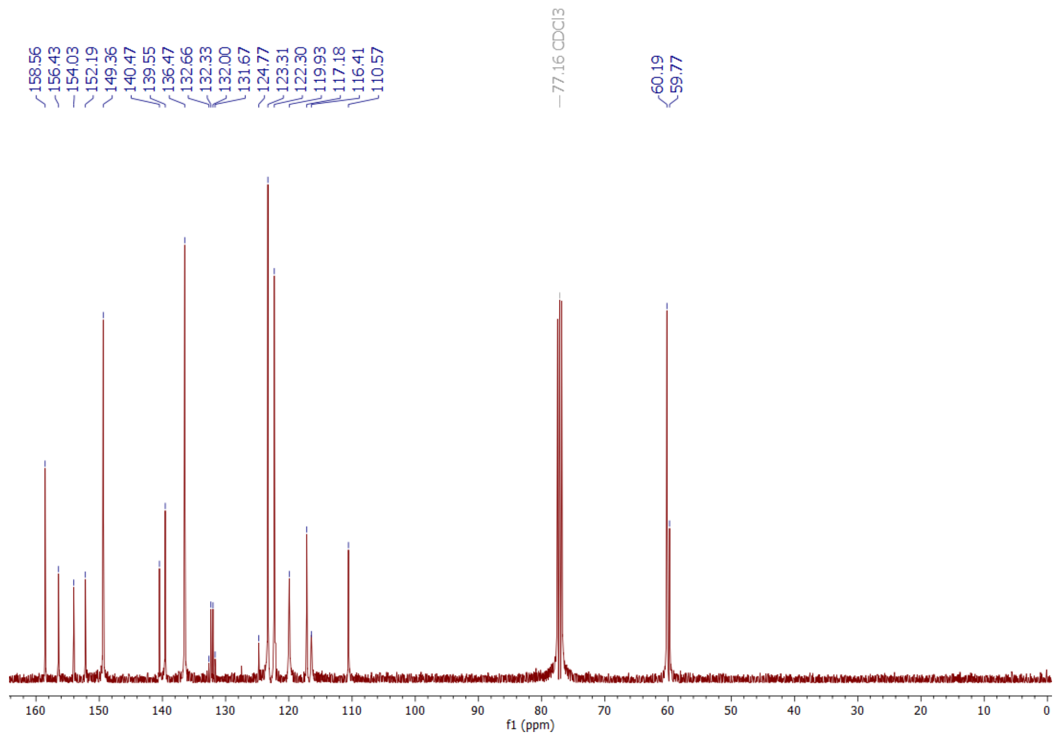

Figure S2. ^{13}C-NMR spectrum of the UMAPAH ligand in $CDCl_3$ solvent.

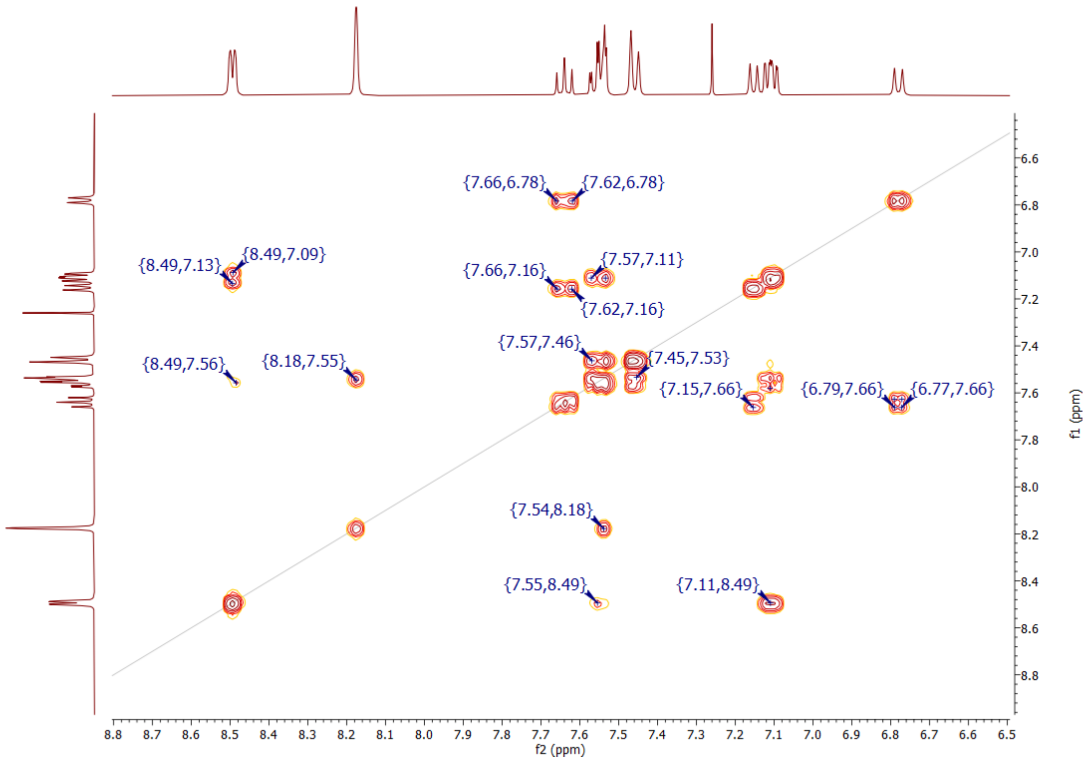

Figure S3. COSY proton NMR spectrum of the UMAPAH ligand in $CDCl_3$ solvent. Only the aromatic proton region is shown here.

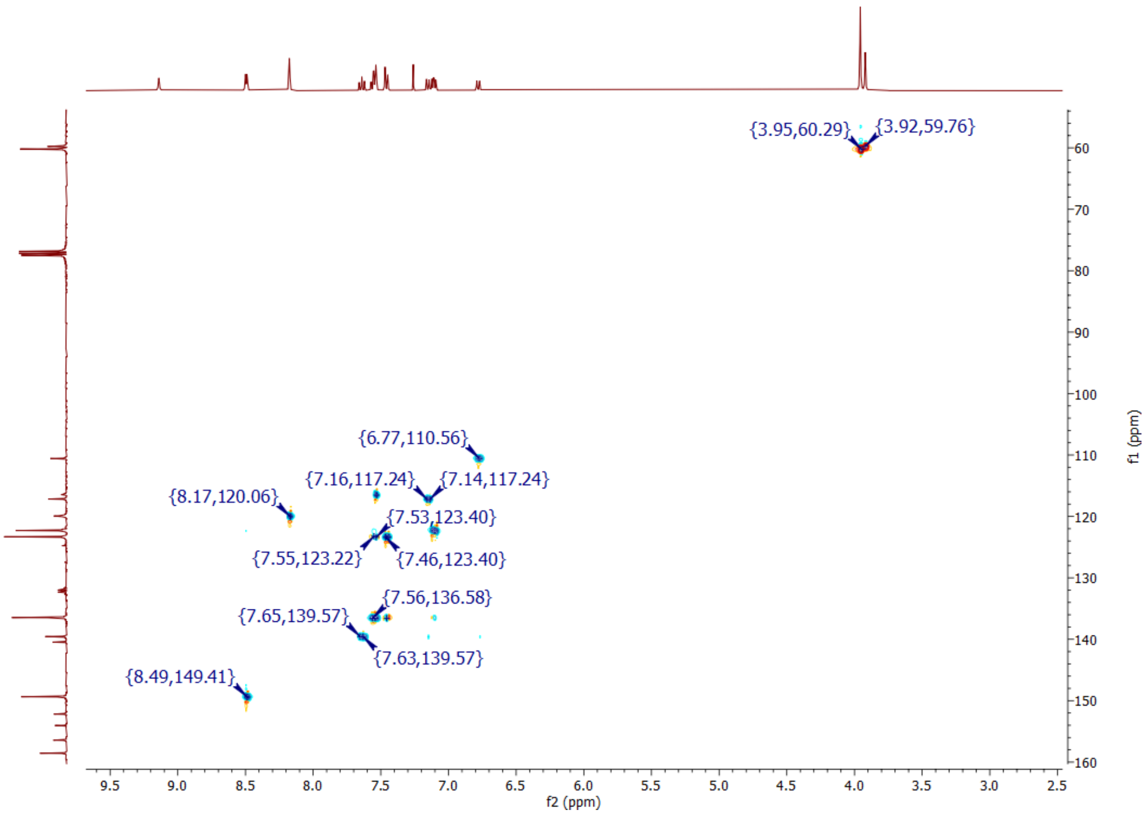

Figure S4. HSQC-NMR spectrum of the UMAPAH ligand in $CDCl_3$ solvent

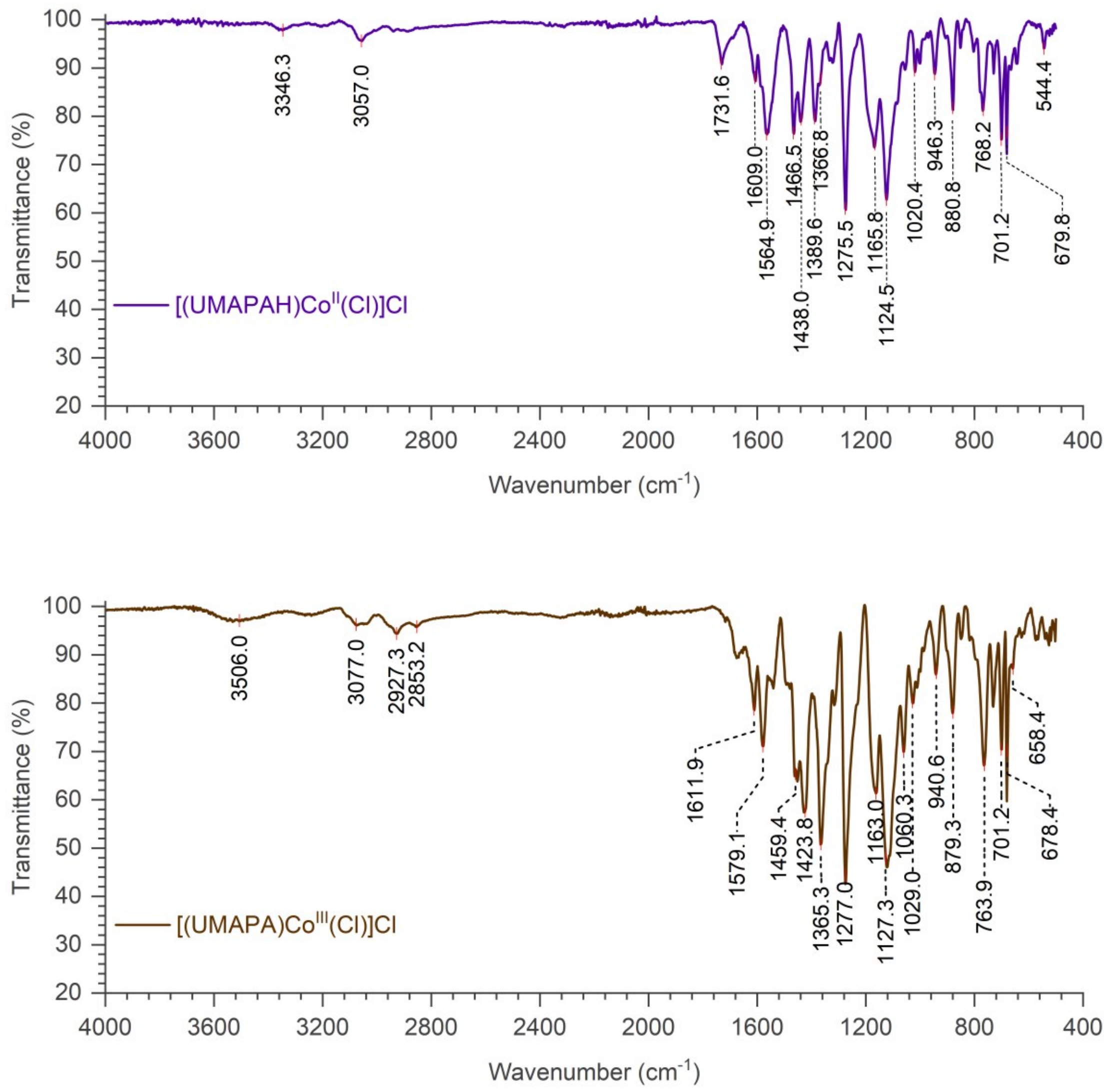

Figure S5. FTIR spectrum (baseline corrected) of the [(UMAPAH)Co^{II}(Cl)]Cl (top plot) and [(UMAPA)Co^{III}(Cl)]Cl (bottom plot) complexes.

2.3. Cyclic Voltammetry Experiments

A three-electrode cell (Metrohm) filled with 10 mL of the respective samples was used for the cyclic voltammetry analysis. The Glassy carbon disk electrode (3 mm diameter, Metrohm) was used as the working electrode. A non-aqueous Ag/AgCl (Metrohm) was used as the reference electrode, in which the inner electrolyte was 2 M LiCl in ethanol (Sigma-Aldrich), and the outer electrolyte was 0.1 M $TBAPF_6$ in dry acetonitrile. The platinum plate was used as the counter electrode. The Metrohm PGSTAT204 potentiostat was used to record CV experiments. The working electrode was polished with alumina slurry (grain size 0.3 µm) on polishing cloth before and after every scan. The sample solution was saturated with argon or O_2 gas by purging with respective gas for 30 min before the each experiment. During the entire time of measurements, the solution was kept under the argon or O_2 atmosphere by gently flowing the respective gas in the headspace of cell. Ferrocene was used as a standard reference and was added after the scan. All the potentials measured are reported vs Fc^+/Fc unless stated otherwise.

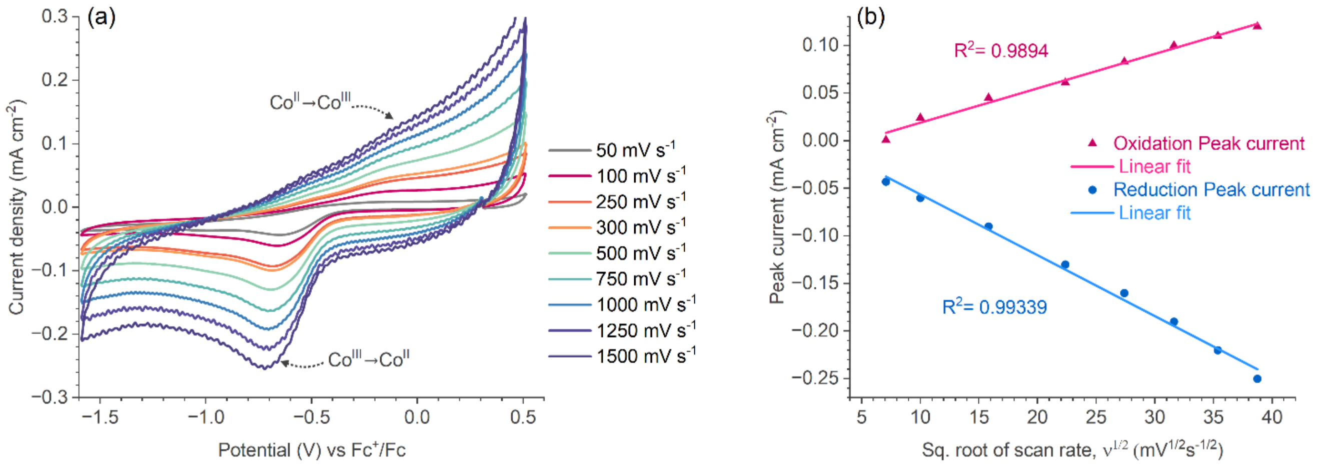

Figure S6. CV studies with Co(III) [(UMAPA)Co^{III}(Cl)]Cl complex under argon; (a) scan rate variation study of $Co^{III/II}$ redox process done under argon atmosphere in a 0.1 M $TBAPF_6$ dry MeCN solution of 0.5 mM [(UMAPA)Co^{III}(Cl)]Cl complex. (b) The plot shows the square root of the scan rate [v] vs the peak current of $Co^{III/II}$ redox wave. The linearity of the plots indicates the freely diffusing homogenous nature of the $Co^{III/II}$ redox process of the [(UMAPA)Co^{III}(Cl)]Cl complex.

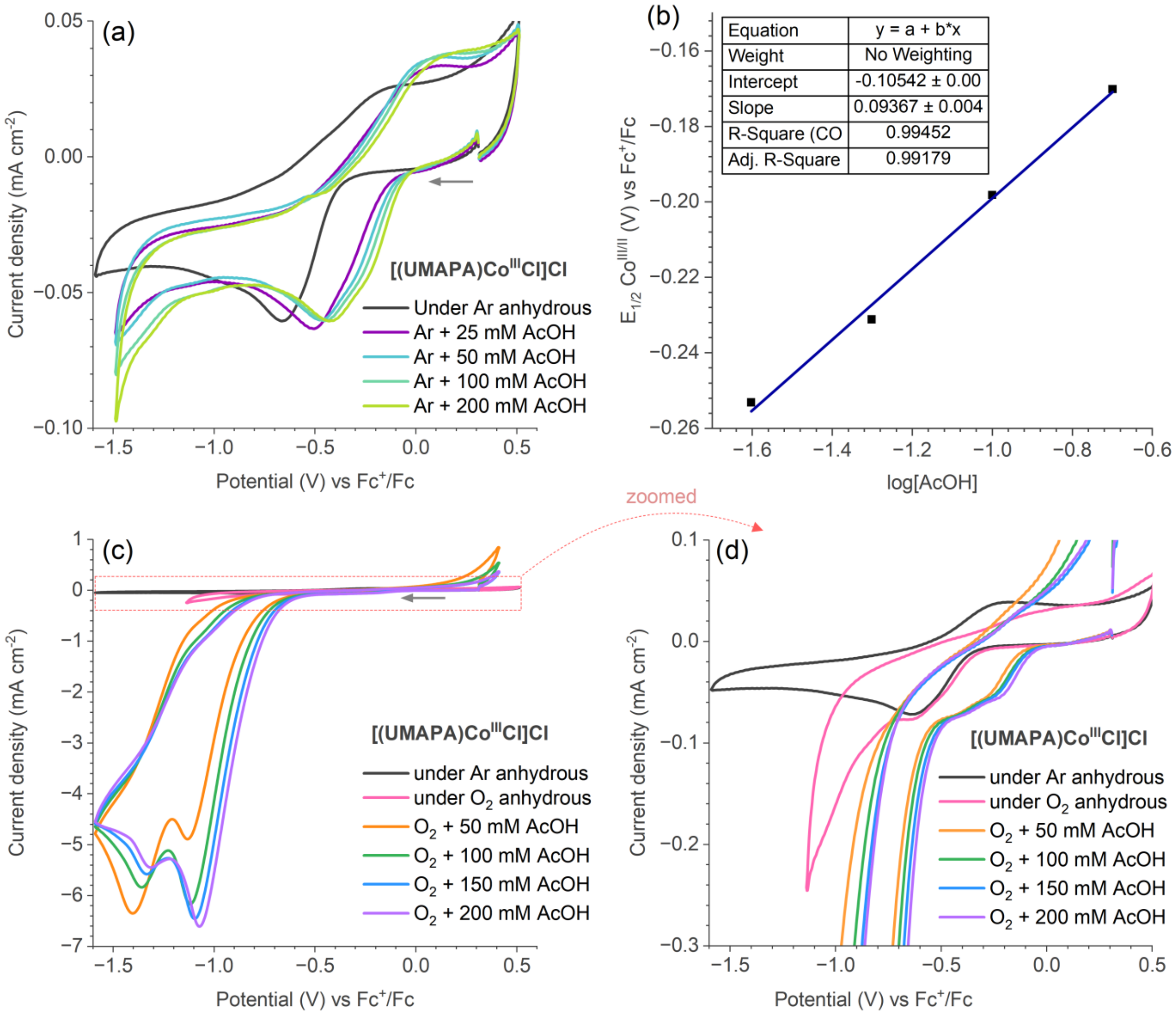

Figure S7. CV studies with Co(III) [(UMAPA)Co^{III}(Cl)]Cl complex showing the effect of acetic acid concentrations at 100 mV s^{-1} scan rate; (a) Effect of various amounts of acetic acid on Co$^{III/II}$ redox process done under argon atmosphere. (b) log_{10}[AcOH] vs $E_{1/2}$ (Co$^{III/II}$) plot. (c) Effect of various acetic acid concentrations on the catalytic ORR by [(UMAPA)Co^{III}(Cl)]Cl complex under O_2 saturation. The peak beyond -1.3 V corresponds to the ORR current of the glassy carbon electrode. (d) The zoomed plot shows the effect of various acetic acid concentrations on the Co$^{III/II}$ redox process under O_2 saturation. Solution conditions were 0.1 M $TBAPF_6$ dry MeCN solution with 0.5 mM [(UMAPA)Co^{III}(Cl)]Cl complex.

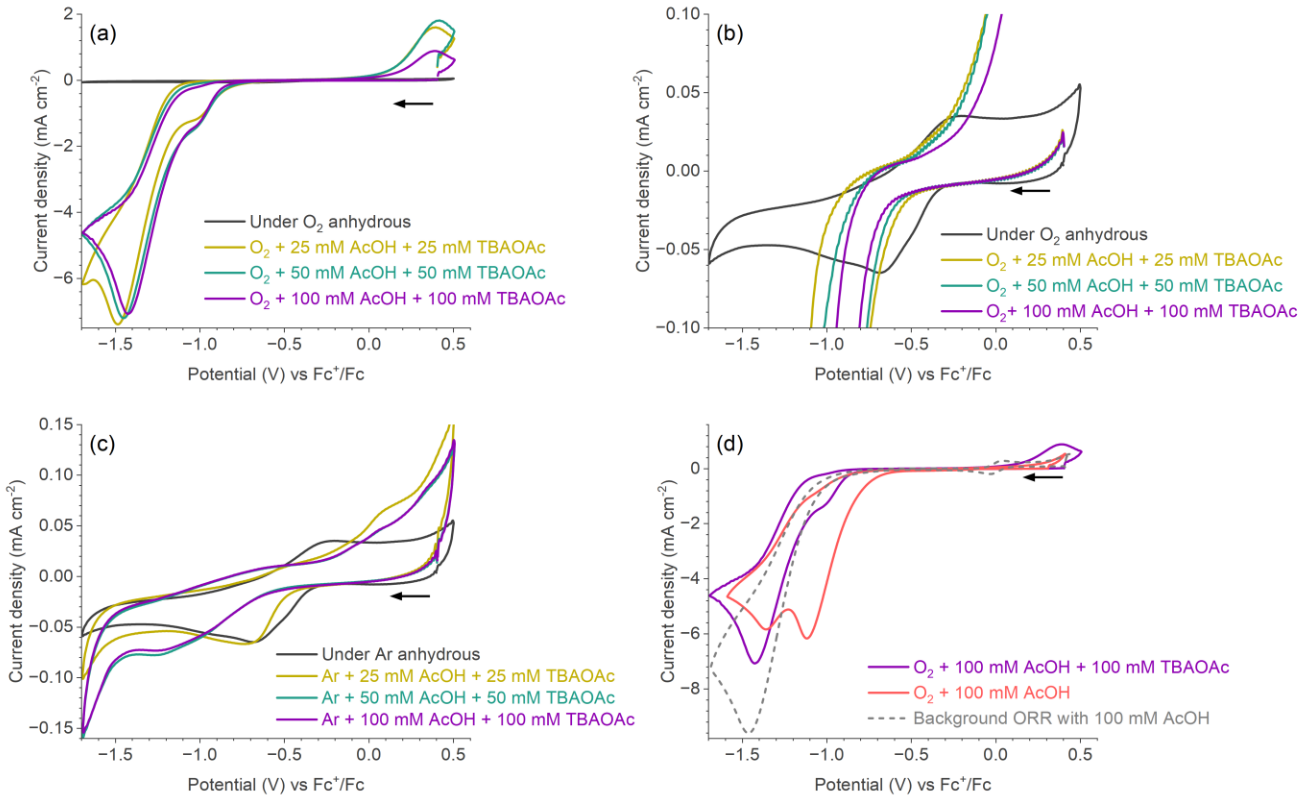

Figure S8. CV studies with Co(III) [(UMAPA)Co^{III}(Cl)]Cl complex at scan rate of 100 mV s^{-1} showing; (a) the effect of various acetic acid and acetate ions (TBAOAc) concentrations on the electrocatalytic ORR by [(UMAPA)Co^{III}(Cl)]Cl complex under O_2 saturation. (b) The zoomed plot shows the effect of various acetic acid and acetate ions (TBAOAc) concentrations on the $Co^{III/II}$ redox process under O_2 saturation. (c) effect of various amounts of acetic acid and acetate ions (TBAOAc) on $Co^{III/II}$ redox process done under argon atmosphere (d) Comparison of CV of [(UMAPA)Co^{III}(Cl)]Cl complex under O_2 saturations with 100 mM AcOH and 100 mM TBAOAc (purple trace), with only 100 mM AcOH (red trace) and background ORR current of the glassy carbon electrode without Co(III) complex. Solution conditions were 0.1 M $TBAPF_6$ dry MeCN solution with 0.5 mM [(UMAPA)Co^{III}(Cl)]Cl complex. Direction of scan was from negative to positive potentials as indicated by black arrow.

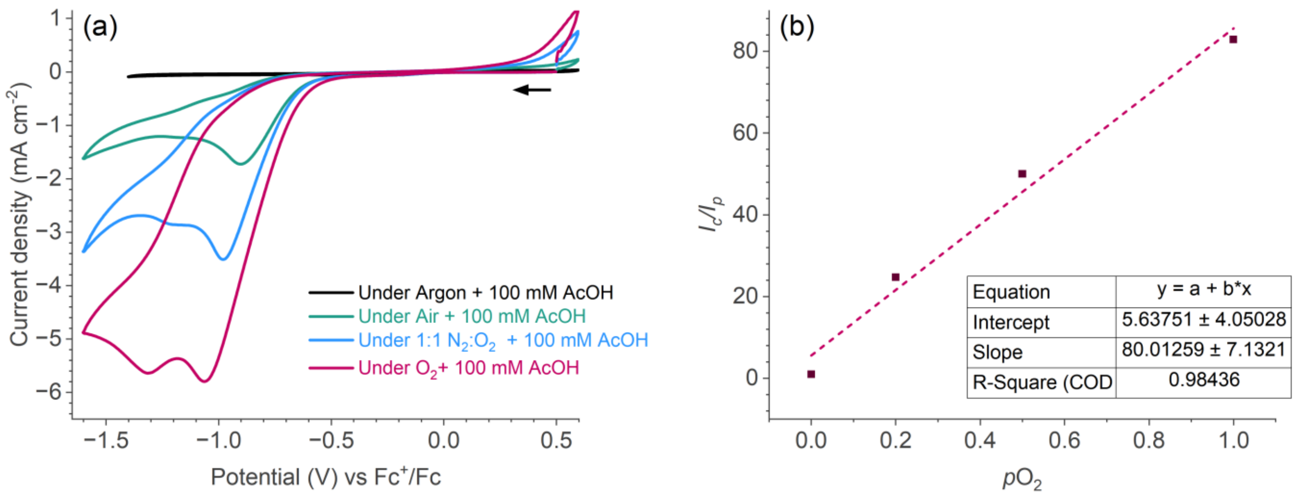

Figure S9. (a) CV studies with Co(III) [(UMAPA)Co^{III}(Cl)]Cl complex with 100 mM AcOH (200 eq.) at scan rate of 100 mV s^{-1} showing effect of O_2 concentrations. The experiments for variable O_2 concentrations were done under air (green trace), under 1:1 ratio of O_2 and N_2 (blue trace) and under pure O_2 (pink trace). All CV were recorded at 1 atm and room temperature. (b) plot showing the variation of I_c/I_p with pO_2 (partial pressure of O_2) and its linear fit (dashed line). I_c= Catalytic peak current in presence of O_2, I_p = catalyst peak current for Co(III) to Co(II) reduction wave under argon. Solution conditions were 0.1 M $TBAPF_6$ dry MeCN solution with 0.5 mM [(UMAPA)Co^{III}(Cl)]Cl complex. Direction of scan was from negative to positive potentials as indicated by black arrow.

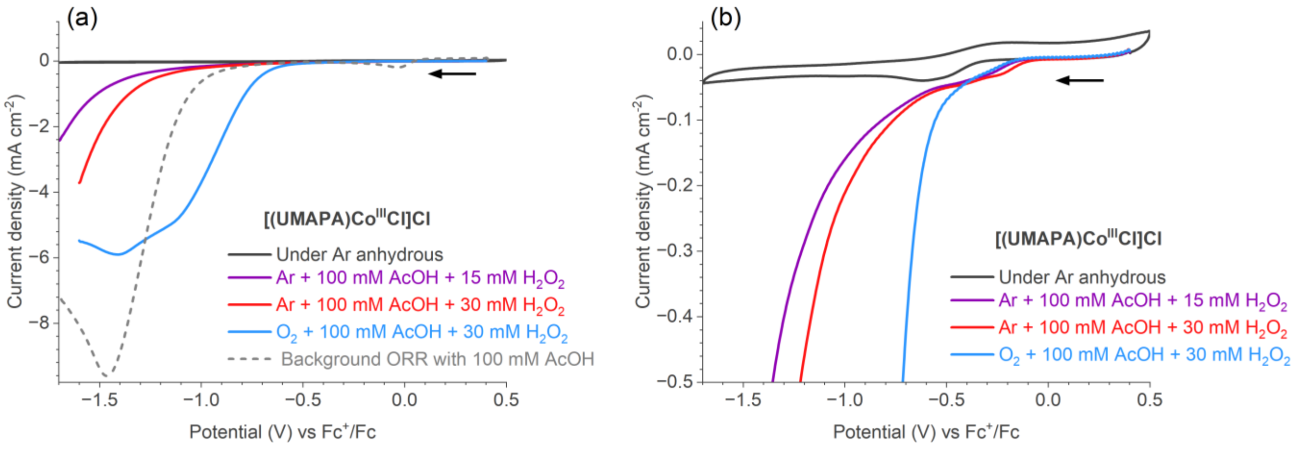

Figure S10. CV studies with Co(III) [(UMAPA)Co^{III}(Cl)]Cl complex at 100 mV s^{-1} scan rate (a) under argon and anhydrous conditions, under argon with 100 mM AcOH and 15 mM H_2O_2, under argon with 100 mM AcOH and 30 mM H_2O_2, and under O_2 saturation with 100 mM AcOH and 30 mM H_2O_2. Dashed grey trace shows the background ORR in presence of 100 mM AcOH but without the cobalt complex. Reverse scan trace is omitted for better clarity. (b) Zoomed plot showing the Co$^{III/II}$ redox process. Solution conditions were 0.1 M $TBAPF_6$ dry MeCN solution with 0.5 mM [(UMAPA)Co^{III}(Cl)]Cl complex. Direction of scan was from negative to positive potentials as indicated by black arrow.

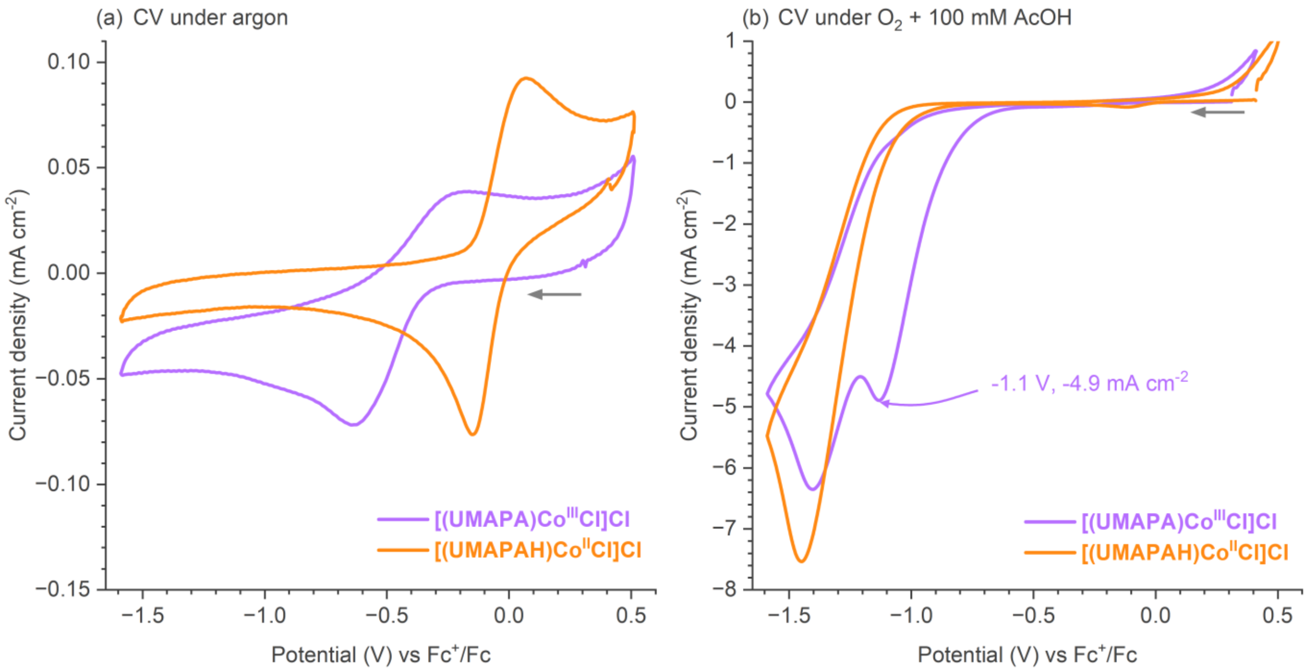

Figure S11. (a) CV of under argon with [(UMAPA)Co^{III}(Cl)]Cl and [(UMAPAH)Co^{II}(Cl)]Cl complex (0.5 mM) at 100 mV s^{-1} scan rate, under anhydrous O_2 saturation condition. (b) CV under O_2 saturation with 100 mM acetic acid (200 eq.). Solution conditions: 0.1 M $TBAPF_6$ in anhydrous MeCN. The [(UMAPAH)Co^{II}(Cl)]Cl complex did not exhibit any ORR catalytic current before the background ORR at the glassy carbon electrode.

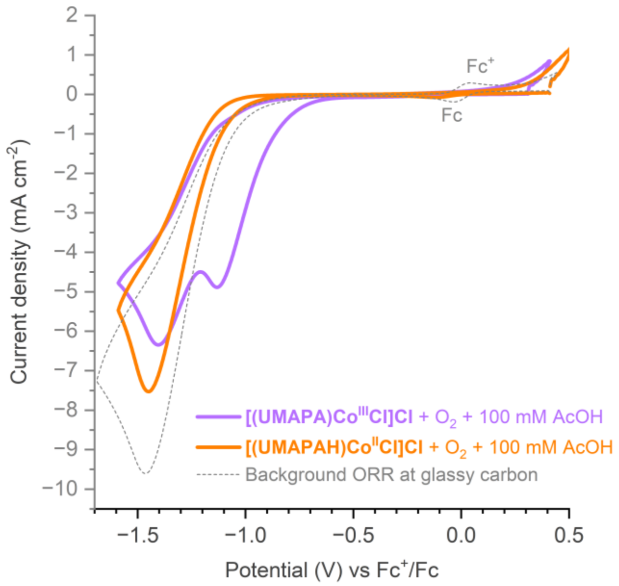

Figure S12. CV of cobalt complexes under O_2 saturation with 100 mM acetic acid (200 eq.) at 100 mV s^{-1} scan rate. The orange trace shows the CV of Co(II) [(UMAPAH)Co^{II}(Cl)]Cl complex, and the purple trace shows the CV of Co(III) [(UMAPA)Co^{III}(Cl)]Cl complex. The dotted gray trace shows the background ORR activity of glassy carbon. Solution conditions: 0.1 M $TBAPF_6$ supporting electrolyte in dry MeCN solution with 0.5 mM cobalt complexes. The blank experiment was done without any cobalt complexes.

2.4. Rotating Ring Disk Electrode Experiments

A rotating ring disk electrode (RRDE) (5 mm glassy carbon disk and platinum ring electrode) was purchased from Metrohm B.V. The Metrohm PGSTAT204 bi-potentiostat was used to record RRDE experiments. Rotating ring-disk experiments were done in 0.1 M $TBAPF_6$ dry MeCN solution with 0.5 mM [(UMAPA)Co^{III}(Cl)]Cl complex under O_2 saturation. First a LSV was recorded under O_2 at 10 mV/s scan rate without any rotation to determine window of potential to be scanned (a window of 0.1 V to -1.4 V was scanned). Then, the LSVs were recorded at 10 mV/s scan rate and different rotation speed (200 rpm to 2200 rpm) under O_2 saturation with 100 mM acetic acid added. The Platinum ring was set at + 1.0 V to oxidize any H_2O_2 produced at the glassy carbon disk electrode. The Platinum ring's theoretical collection efficiency (N_c) of 27 % was used for calculations. Eq 5.1 was used to calculate the %H_2O_2 selectivity, where p= H_2O_2 ratio, i_r= ring current, i_d= disk current, and N_c=collection efficiency in fraction. [2] Multiplying p by 100 gave %H_2O_2 selectivity. For calculation of p, at every rotation speed, the limiting current values of ring (i_r) and disk (i_d) current at potential of -1.2 V was used. The average p was then reported.

$$\boldsymbol{Eq\ 5.1} \ldots\ldots\ p = 2 \times \frac{(\frac{i_r}{N_c})}{i_d + (\frac{i_r}{N_c})}$$

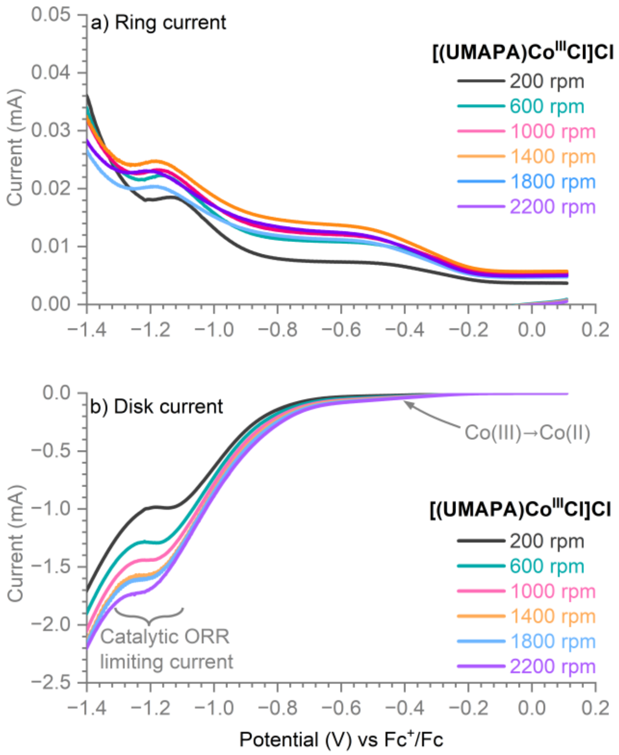

Figure S13. RRDE studies with Co(III) [(UMAPA)Co^{III}(Cl)]Cl complex. The LSVs were recorded at 10 mV s^{-1} scan rate under O_2 saturation with added 100 mM acetic acid (200 eq.). (a) Ring current at various rotation speeds, (b) Disk current at various rotation speeds. Solution conditions; 0.1 M $TBAPF_6$ dry MeCN solution with 0.5 mM [(UMAPA)Co^{III}(Cl)]Cl complex.

2.5. Electrochemical Mass Spectrometry Hyphenated (EC-ESI-MS) Experiments

The flow cell-based EC-ESI-MS setup used in this work was an update on our previous EC-ESI-MS setup, whose details can be found elsewhere. [3, 4] The key components of the flow cell-based EC-ESI-MS setup are shown in Figure S14. The potentials were controlled with Palmsens4 potentiostat (Palmsens B.V., the Netherlands). The samples were injected into an EC-ESI-MS setup with a Labm8 syringe pump control unit (Labm8, the Netherlands). The flow cell was connected to the ESI-probe of the Bruker timsTOF instrument using a fused silica capillary (16.5 cm long, OD 190 µm, ID 100 µm).

The cross-sectional view of the flow cell is shown in Figure S14c. The flow cell was manufactured by CNC machining of polypropylene blocks. The flow cell comprises two components sealed tight with a PTFE gasket (1 mm thickness), housing, the working and counter electrode assemblies, and the reference electrode (Figure S14c). The main component comprises two slots, each for the counter and working electrodes. The working electrode slot was 5 mm x 6 mm x 1.5 mm in dimension, whereas the counter electrode slot was 10 mm x 6 mm x 1.5 mm. The working and counter electrode slots were partly separated by a wall to prevent a short-circuit with the counter electrode. We used four Toray carbon paper electrodes (0.2 mm thick, Thermo Scientific Chemicals) in each slot (5 mm x 6 mm carbon paper as the working electrode and 10 mm x 6 mm as the counter electrode). The steel current collectors connected the counter electrodes to the potentiostat. A glassy carbon rod (2 mm diameter) connected the working electrodes to the potentiostat. A silver wire was used as a reference electrode and positioned between the working and counter electrodes. Current collectors, reference electrode, inlet tubing and outlet fused silica capillary were secured into the flow cell using 1/4-28 super flangeless nuts (Darwin microfluidics). The carbon paper electrodes were porous and provided higher conversion efficiency. Our cell design allowed us to easily replace the carbon paper electrodes between the experiments, preventing electrode fouling and enabling reproducibility. The inner volume of the empty assembled flow cell without the electrodes was ~ 155 µL. With all the electrodes added, the geometric inner volume of the completely assembled flow cell was ~ 65 µL. The fused silica capillary (length 16.5 cm, ID 100 µm, OD 190 µm, Postnova) transferred the species from the working electrode to the electrospray ionization-mass spectrometer (ESI-MS). The fused silica capillary was secured between the carbon paper electrodes, limiting the distance between the surface of the electrode and the capillary to the capillary wall thickness, 45 µm. Low interelectrode distance and low less volume allowed us to use low supporting electrolyte concentrations.[5]

In a typical EC-ESI-MS experiment, we loaded a O_2 saturated solution of Co(II) complex in to a syringe which was then injected into the flow cell. The flow cell then transferred the solution into the ESI probe through a fused silica capillary. Typical source conditions of Burker timsTOF were as follows: Capillary voltage= 4500 V, Nebulizer gas= 0.5 bar N_2, Dry gas= 2 mL/min, Dry gas temperature= 200 ^oC.

During **chronoamperometry EC-ESI-MS experiments**, a fixed potential was applied to the working electrode of the electrochemical flow cell for a certain time interval. The solution was simultaneously injected from the flow cell to the ESI-mass spectrometer using syringe pump. The mass spectrum was continuously recorded for the complete duration of chronoamperometry EC-ESI-MS experiment.

During **VESI-MS experiments** same EC-ESI-MS setup was used. We recorded mass spectra while performing the linear scan voltammetry (LSV) with a slow scan rate of 5 mV s^{-1}. [4] The VESI-MS method lets us detect reactive intermediates/products formed at the electrode surface in real time during a voltammetry measurement.

Data processing: From MS data of the chronoamperometry EC-ESI-MS experiments, extracted ion chromatograms (EICs) of the ions of interest were exported using Bruker data analysis software. The extracted

ions chromatograms were then normalized to the total ion current of the that particular experiment and plotted as intensity vs time plots.

From the raw MS data of the VESI-MS experiments, extracted ion chromatograms (EICs) of the ions of interest were exported using Bruker data analysis software. The extracted ions chromatograms were then normalized to the total ion current of the that particular experiment. From the raw voltammetry data of the VESI-MS experiments (acquired using Palmsens potentiostat), the time-potential-current data was extracted. The time scale of voltammetry data and normalized extracted ion chromatograms were aligned on a common time axis, then the time axis was converted to the corresponding potential axis for plotting VESI-MS plots. The time scale of the MS data of the VESI-MS experiments was corrected for the transfer time delays. The transfer time was measured during chronoamperometry EC-ESI-MS experiments by monitoring the oxidation of ferrocene to ferrocenium ion (see Figure S25).

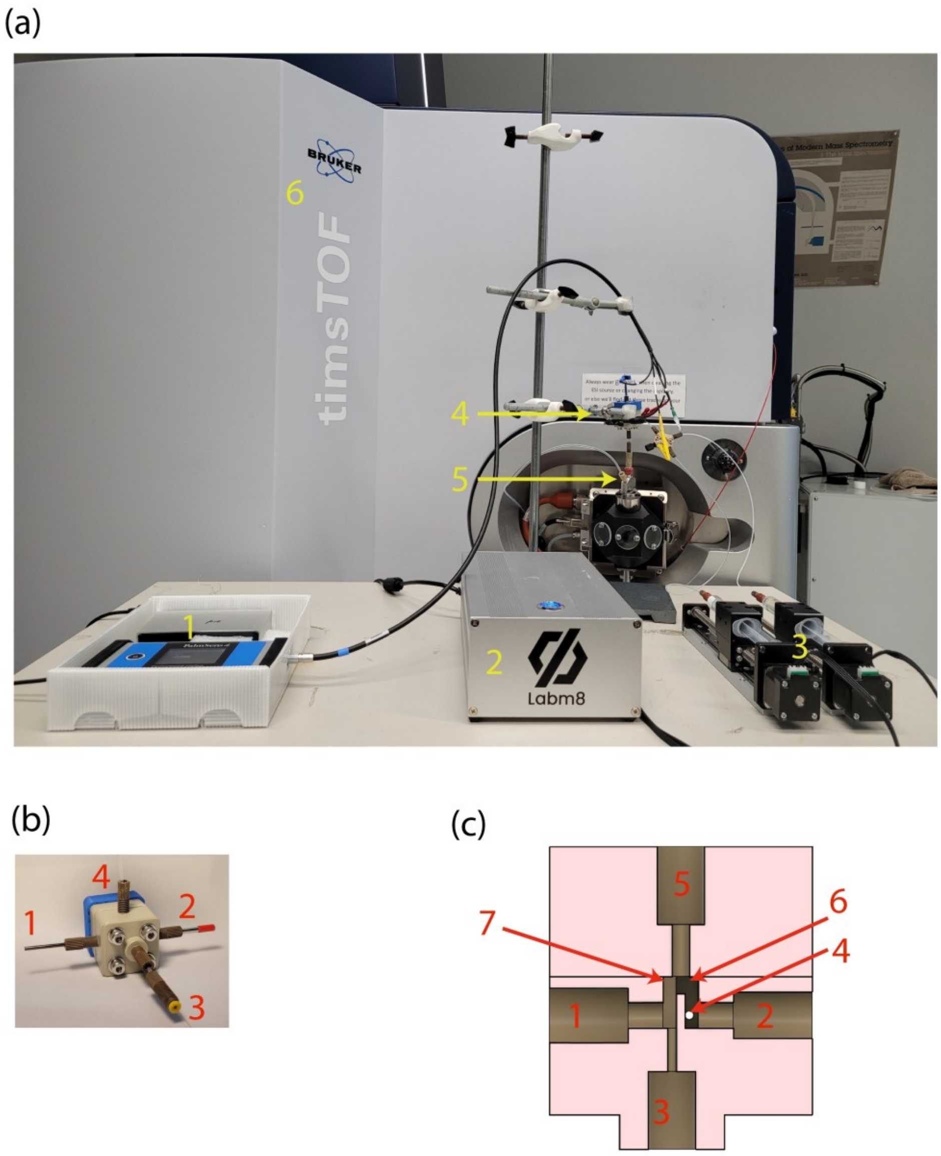

Figure S14. (a) Picture of the EC-ESI-MS instrumentation used in this work. Legends are as follows: 1= Palmsens potentiostat, 2= Labm8 syringe pump control unit, 3=syringe pump assemblies, 4= electrochemical flow cell, 5= electrospray ionization probe, 6= Bruker timsTOF; (b) picture of electrochemical flow cell. Legends are as follows: 1= working electrode connection, 2= counter electrode connection, 3= outlet showing the fused silica capillary, 4= inlet; (c) Cross sectional view of the electrochemical flow cell. Legends are as follows: 1= working electrode connection port, 2= counter electrode connection port, 3= outlet port, 4= inlet hole, 5= reference electrode connection port, 6= counter electrode slot (10 mm x 6 mm x 1.5 mm), 7= working electrode slot (5 mm x 6 mm x 1.5 mm).

Table S 1. Exact masses of ORR species detected during EC-ESI-MS studies with [(UMAPA)Co^{III}(Cl)]Cl complex.

Species	Molecular Formula	Found Exact Mass[a]	Calculated Exact Mass
**[(UMAPA)Co^{III}]$^{2+}$**	$C_{27}H_{21}CoF_6N_6O$	309.053	309.050
**[(UMAPAH)Co^{II}]$^{2+}$**	$C_{27}H_{22}CoF_6N_6O$	309.553	309.554
**[(UMAPAH)Co^{III}(O$_2$H)]$^{2+}$**	$C_{27}H_{23}CoF_6N_6O_3$	326.052	326.053
**[(UMAPAH)Co^{III}(Cl)]$^{2+}$**	$C_{27}H_{22}ClCoF_6N_6O$	327.038	327.038
**[(UMAPA)Co^{II}]$^{+}$**	$C_{27}H_{21}CoF_6N_6O$	618.099	618.101
**[(UMAPA^{OH})Co^{II}]$^{+}$**	$C_{27}H_{21}CoF_6N_6O_2$	634.095	634.096
**[(UMAPA)Co^{III}(OH)]$^{+}$**	$C_{27}H_{22}CoF_6N_6O_2$	635.102	635.104
**[(UMAPA)Co^{III}(CN)]$^{+}$**	$C_{28}H_{21}CoF_6N_7O$	644.102	644.104
**[(UMAPA)Co^{III}(O$_2$H)]$^{+}$**	$C_{27}H_{22}CoF_6N_6O_3$	651.098	651.099
**[(UMAPA)Co^{III}(Cl)]$^{+}$**	$C_{27}H_{21}ClCoF_6N_6O$	653.070	653.070
**[(UMAPAH)Co^{II}(Cl)]$^{+}$**	$C_{27}H_{22}ClCoF_6N_6O$	654.076	654.078
**[(UMAPA^{OH})Co^{III}(Cl)]$^{+}$**	$C_{27}H_{21}ClCoF_6N_6O_2$	669.065	669.065
**[(UMAPAH^{OH})Co^{II}(Cl)]$^{+}$**	$C_{27}H_{22}ClCoF_6N_6O_2$	670.072	670.073
**[(UMAPA)Co^{II}(Cl)(Na)]$^{+}$**	$C_{27}H_{21}ClCoF_6N_6NaO$	676.054	676.060
**[(UMAPA)Co^{III}(AcO)]$^{+}$**	$C_{29}H_{24}CoF_6N_6O_3$	677.114	677.115
**[(UMAPAH)Co^{III}(AcO)(OH)]$^{+}$**	$C_{29}H_{26}CoF_6N_6O_4$	695.124	695.125
**[(UMAPA)Co^{II}(AcO)(Na)]$^{+}$**	$C_{29}H_{24}CoF_6N_6NaO_3$	700.099	700.104
**[(UMAPAH^{OH})Co^{III}(AcO)(OH)]$^{+}$**	$C_{29}H_{26}CoF_6N_6O_5$	711.120	711.120
**[(UMAPACo)$_2$(Cl)]$^{+}$**	$C_{54}H_{42}ClCo_2F_{12}N_{12}O_2$	1271.171	1271.171
**[(UMAPACo)$_2$(O)(Cl)]$^{+}$**	$C_{54}H_{42}ClCo_2F_{12}N_{12}O_3$	1287.170	1287.166
**[(UMAPACo)$_2$(O)$_2$(Cl)]H^{+}**	$C_{54}H_{43}ClCo_2F_{12}N_{12}O_4$	1304.171	1304.169
**[(UMAPACo)$_2$(Cl)$_2$]H^{+}**	$C_{54}H_{43}Cl_2Co_2F_{12}N_{12}O_2$	1307.147	1307.148

[a]The measurements were done on different days so the error in exact masses varies with the error in the calibration.

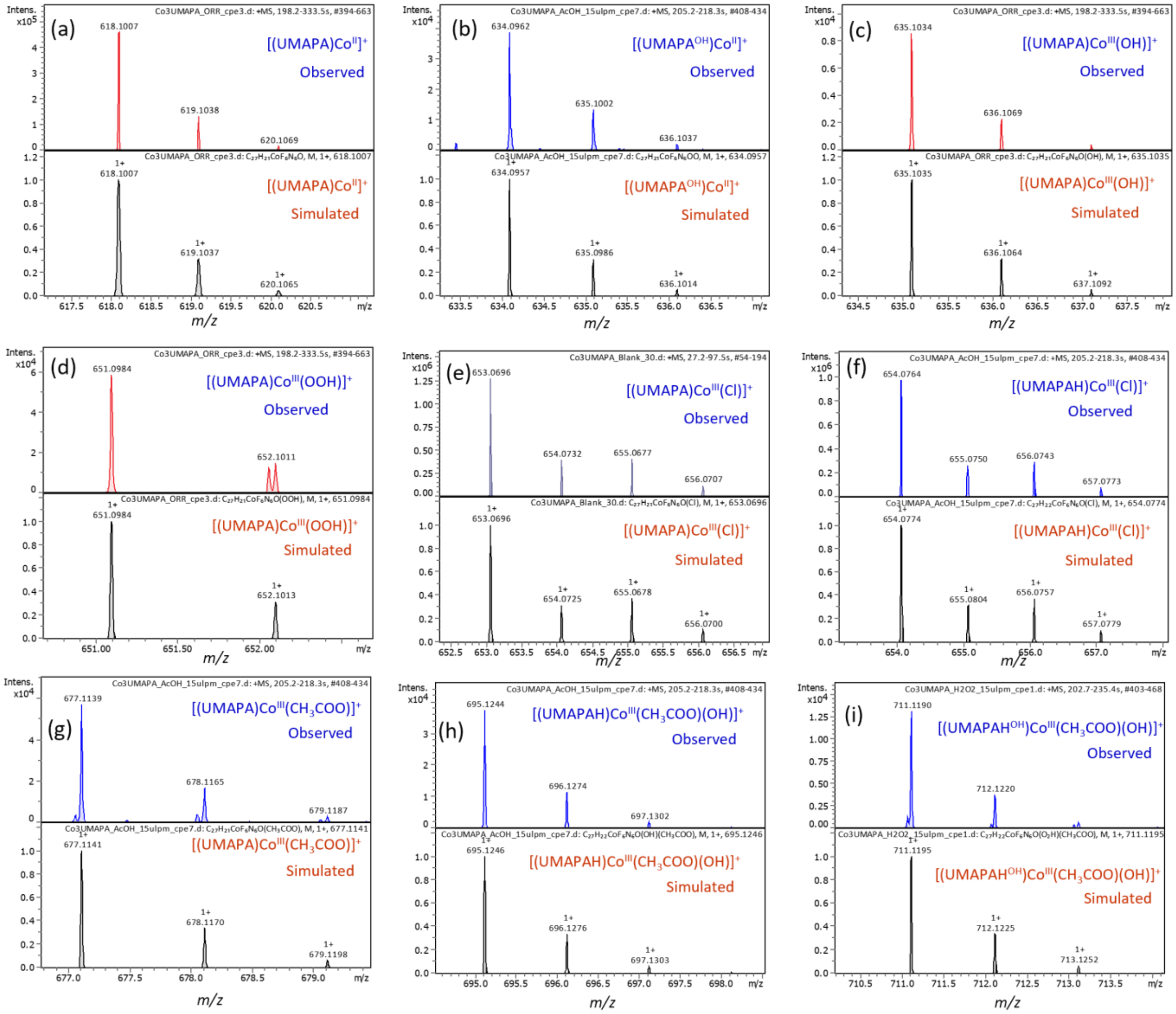

Figure S15. Comparison of observed and simulated mass spectrum of EC-ESI-MS detected ORR intermediates. (a) $[(UMAPA)Co^{II}]^+$ (*m/z* 618.10), (b) $[(UMAPA^{OH})Co^{II}]^+$ (*m/z* 634.10), (c) $[(UMAPA)Co^{III}(OH)]^+$ (*m/z* 635.10), (d) $[(UMAPA)Co^{III}(O_2H)]^+$ (*m/z* 651.10), (e) $[(UMAPA)Co^{III}(Cl)]^+$ (*m/z* 653.07), (f) $[(UMAPAH)Co^{II}(Cl)]^+$ (*m/z* 654.08), (g) $[(UMAPA)Co^{III}(AcO)]^+$ (*m/z* 677.11), (h) $[(UMAPAH)Co^{III}(AcO)(OH)]^+$ (*m/z* 695.12), (i) $[(UMAPAH^{OH})Co^{III}(AcO)(OH)]^+$ (*m/z* 711.12). The ions were generated from an O_2 saturated dry MeCN solution of 0.05 mM $[(UMAPA)Co^{III}(Cl)]Cl$ complex at a flow rate of 15 µL min^{-1}.

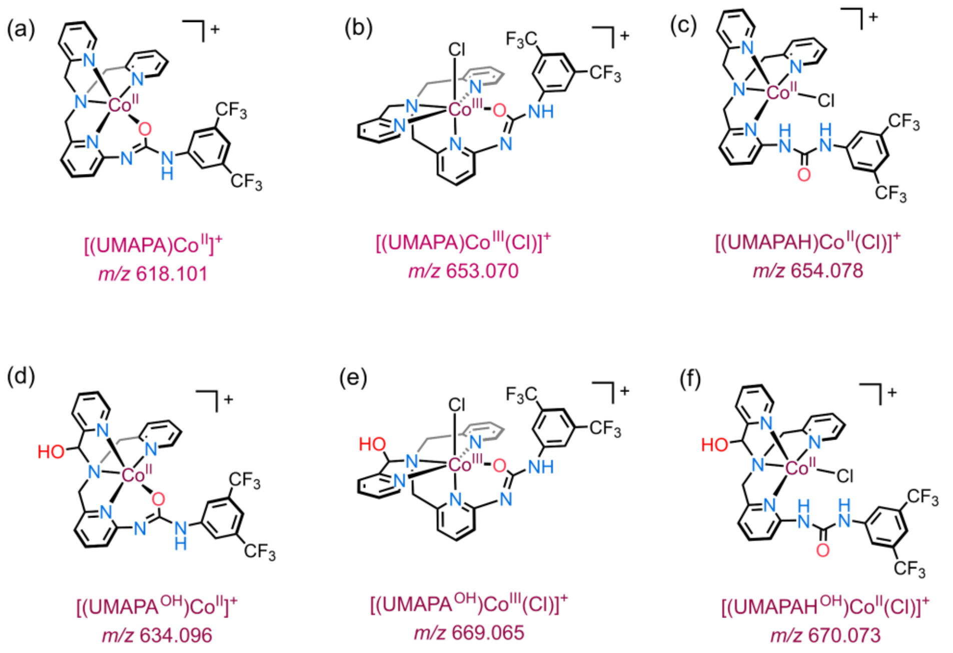

Figure S16. (a)-(c) Proposed structures of EC-ESI-MS detected cobalt complexes without ligand hydroxylation; (d)-(f) Proposed structures of EC-ESI-MS detected cobalt complexes with hydroxylated ligand.

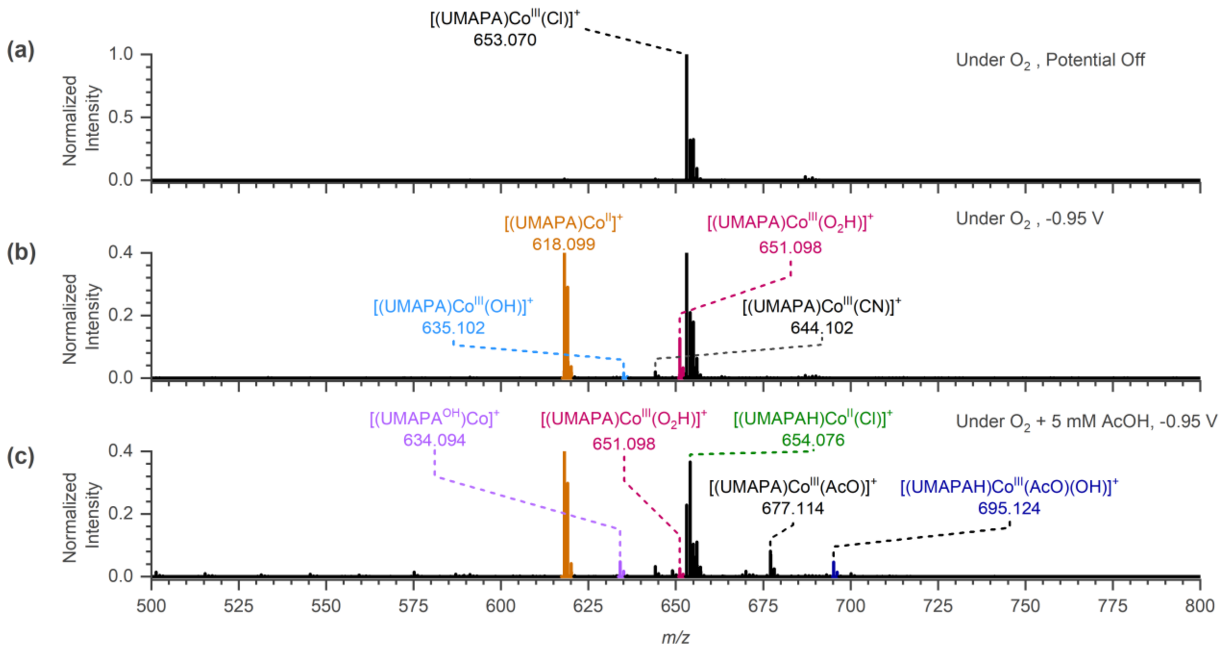

Figure S17. EC-ESI-MS studies with cobalt complex [(UMAPA)Co^{III}(Cl)]Cl under O_2. (a) zoomed EC-ESI-MS spectrum under anhydrous O_2 saturation conditions, without any applied potential at the working electrode, (b) zoomed EC-ESI-MS spectrum under O_2 at -0.95 V vs Fc^+/Fc, (c) zoomed EC-ESI-MS spectrum under O_2 at -0.95 V vs Fc^+/Fc with 5 mM AcOH (100 eq.). The ions were generated from an O_2 saturated dry MeCN solution of 0.05 mM [(UMAPA)Co^{III}(Cl)]Cl complex at a flow rate of 15 µL min^{-1}.

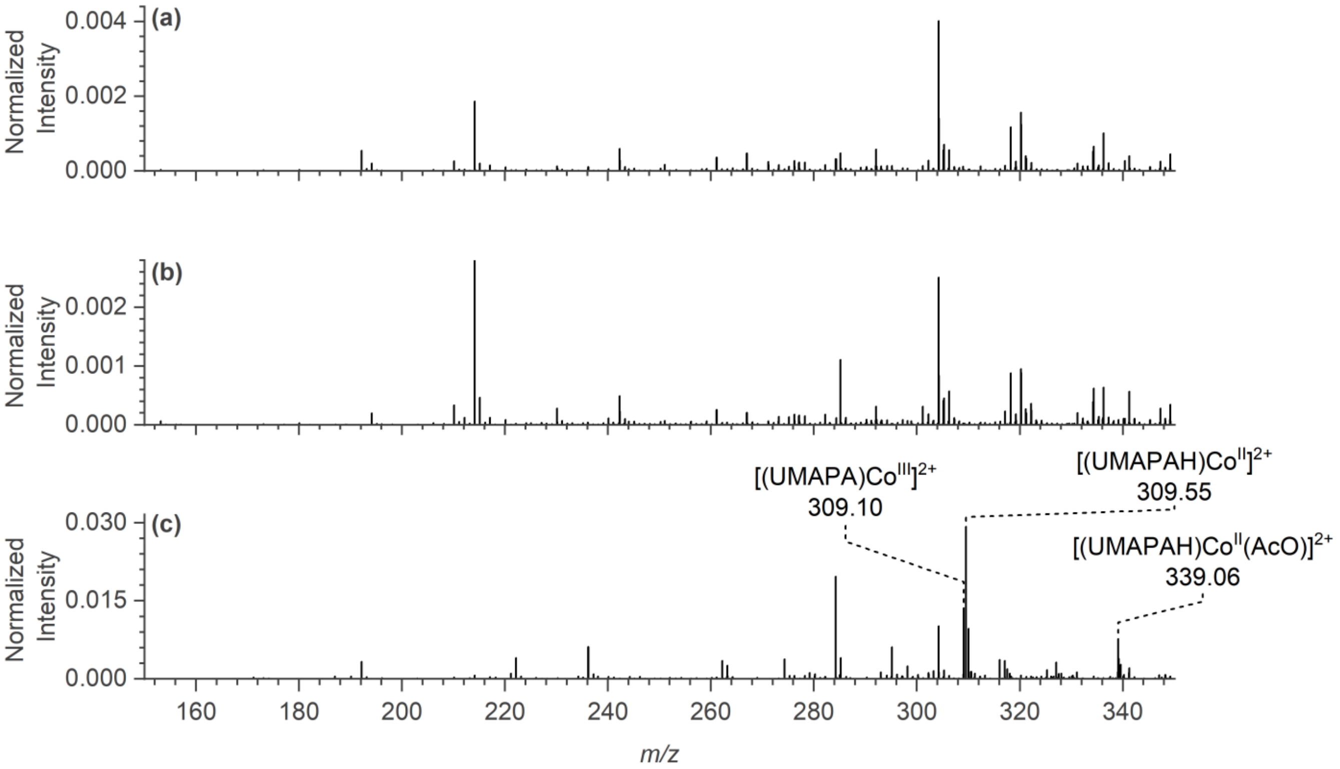

Figure S18. EC-ESI-MS studies with cobalt complex [(UMAPA)Co^{III}(Cl)]Cl under O_2 showing lower mass range for dications. (a) EC-ESI-MS spectrum under O_2, without any applied potential at the working electrode, (b) EC-ESI-MS spectrum under O_2 at -0.95 V vs Fc^+/Fc, (c) EC-ESI-MS spectrum under O_2 at -0.95 V vs Fc^+/Fc with 5 mM AcOH (100 eq.). The ions were generated from an O_2 saturated dry MeCN solution of 0.05 mM [(UMAPA)Co^{III}(Cl)]Cl complex at a flow rate of 15 µL min^{-1}.

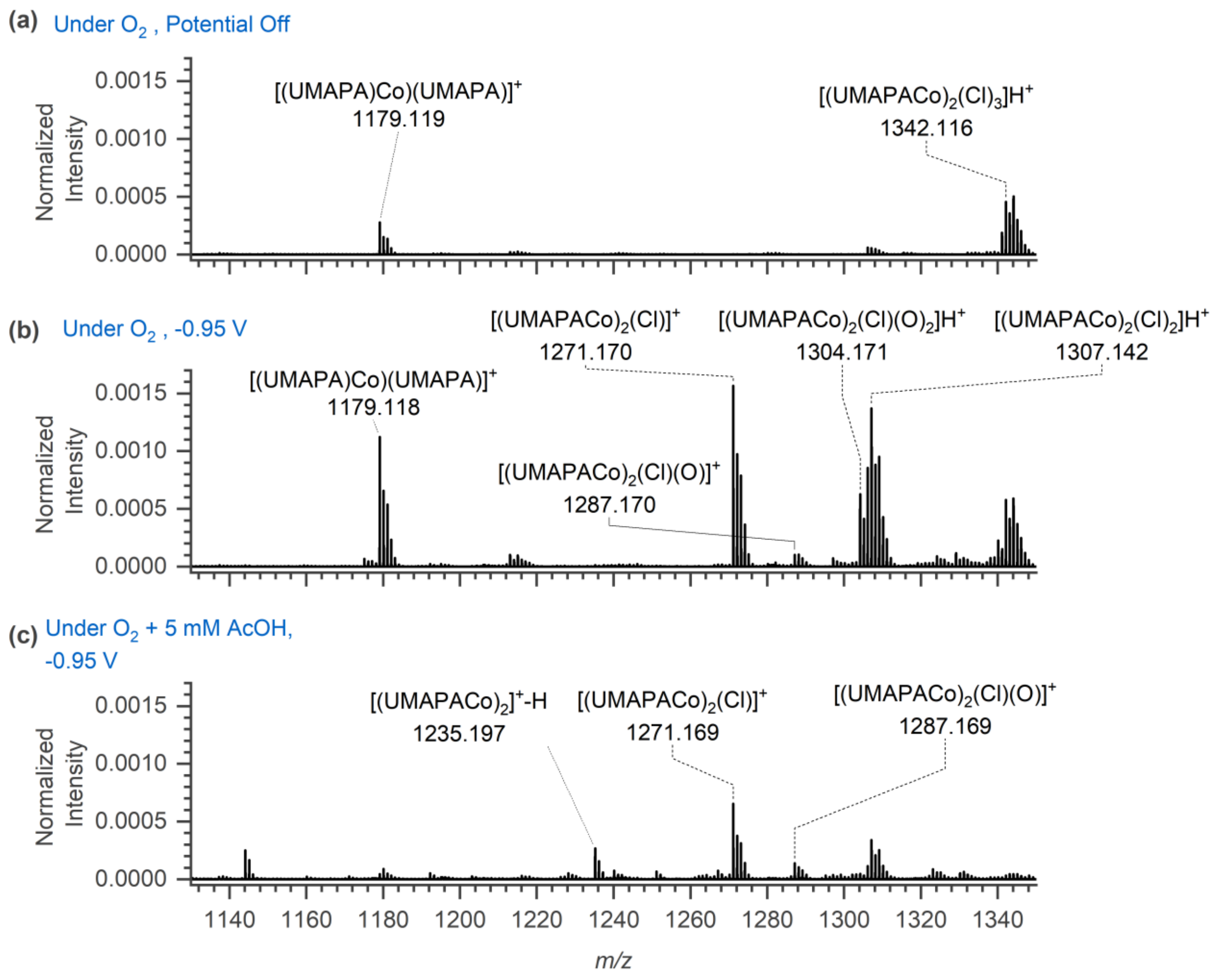

Figure S19. EC-ESI-MS studies with cobalt complex [(UMAPA)Co^{III}(Cl)]Cl under O_2 showing higher mass range for dimeric ions. (a) EC-ESI-MS spectrum under O_2, without any applied potential at the working electrode, (b) EC-ESI-MS spectrum under O_2 at -0.95 V vs Fc^+/Fc, (c) EC-ESI-MS spectrum under O_2 at -0.95 V vs Fc^+/Fc with 5 mM AcOH (100 eq.). The ions were generated from an O_2 saturated dry MeCN solution of 0.05 mM [(UMAPA)Co^{III}(Cl)]Cl complex at a flow rate of 15 µL min^{-1}.

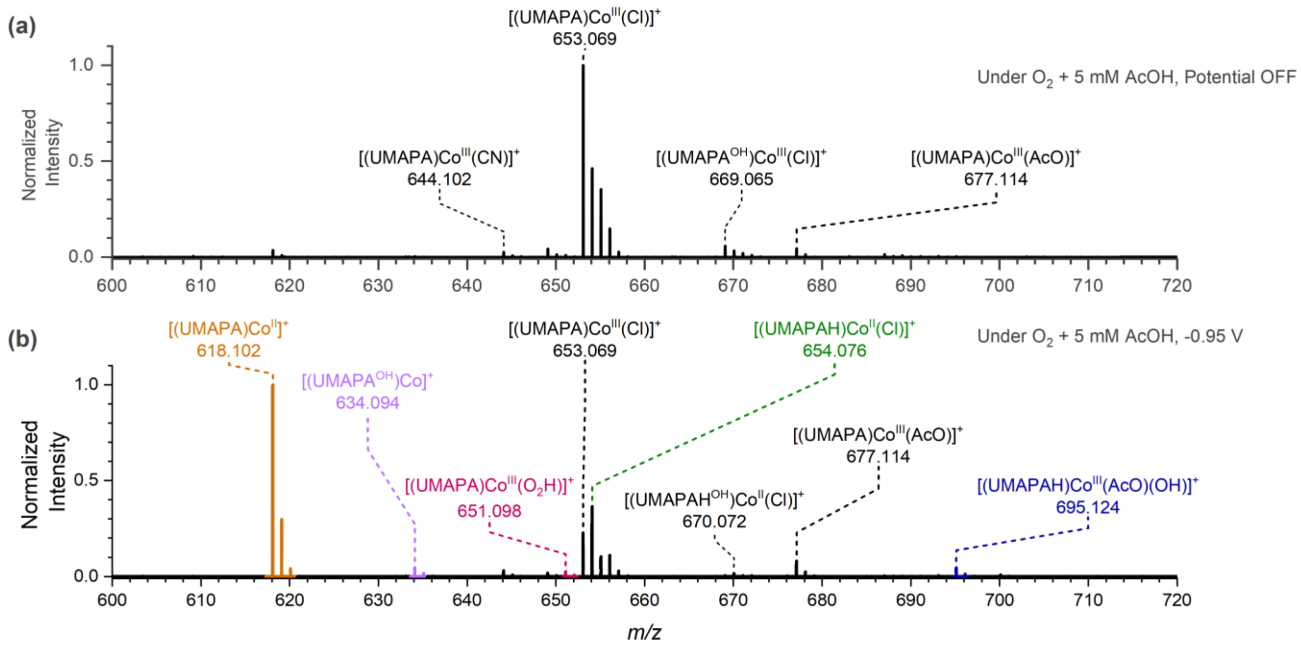

Figure S20. EC-ESI-MS studies with cobalt complex [(UMAPA)Co^{III}(Cl)]Cl under O_2 with 5 mM AcOH. (a) EC-ESI-MS spectrum under O_2, without any applied potential at the working electrode, (b) EC-ESI-MS spectrum under O_2 at -0.95 V vs Fc^+/Fc. The ions were generated from an O_2 saturated dry MeCN solution of 0.05 mM [(UMAPA)Co^{III}(Cl)]Cl complex with 5 mM AcOH at a flow rate of 15 µL min^{-1}.

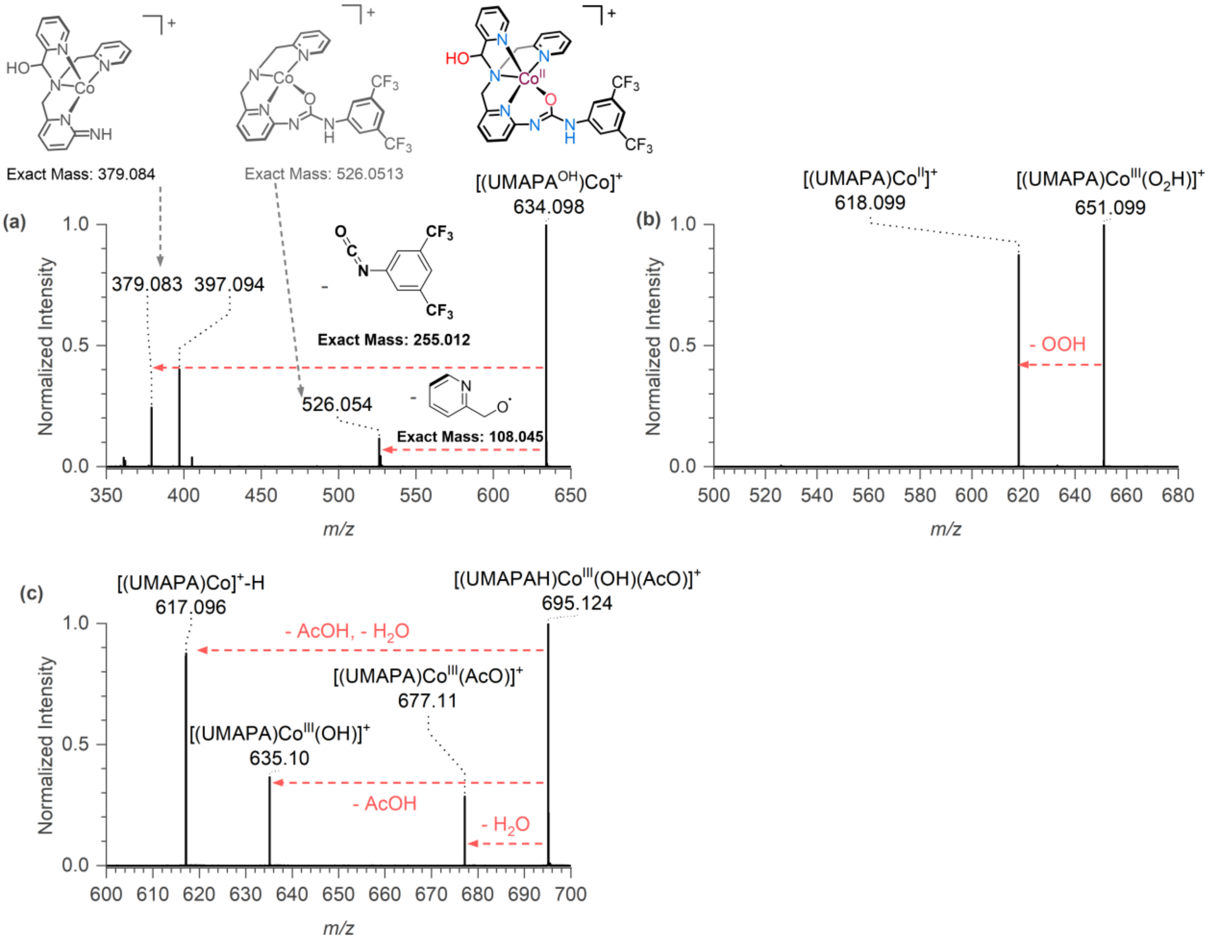

Figure S21. Collision-induced dissociation (CID) spectra of ORR intermediates. All the ions were generated from an O_2 saturated dry MeCN solution of 0.05 mM [(UMAPA)Co^{III}(Cl)]Cl complex with 5 mM AcOH (100 eq.) at a flow rate of 15 µL min^{-1} (a) CID spectrum of [(UMAPA^{OH})Co^{II}]$^+$ (*m/z* 634.09) at collision energy of 30 eV (E_{lab}), this ion lost a neutral fragment of mass 108.044 which corresponds to the (pyridin-2-yl)methoxyl radical. This suggested that the UMAPA ligand was hydroxylated at pyridyl position. For comparison, possible structures of the fragments are also presented with their exact masses. (b) CID spectrum of peroxo intermediate [(UMAPA)Co^{III}(O_2H)]$^+$ (*m/z* 651.10) at collision energy of 9 eV (E_{lab}), (c) CID spectrum of [(UMAPAH)Co^{III}(OH)(AcO)]$^+$ (*m/z* 695.12) at collision energy of 16 eV (E_{lab}).

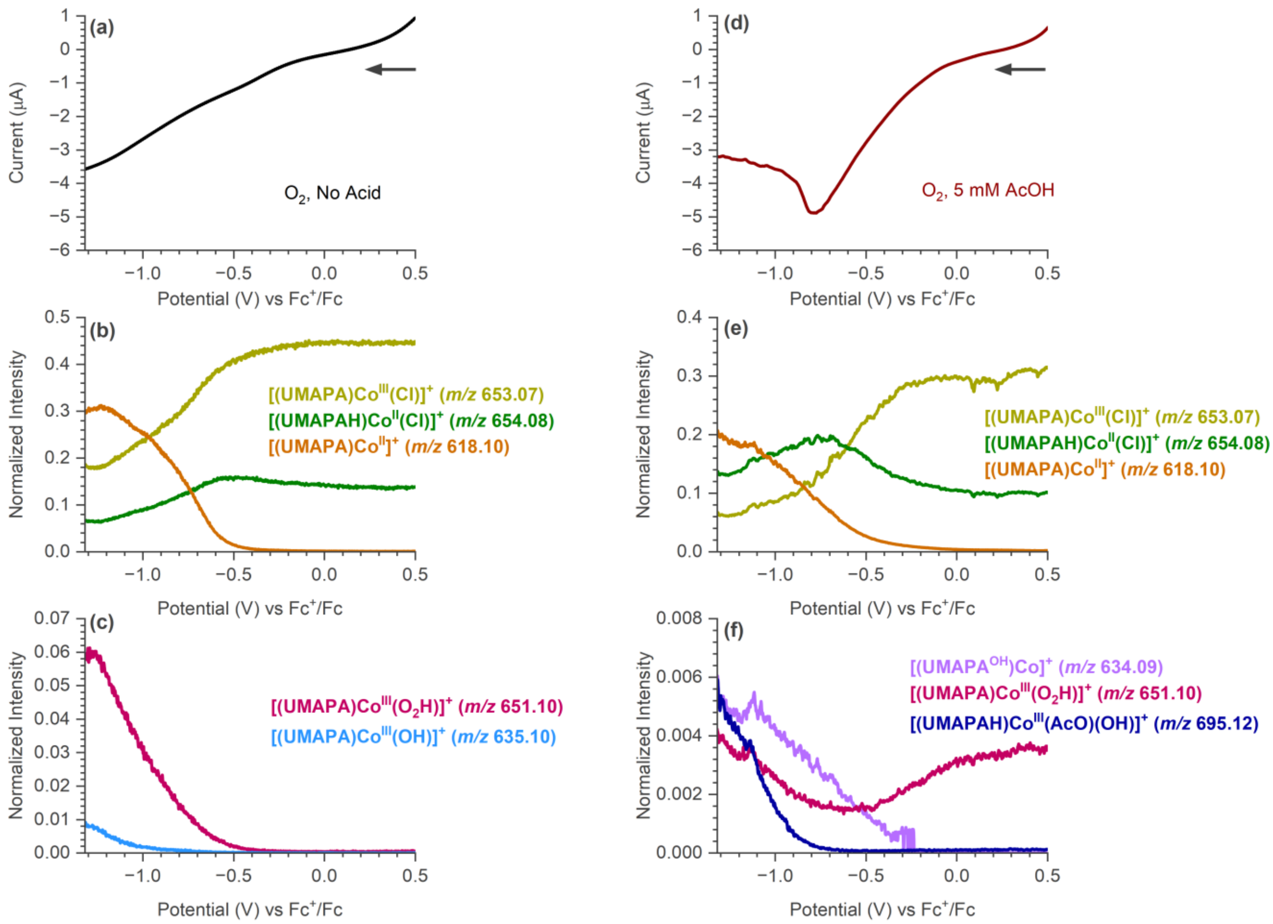

Figure S22. VESI-MS (Voltammetry - Electrospray Ionization Mass Spectrometry) experiments with cobalt complex $[(UMAPA)Co^{III}(Cl)]Cl$ under O_2 saturation. (a) LSV was recorded at 5 mV s^{-1} during the VESI-MS experiment under O_2 without any external proton source from a solution of 0.05 mM $[(UMAPA)Co^{III}(Cl)]Cl$ complex in MeCN at a flow rate of 15 µL min^{-1}. The arrow indicates the direction of scan. (d) LSV was recorded at 5 mV s^{-1} during the VESI-MS experiment under O_2 with 5 mM AcOH (100 eqs.). Plots (b) to (c) and (e) to (f) show the corresponding extracted ion chromatograms of relevant ORR intermediates generated during the VESI-MS experiment.

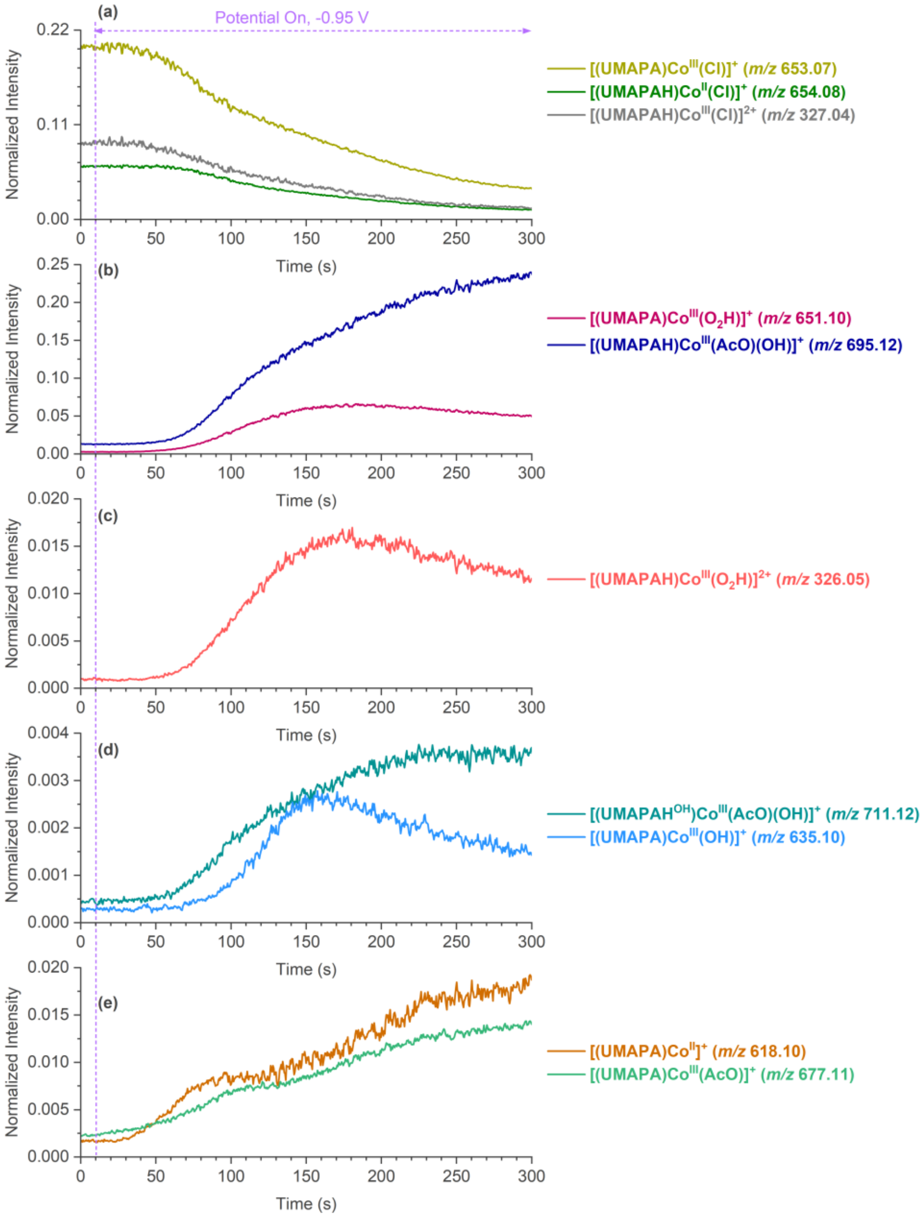

Figure S23. Chronoamperometry EC-ESI-MS experiments at -0.95 V vs Fc^+/Fc showing the evolution of ORR intermediates over time. All the ions were generated from a solution of 0.05 mM [(UMAPA)Co^{III}(Cl)]Cl complex in MeCN with H_2O_2 (15 mM) and 10 mM AcOH (200 eq.) at a flow rate of 15 µL min^{-1}. The potential was started at 10 s. Extracted ion chromatograms (EICs) vs time plots of (a) [(UMAPAH)Co^{III}(Cl)]$^{2+}$ (*m/z* 327.04), [(UMAPA)Co^{III}(Cl)]$^+$ (*m/z* 653.07), [(UMAPAH)Co^{II}(Cl)]$^+$ (*m/z* 654.08); (b) [(UMAPA)Co^{III}(O_2H)]$^+$ (*m/z* 651.10), [(UMAPAH)Co^{III}(AcO)(OH)]$^+$ (*m/z* 695.12); (c) [(UMAPAH)Co^{III}(O_2H)]$^{2+}$ (*m/z* 326.05) (d) [(UMAPA)Co^{III}(OH)]$^+$ (*m/z* 635.10), [(UMAPAH^{OH})Co^{III}(AcO)(OH)]$^+$ (*m/z* 711.12) and (e) [(UMAPA)Co^{II}]$^+$ (*m/z* 618.10), [(UMAPA)Co^{III}(AcO)]$^+$ (*m/z* 677.11).

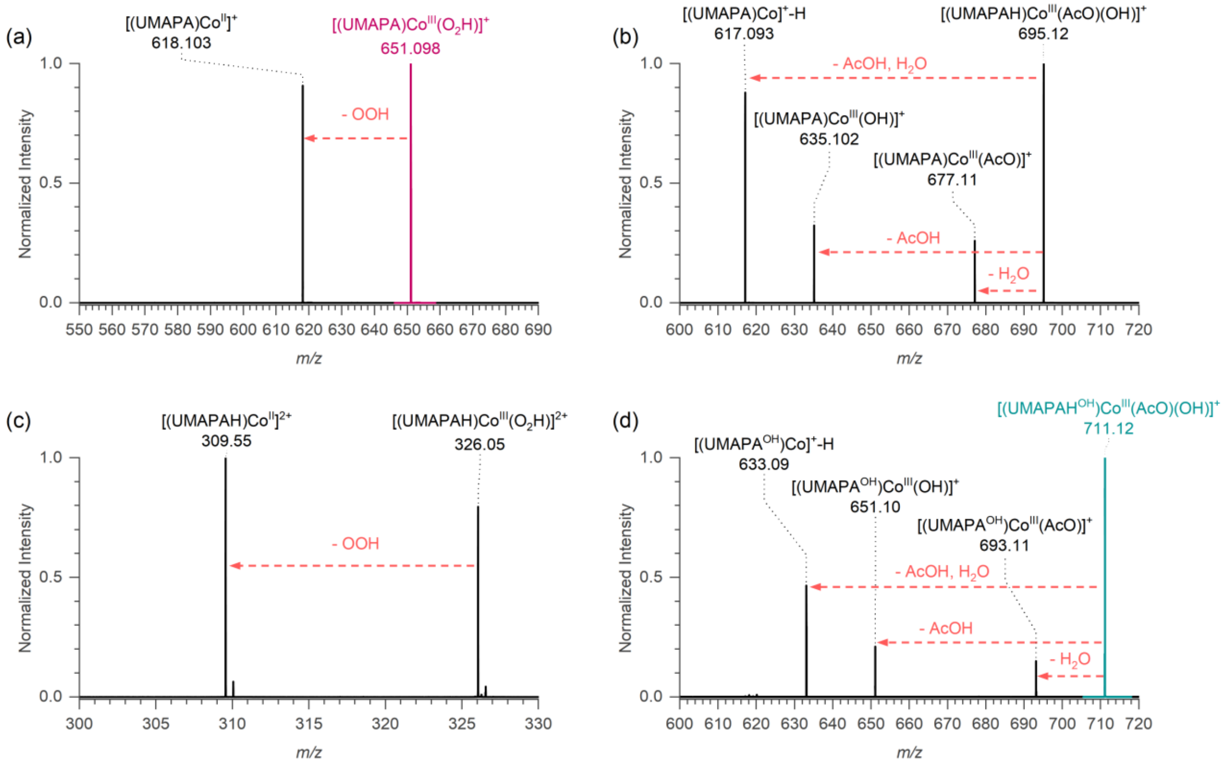

Figure S24. Collision-induced dissociation (CID) spectra of ORR intermediates. All the ions were generated from a solution of 0.05 mM [(UMAPA)Co^{III}(Cl)]Cl complex in MeCN with H_2O_2 (15 mM) and 10 mM AcOH (100 eq.) at a flow rate of 15 µL min^{-1}. (a) CID spectrum of peroxo intermediate [(UMAPA)Co^{III}(O$_2$H)]$^+$ (*m/z* 651.10) at collision energy of 9 (E_{lab}), (c) CID spectrum of [(UMAPAH)Co^{III}(AcO)(OH)]$^+$ (*m/z* 695.12) at collision energy of 16 eV (E_{lab}), (c) CID spectrum of [(UMAPAH)Co^{III}(O$_2$H)]$^{2+}$ (*m/z* 326.05) at collision energy of 2 eV (E_{lab}), (d) CID spectrum of [(UMAPAH^{OH})Co^{III}(OH)(AcO)]$^+$ (*m/z* 711.12) at collision energy of 16 eV (E_{lab}).

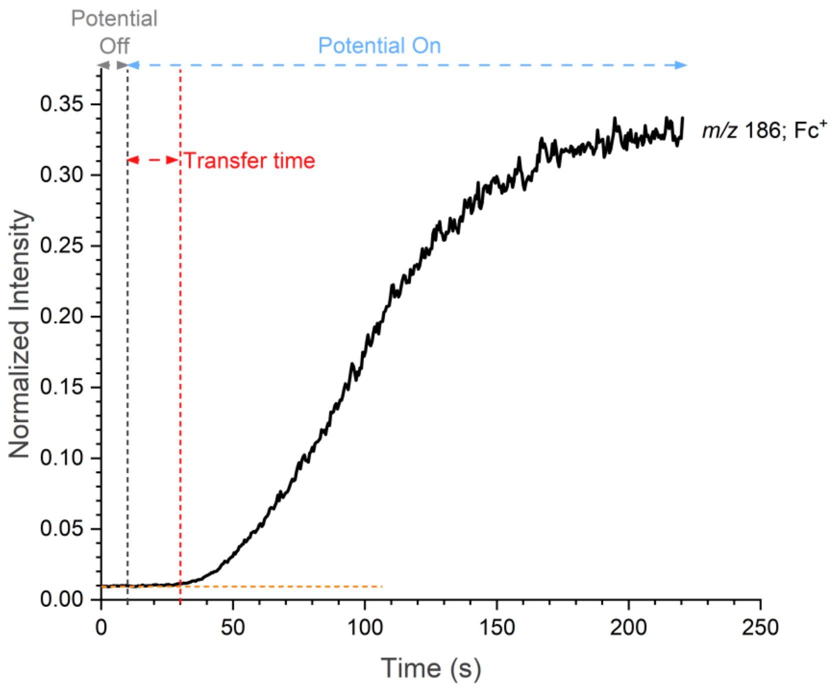

Figure S25. Extracted ion chromatogram (EIC) of ferrocenium ion (*m/z* 186.012) recorded to measure the transfer time of ions generated during EC-ESI-MS experiments to ESI source. The ions were generated during EC-ESI-MS experiments at 1 V, from the dry MeCN solution of 0.05 mM ferrocene. The solution was pumped from the EC-ESI-MS cell to the ESI source of the TIMSTOF mass spectrometer at flow rate of 15 µL min^{-1}. The average transfer time was 20 s with the fused silica capillary length of 16.5 cm.

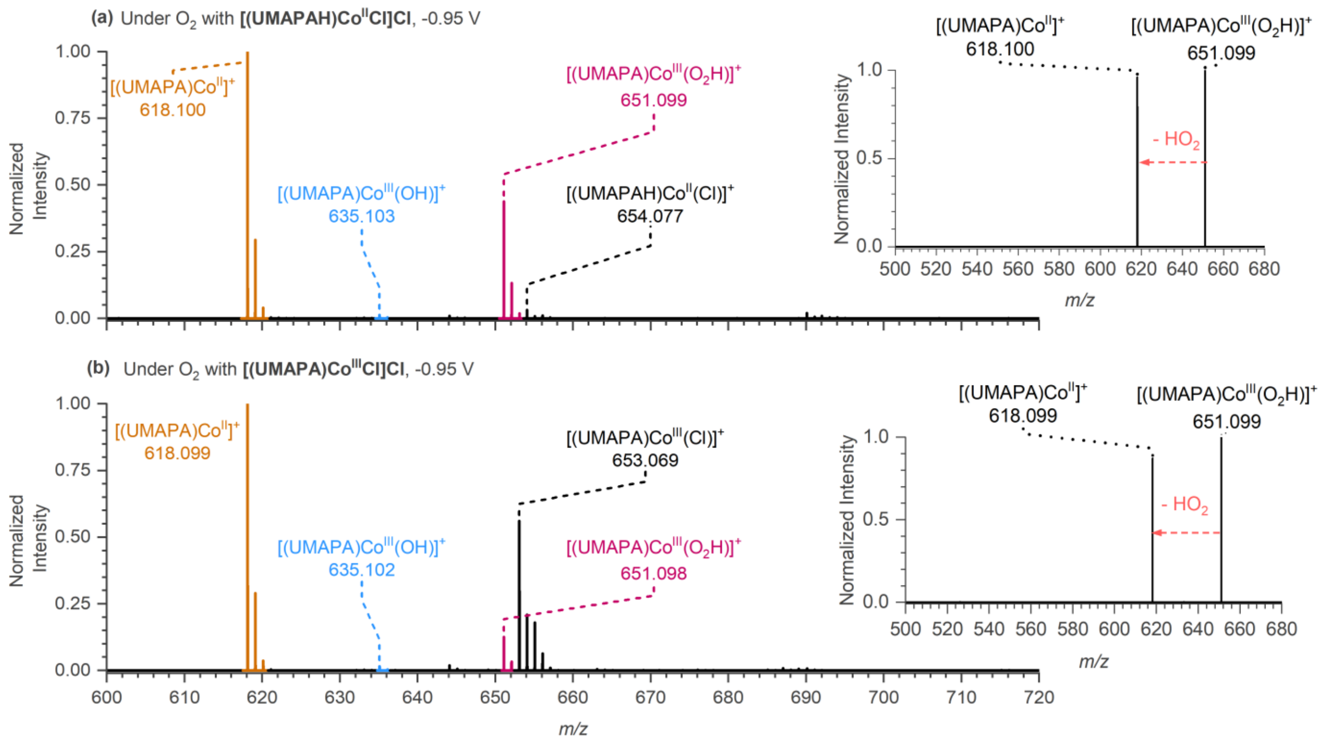

Figure S26. EC-ESI-MS studies with cobalt complex [(UMAPAH)Co^{II}(Cl)]Cl and [(UMAPA)Co^{III}(Cl)]Cl under O_2 saturation at -0.95 V vs Fc$^+$/Fc. (a) EC-ESI-MS spectrum of Co(II) complex [(UMAPAH)Co^{II}(Cl)]Cl, inset shows the CID spectrum of peroxo intermediate [(UMAPA)Co^{III}(OOH)]$^+$ (*m/z* 651.10) at a collision energy of 9 eV (E_{lab}), (b) EC-ESI-MS spectrum of Co(III) complex [(UMAPA)Co^{III}(Cl)]Cl, inset shows the CID spectrum of peroxo intermediate [(UMAPA)Co^{III}(OOH)]$^+$ (*m/z* 651.10) at a collision energy of 9 eV (E_{lab}). The ions were generated from the dry MeCN solution of 0.05 mM respective cobalt complex without any external proton source at a flow rate of 15 µL min^{-1}.

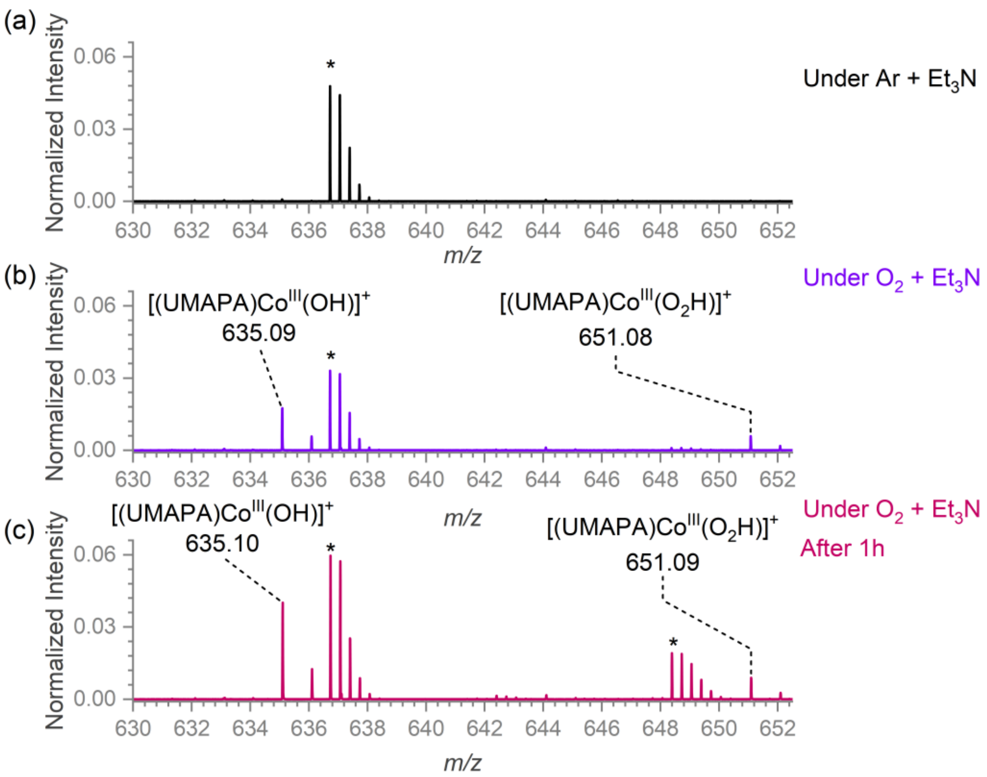

Figure S27. Zoomed ESI-MS spectra of the acetonitrile solution of [(UMAPAH)Co^{II}(Cl)]Cl at 298 K after adding 1.5 equivalent of triethyl amine (a) under argon and (b) O_2 (c) Under O_2 after 1 h. The zoomed spectra show the increase in the abundance of Co(III) hydroxo [(UMAPA)Co^{III}(OH)]$^+$ (*m/z* 635.10) and Co(III) hydroperoxo complex [(UMAPA)Co^{III}(OOH)]$^+$ (*m/z* 651.10). The multiply charged peak marked with (*) could be the impurity, possibly coming from the clusters of triethyl ammonium salts formed in sample during the experiments. These peaks were absent from the spectra in absence of triethyl amine (see Figure S28). However, we could not exactly assign these impurity peaks solely based on their *m/z* values.

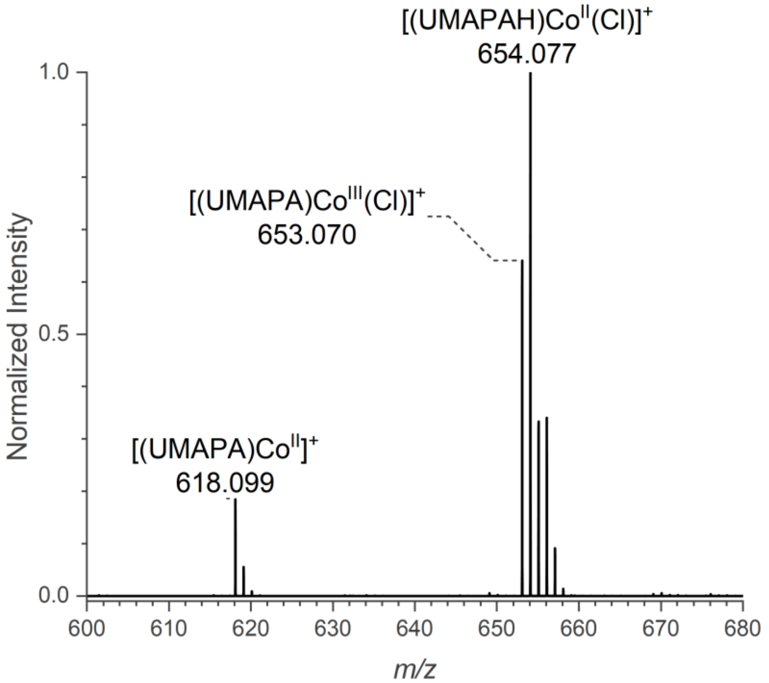

Figure S28. The mass spectrum of the solution of [(UMAPAH)Co^{II}(Cl)]Cl complex in the acetonitrile that was left under air for 2 days. The spectrum shows the oxidation of the original Co(II) complex [(UMAPAH)Co^{II}(Cl)]$^{+}$ (*m/z* 654.08) to the Co(III) complex [(UMAPA)Co^{III}(C)]$^{+}$ (*m/z* 653.07) over time by the atmosphere oxygen. The aerobic oxidation of the Co(II) [(UMAPAH)Co^{II}(Cl)]Cl complex was slow as we only observed it after 2 days. The freshly prepared solution of the Co(II) [(UMAPAH)Co^{II}(Cl)]Cl complex did not possess the oxidized Co(III) species.

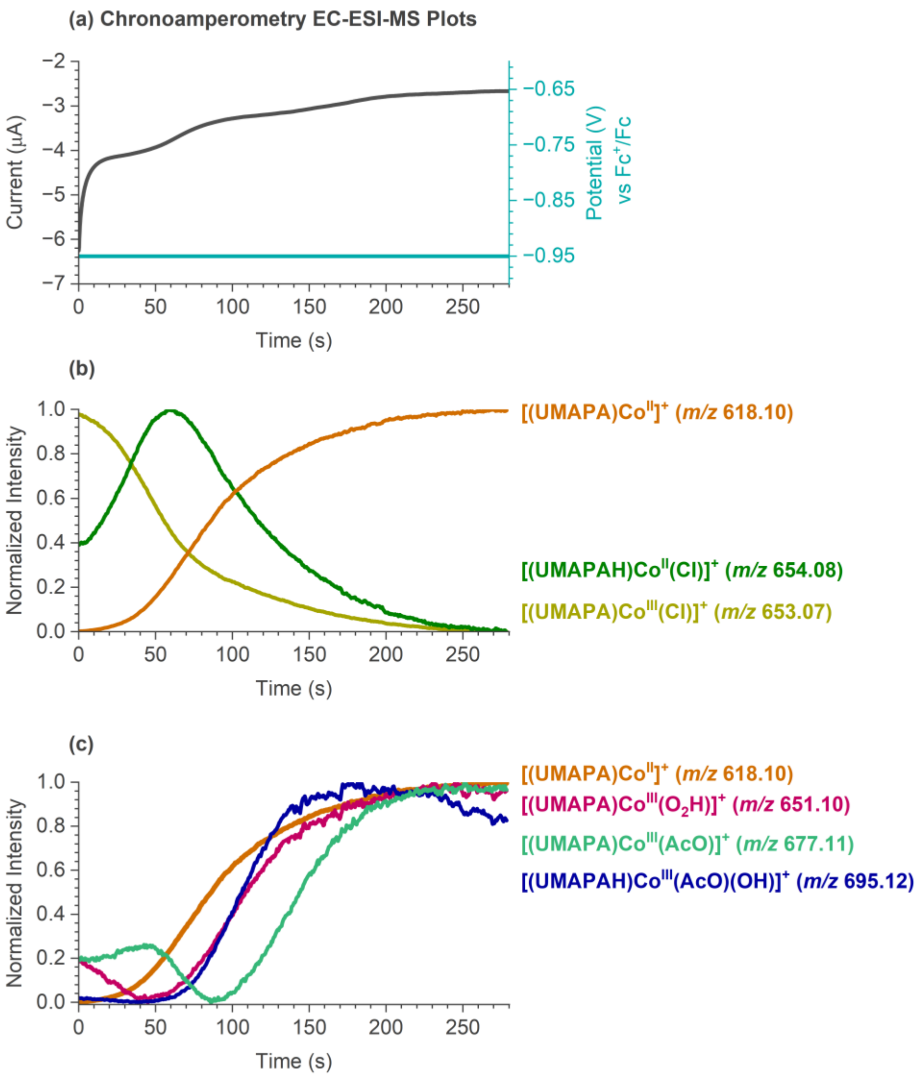

Figure S29. Chronoamperometry EC-ESI-MS experiments at -0.95 V vs Fc^+/Fc showing the evolution of ORR intermediates over time. (a) E vs t chronoamperometry plot. (b) and (c) Extracted ion chromatograms (EICs) vs time plots (the chromatograms are shifted by -20 s, which was the transfer time from the flowcell to ESI interface by the fused silica capillary length of 16.5 cm) and normalized from 0 to 1 for easier comparison. All the ions were generated from an O_2-saturated MeCN solution of 0.05 mM [(UMAPA)Co^{III}(Cl)]Cl complex with 5 mM AcOH (100 eq.) at a flow rate of 15 µL min-1. The reaction time in the flow cell was ~ 250 s.

2.6. Helium Tagging Infrared Photodissociation (IRPD) Spectroscopy Results

During a typical He-tagging IPRD experiment, [6, 7] the intermediates were generated using an EC-ESI-MS flow cell and transferred to the ESI source of our ISORI (Infrared Spectroscopy of Reaction Intermediates) instrument, which has quadrupole (Q1) – quadrupole bender (QPB) – octupole ion guide (O) – quadrupole ion trap (QIT) – quadrupole (Q2) geometry (Figure S30). The generated ions are mass-selected by Q1 and guided (QPB and O) toward the cryogenic quadrupole ion trap (QIT). The trap is cooled to temperatures ~3.4 K. The ions are trapped and thermalized in collisions with He, introduced in short pulses. The thermalized ions form He-tagged complexes. The ions are then irradiated by photons using the OPO/OPA system and extracted toward the second quadrupole, set to transmit only the ions with *m/z* of the helium complexes. The number of the surviving helium complexes is counted (Ni(ν)). In alternating cycles, the experiments are done without IR light irradiation, which gives the Ni0 number of the total number of He-tagged ions. The helium tagging IRPD spectra are then obtained as the relative attenuation (1 – Ni(ν)/Ni0) of the number of helium complexes as a function of the wavenumber (cm^{-1}). The He tagging IRPD spectrum of ions can be easily correlated with the DFT-predicted spectrum to provide detailed structural information. The DFT calculations were carried out first carried out at B97D/LANL2MB level to obtained the initial optimized geometry. Then with initially optimized geometry, the final DFT calculations (geometry optimization and frequency calculations) were carried at B3LYP-D3/def2svp (Opt Freq) level which provided the theoretical IR spectra.

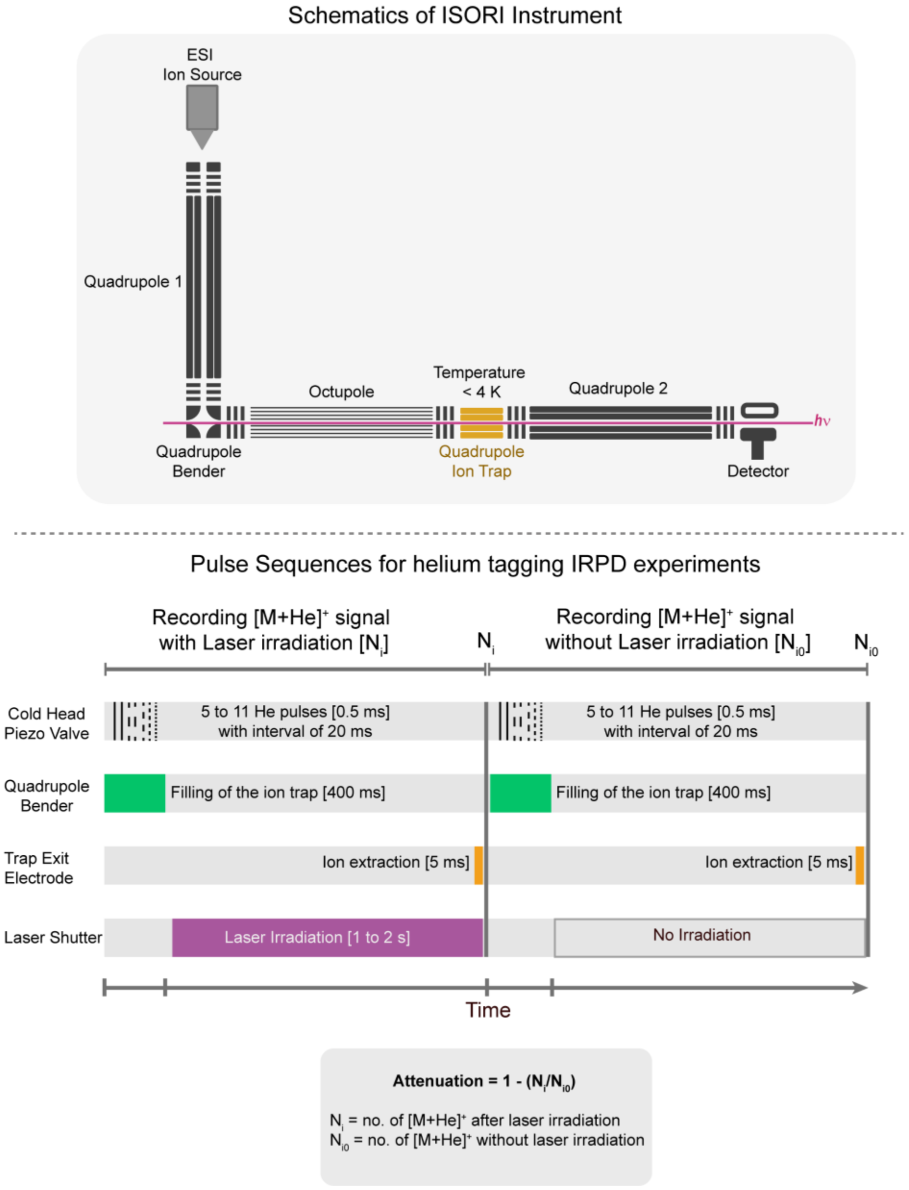

Figure S30: Sequences of events followed during a typical He tagging IRPD spectroscopy experiments.

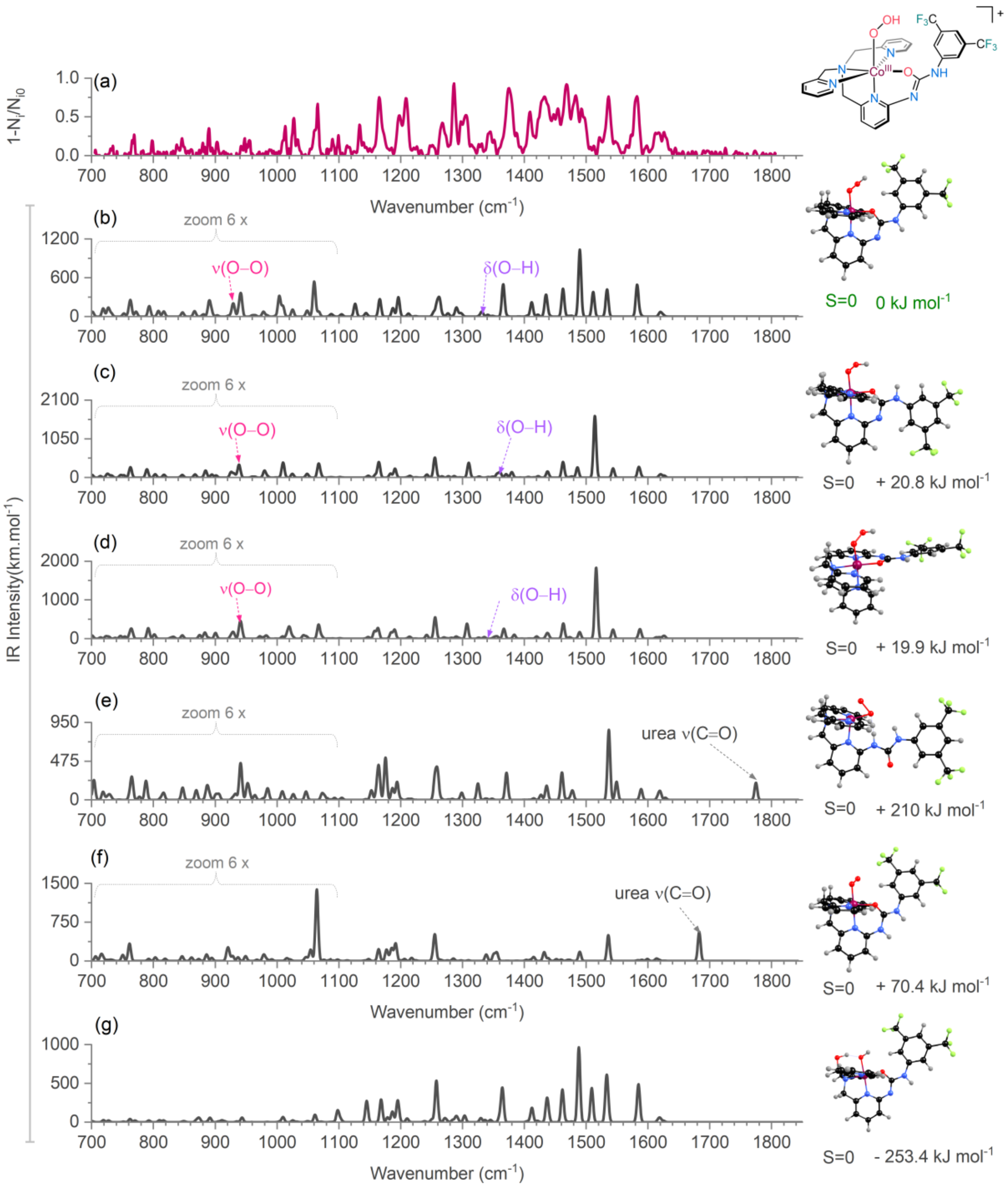

Figure S31. (a) The IRPD spectrum of ORR intermediate $[(UMAPA)Co^{III}(OOH)]^+$ (*m/z* 651.10). The ions were generated from the dry MeCN solution of 0.05 mM $[(UMAPA)Co^{III}(Cl)]Cl$ complex during the EC-ESI-MS experiment under O_2 saturation at -1.1 V Fc^+/Fc. (b to g) DFT predicted spectra of $[(UMAPA)Co^{III}(OOH)]^+$ complex in singlet spin state (S=0) with different geometries. The geometry (b) had the lowest energy. The calculations were carried out at B3LYP-D3/def2svp level. Scaling *0.955 for $v > 1800$ cm^{-1}, and *0.97 for $v < 1800$ cm^{-1}.

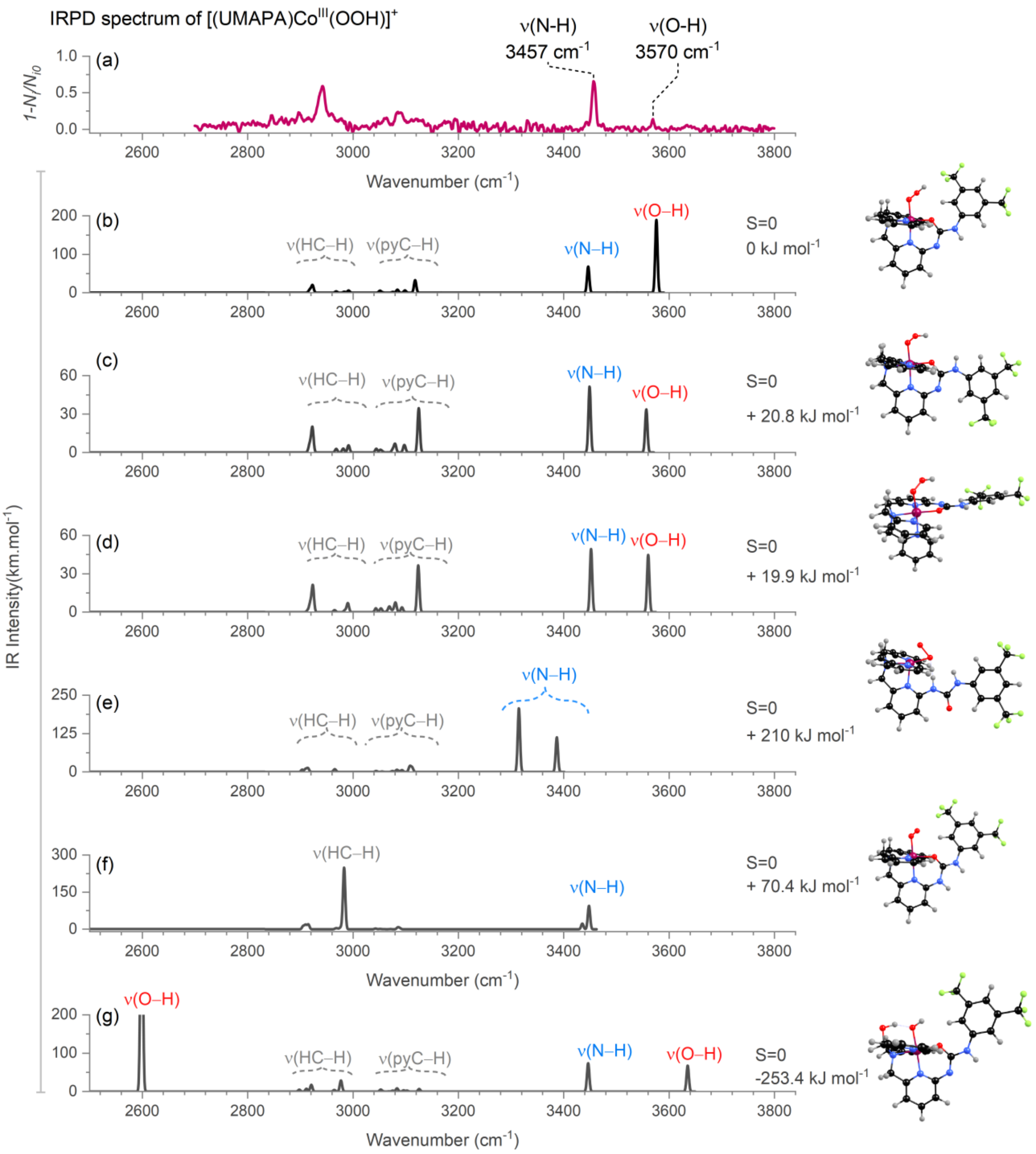

Figure S32. (a) The IRPD spectrum of ORR intermediate [(UMAPA)Co^{III}(OOH)]$^+$ (*m/z* 651.10) in range 2600 cm^{-1} to 3800 cm^{-1}. The ions were generated from the anhydrous MeCN solution of 0.05 mM [(UMAPA)Co^{III}(Cl)]Cl complex during the EC-ESI-MS experiment under O_2 saturation at -1.1 V Fc$^+$/Fc. (b to g) DFT predicted spectra of [(UMAPA)Co^{III}(OOH)]$^+$ complex in singlet spin state (S=0) with different geometries. The geometry (b) had the lowest energy. The calculations were carried out at B3LYP-D3/def2svp level. Scaling *0.955 for v > 1800 cm^{-1}, and *0.97 for v < 1800 cm^{-1}.

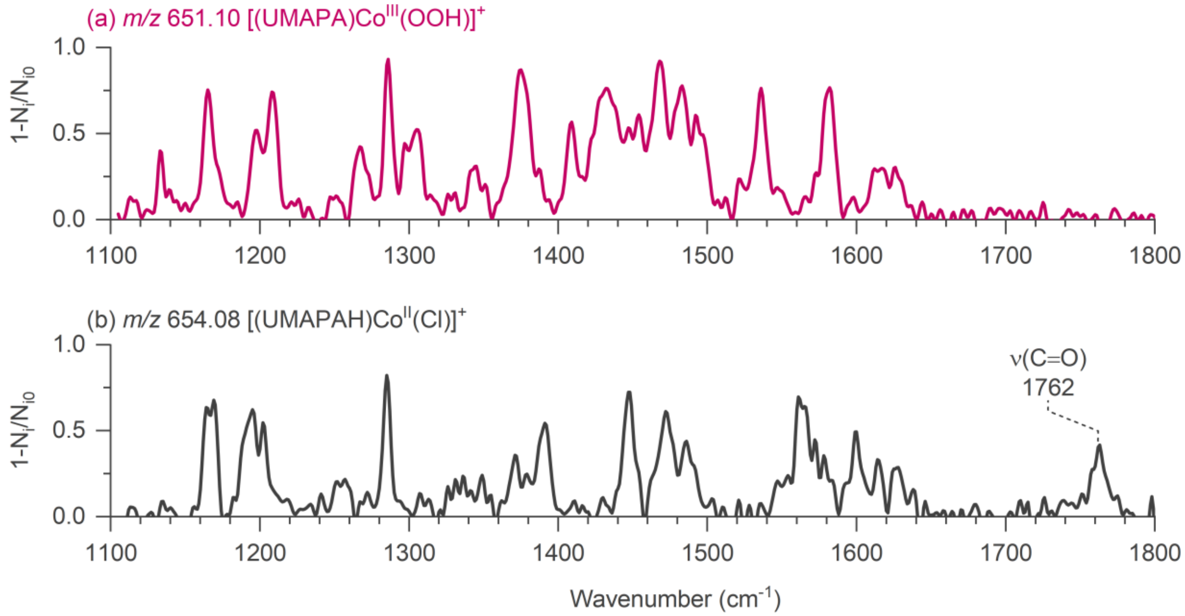

Figure S 33. (a) The IRPD spectrum of ORR intermediate $[(UMAPA)Co^{III}(OOH)]^+$ (*m/z* 651.10). The ions were generated from the dry MeCN solution of 0.05 mM cobalt complex during the EC-ESI-MS experiment under O_2 saturation at -1.1 V Fc^+/Fc. (b) The IRPD spectra of $[(UMAPAH)Co^{II}(Cl)]^+$ (*m/z* 654.08). The ions were generated from a MeCN solution of 0.05 mM cobalt complex without EC-ESI-MS experiments under N_2.

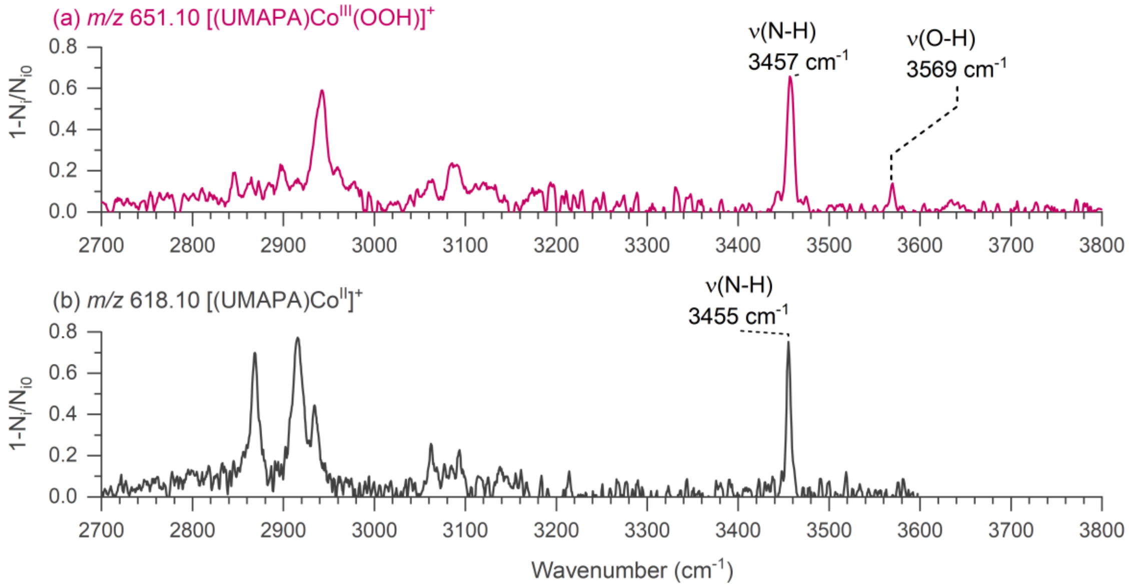

Figure S 34. Comparison of (a) the IRPD spectrum of ORR intermediate $[(UMAPA)Co^{III}(OOH)]^+$ (*m/z* 651.10). With (b) the IRPD spectrum of intermediate $[(UMAPA)Co^{II}]^+$ (*m/z* 618.10), in the C-H and N-H stretching vibration region. The ions were generated from the dry MeCN solution of 0.05 mM cobalt complex during the EC-ESI-MS experiment under O_2 saturation at -1.1 V Fc^+/Fc.

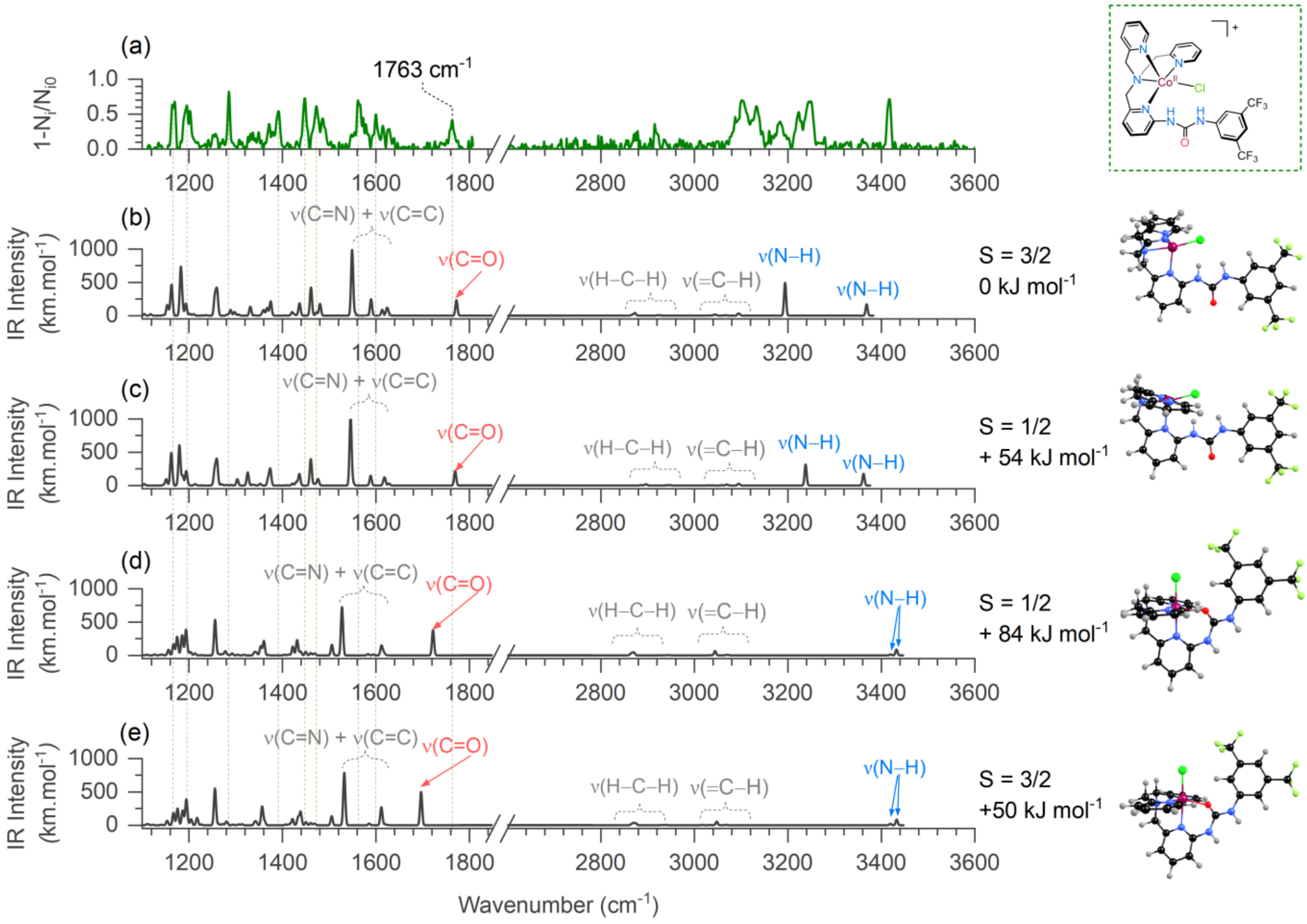

Figure S35. (a) The IRPD spectrum of Co(II)-chlorido complex [(UMAPAH)Co^{II}(Cl)]$^{+}$ (*m/z* 654.08). The spectrum was recorded from the MeCN solution of 0.05 mM [(UMAPAH)Co^{II}(Cl)]Cl complex. (b to f) DFT predicted spectra of [(UMAPAH)Co^{II}(Cl)]$^{+}$ (*m/z* 654.08) in different geometries and spin states. The geometry (b) with quartet spin state (S=3/2) had the lowest energy. The calculations were carried out at B3LYP-D3/def2svp level. Scaling *0.955 for *v* > 1800 cm^{-1}, and *0.97 for *v* < 1800 cm^{-1}.

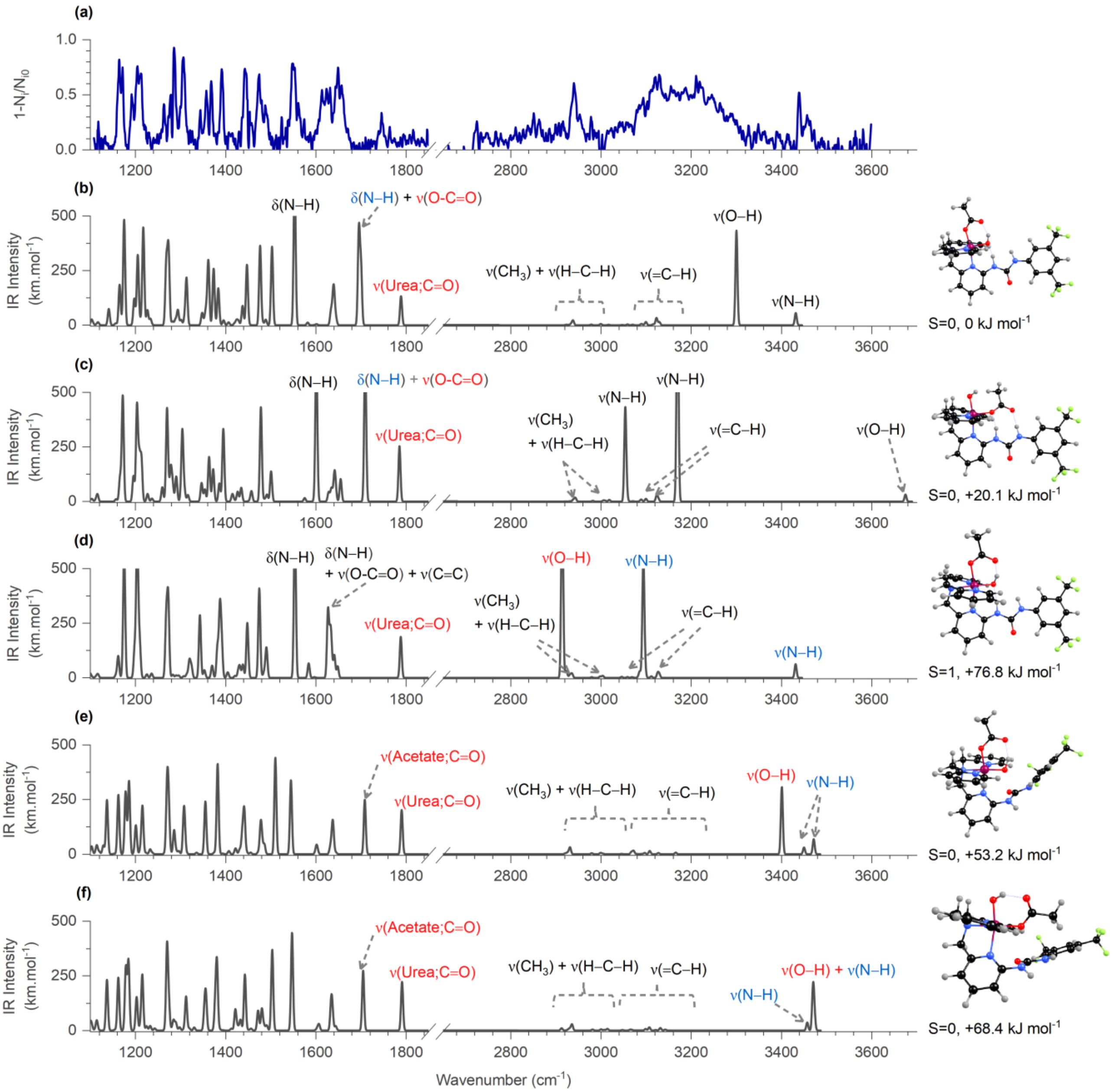

Figure S36. (a) The IRPD spectrum of $[(UMAPAH)Co^{III}(AcO)(OH)]^+$ (*m/z* 695.12). The ions were generated from the MeCN solution of 0.05 mM $[(UMAPA)Co^{III}(Cl)]Cl$ complex with H_2O_2 (15 mM) and 10 mM AcOH (100 eq.) during the EC-ESI-MS spectrum under O_2 at -0.95 V vs Fc^+/Fc. (b to c, and e-f) DFT predicted spectra of $[(UMAPAH)Co^{III}(AcO)(OH)]^+$ complex in singlet spin state (S=0) with different geometries. (d) DFT predicted spectra of $[(UMAPAH)Co^{III}(AcO)(OH)]^+$ in triplet spin state (S=1). The geometry (b) had the lowest energy. The calculations were carried out at B3LYP-D3/def2svp level. Scaling *0.955 for v > 1800 cm^{-1}, and *0.97 for v < 1800 cm^{-1}.

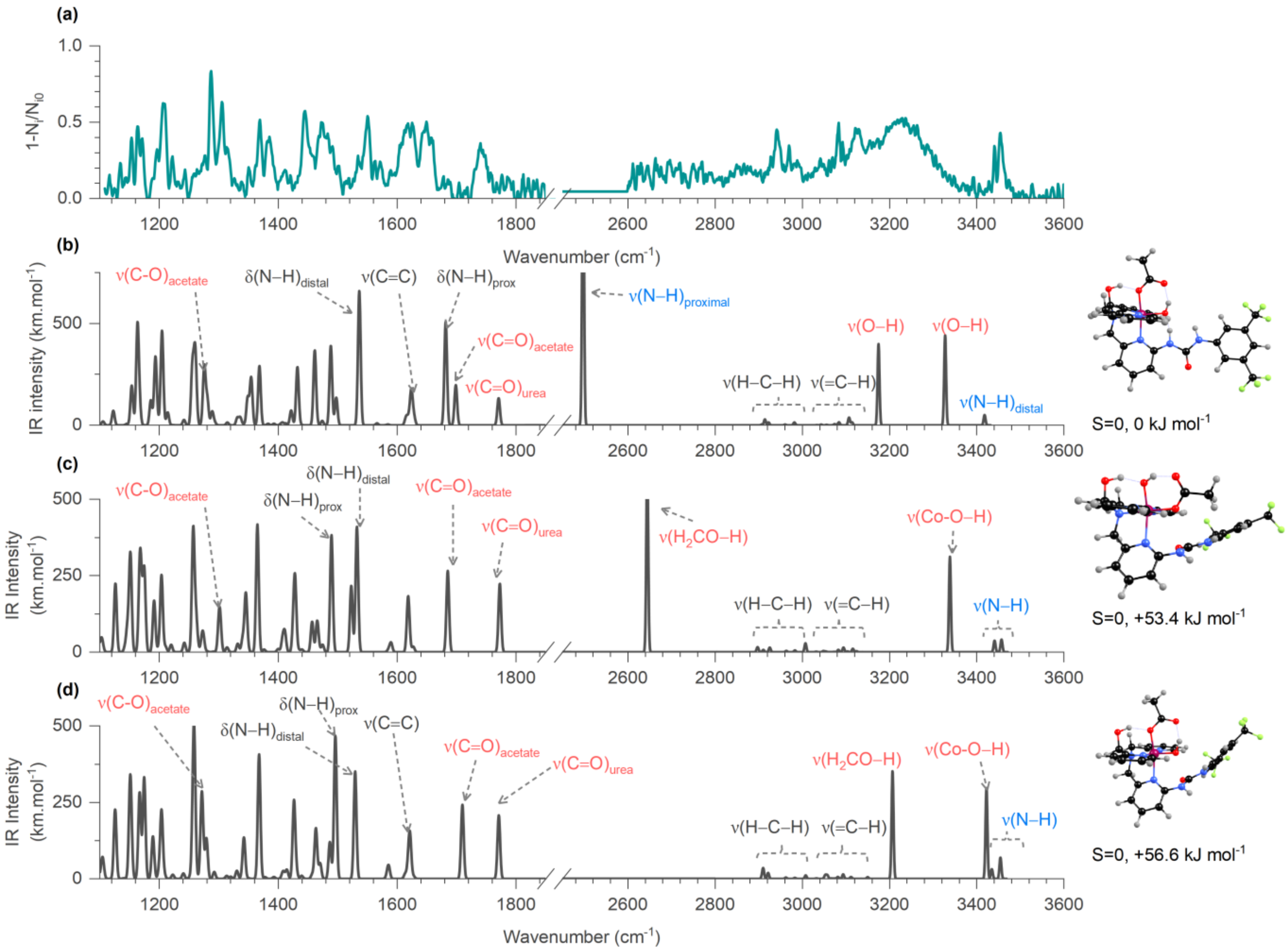

Figure S37. (a) The IRPD spectrum of $[(UMAPAH^{OH})Co^{III}(AcO)(OH)]^+$ (*m/z* 711.12). The ions were generated from the MeCN solution of 0.05 mM $[(UMAPA)Co^{III}(Cl)]Cl$ complex with H_2O_2 (15 mM) and 10 mM AcOH (100 eq.) during the EC-ESI-MS spectrum under O_2 at -0.95 V vs Fc^+/Fc. (b to d) DFT predicted spectra of $[(UMAPAH^{OH})Co^{III}(AcO)(OH)]^+$ complex in singlet spin state (S=0) with different geometries. The calculations were carried out at B3LYP-D3/def2svp level. Scaling *0.955 for v > 1800 cm^{-1}, and *0.97 for v < 1800 cm^{-1}.

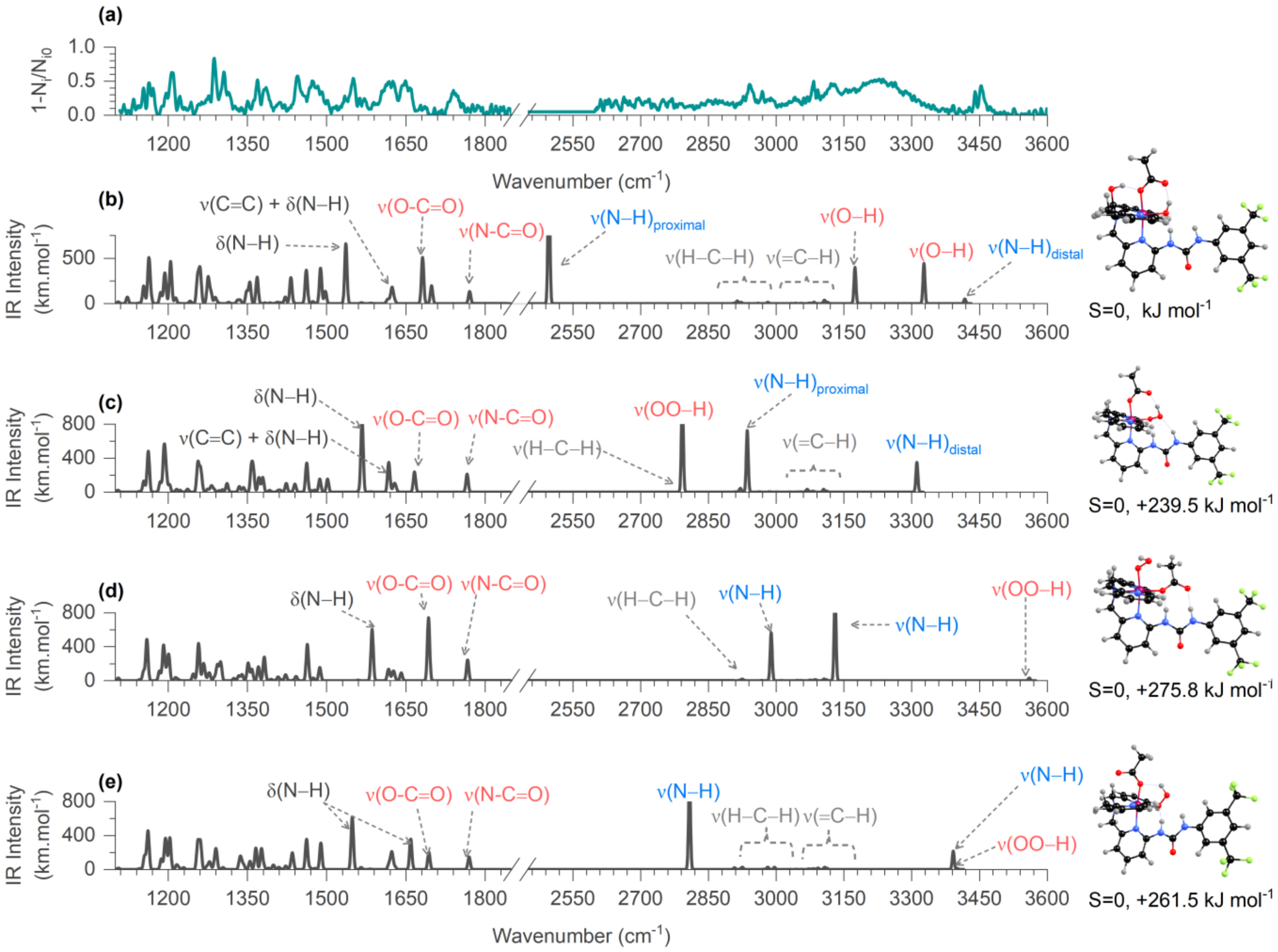

Figure S38. (a) The IRPD spectrum of $[(UMAPAH^{OH})Co^{III}(AcO)(OH)]^+$ (*m/z* 711.12). The ions were generated from the MeCN solution of 0.05 mM $[(UMAPA)Co^{III}(Cl)]Cl$ complex with H_2O_2 (15 mM) and 10 mM AcOH (100 eq.) during the EC-ESI-MS spectrum under O_2 at -0.95 V vs Fc^+/Fc. (b to d) DFT predicted spectra of $[(UMAPAH^{OH})Co^{III}(AcO)(OH)]^+$ complex in singlet spin state (S=0) with different geometries. The geometry (b) had the lowest energy. Structures in (c-e) represents the theoretical spectra of possible peroxo isomer $[(UMAPAH)Co^{III}(AcO)(OOH)]^+$. The calculations were carried out at B3LYP-D3/def2svp level. Scaling *0.955 for v > 1800 cm^{-1}, and *0.97 for v < 1800 cm^{-1}.

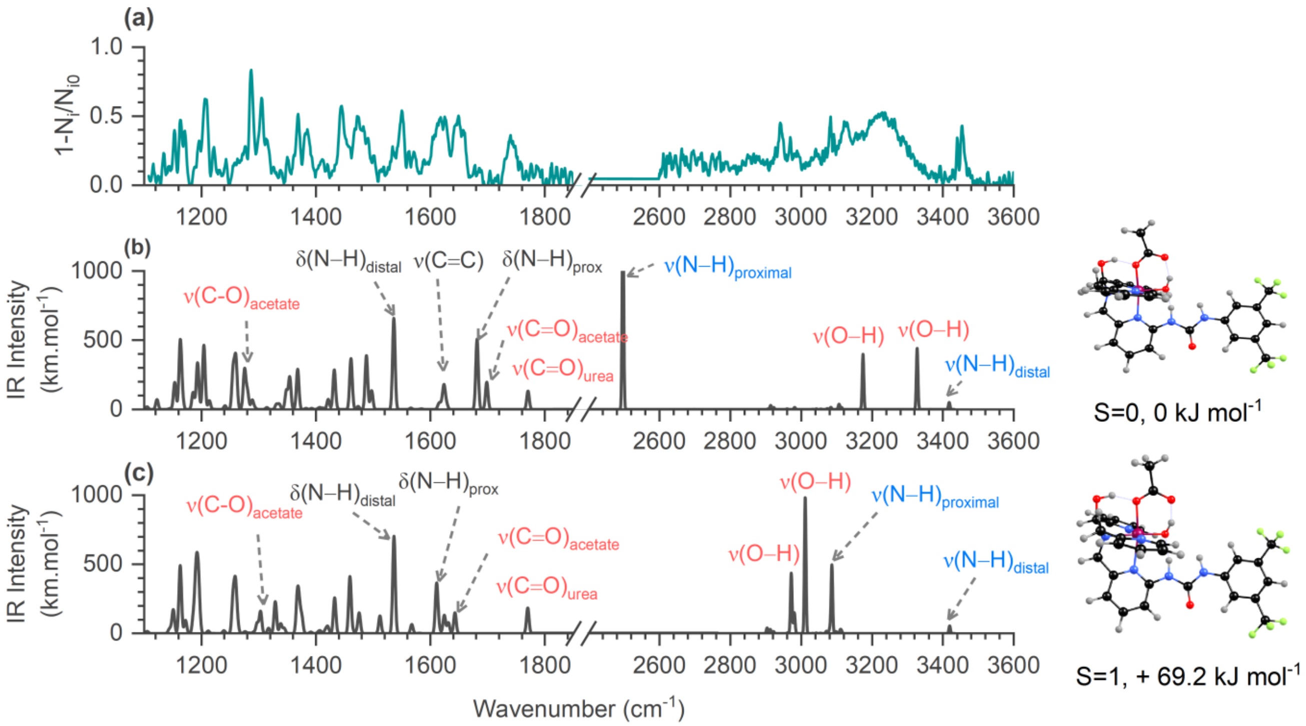

Figure S39. (a) The IRPD spectrum of $[(UMAPAH^{OH})Co^{III}(AcO)(OH)]^+$ (*m/z* 711.12). The ions were generated from the MeCN solution of 0.05 mM $[(UMAPA)Co^{III}(Cl)]Cl$ complex with H_2O_2 (15 mM) and 10 mM AcOH (100 eq.) during the EC-ESI-MS spectrum under O_2 at -0.95 V vs Fc^+/Fc. (b) DFT predicted spectra of $[(UMAPAH^{OH})Co^{III}(AcO)(OH)]^+$ complex in singlet spin state (S=0) with different geometries. (c) DFT predicted spectra of $[(UMAPAH^{OH})Co^{III}(AcO)(OH)]^+$ in triplet spin state (S=1). The calculations were carried out at B3LYP-D3/def2svp level. Scaling *0.955 for $v > 1800$ cm^{-1}, and *0.97 for $v < 1800$ cm^{-1}.

3. References

(1) Bakker, R.; Bairagi, A.; Rodriguez, M.; Tripodi, G. L.; Pereverzev, A. Y.; Roithova, J. Hydrogen Bonding Effect on the Oxygen Binding and Activation in Cobalt(III)-Peroxo Complexes. *Inorg. Chem.* **2023**, *62*, 1728-1734. DOI: 10.1021/acs.inorgchem.2c04260

(2) Zhou, R.; Zheng, Y.; Jaroniec, M.; Qiao, S.-Z. Determination of the Electron Transfer Number for the Oxygen Reduction Reaction: From Theory to Experiment. *Acs Catal* **2016**, *6*, 4720-4728. DOI: 10.1021/acscatal.6b01581

(3) Bairagi, A.; Pereverzev, A. Y.; Tinnemans, P.; Pidko, E. A.; Roithová, J. Electrocatalytic CO_2 Reduction: Monitoring of Catalytically Active, Downgraded, and Upgraded Cobalt Complexes. *J. Am. Chem. Soc.* **2024**, *146*, 5480-5492. DOI: 10.1021/jacs.3c13290

(4) Koovakattil Surendran, A.; Roithová, J. Decoding Voltammograms at the Molecular Frontier: Integration of Voltammetry and Mass Spectrometry. *Chemistry–Methods* **2024**, e202400003. DOI: https://doi.org/10.1002/cmtd.202400003

(5) Atobe, M.; Tateno, H.; Matsumura, Y. Applications of Flow Microreactors in Electrosynthetic Processes. *Chem Rev* **2018**, *118*, 4541-4572. DOI: 10.1021/acs.chemrev.7b00353

(6) Roithova, J.; Gray, A.; Andris, E.; Jasik, J.; Gerlich, D. Helium Tagging Infrared Photodissociation Spectroscopy of Reactive Ions. *Acc. Chem. Res.* **2016**, *49*, 223-230. DOI: 10.1021/acs.accounts.5b00489

(7) Jašík, J.; Žabka, J.; Roithová, J.; Gerlich, D. Infrared spectroscopy of trapped molecular dications below 4K. *Int. J. Mass spectrom.* **2013**, *354-355*, 204-210. DOI: https://doi.org/10.1016/j.ijms.2013.06.007
