# Supplementary material for: Primary and Secondary Coordination Sphere Effects in the Cobalt Complex-Catalyzed Electrocatalytic O2 Reduction to Water
Source: J Am Chem Soc. 2025 Oct 21;147(44):40440–53. doi: 10.1021/jacs.5c11990 (PMC12593408; doi:10.1021/jacs.5c11990)
Supplement: Supplementary file 3 [file ja5c11990_si_003.pdf]

[UMAPA-O_CoIII_AcO_OH]_Isomer2_1C1M.gjf.log

#N Geom=AllCheck Guess=TCheck SCRF=Check GenChk RB3LYP/def2SVP Freq
Charge = 1 Multiplicity = 1
Zero-point correction= 0.543211 (Hartree/Particle)
Thermal correction to Energy= 0.583553
Thermal correction to Enthalpy= 0.584497
Thermal correction to Gibbs Free Energy= 0.468294
Sum of electronic and zero-point Energies= -3804.839367
Sum of electronic and thermal Energies= -3804.799025
Sum of electronic and thermal Enthalpies= -3804.798081
Sum of electronic and thermal Free Energies= -3804.914284
Low frequencies --- -2.8813 -1.0960 -0.0049 -0.0040 0.0035 2.3090
Low frequencies --- 10.2810 14.1536 19.2081

[UMAPA-O_CoIII_AcO_OH]_Isomer3_1C1M.gjf.log

#N Geom=AllCheck Guess=TCheck SCRF=Check GenChk RB3LYP/def2SVP Freq
Charge = 1 Multiplicity = 1
Zero-point correction= 0.543572 (Hartree/Particle)
Thermal correction to Energy= 0.584237
Thermal correction to Enthalpy= 0.585181
Thermal correction to Gibbs Free Energy= 0.467265
Sum of electronic and zero-point Energies= -3804.838140
Sum of electronic and thermal Energies= -3804.797475
Sum of electronic and thermal Enthalpies= -3804.796531
Sum of electronic and thermal Free Energies= -3804.914447
Low frequencies --- -3.6394 0.0034 0.0041 0.0052 1.4695 2.0315
Low frequencies --- 6.2451 13.4573 15.8968

[UMAPA-O_CoIII_AcO_OH]_LowestE_1C1M.gjf.log

#N Geom=AllCheck Guess=TCheck SCRF=Check GenChk RB3LYP/def2SVP Freq
Charge = 1 Multiplicity = 1
Zero-point correction= 0.543165 (Hartree/Particle)
Thermal correction to Energy= 0.583753
Thermal correction to Enthalpy= 0.584697
Thermal correction to Gibbs Free Energy= 0.464201
Sum of electronic and zero-point Energies= -3804.859691
Sum of electronic and thermal Energies= -3804.819104
Sum of electronic and thermal Enthalpies= -3804.818160
Sum of electronic and thermal Free Energies= -3804.938656
Low frequencies --- -1.2900 -1.0717 -0.0021 0.0020 0.0035 1.9750
Low frequencies --- 7.3903 8.6852 10.6676

[UMAPA-O_CoIII_AcO_OH]_triplet_1C3M.gjf.log

#N Geom=AllCheck Guess=TCheck SCRF=Check GenChk UB3LYP/def2SVP Freq
Charge = 1 Multiplicity = 3
Zero-point correction= 0.542006 (Hartree/Particle)
Thermal correction to Energy= 0.583229
Thermal correction to Enthalpy= 0.584173
Thermal correction to Gibbs Free Energy= 0.460895

Sum of electronic and zero-point Energies= -3804.833318
Sum of electronic and thermal Energies= -3804.792096
Sum of electronic and thermal Enthalpies= -3804.791151
Sum of electronic and thermal Free Energies= -3804.914429
Low frequencies --- -0.6554 0.0033 0.0038 0.0064 1.4834 2.7289
Low frequencies --- 7.1957 9.0272 11.1483

[UMAPAH_CoIII_AcO_OH]_Isomer2_1C1M.gjf.log

#N Geom=AllCheck Guess=TCheck SCRF=Check GenChk RB3LYP/def2SVP Freq
Charge = 1 Multiplicity = 1
Zero-point correction= 0.538871 (Hartree/Particle)
Thermal correction to Energy= 0.578682
Thermal correction to Enthalpy= 0.579627
Thermal correction to Gibbs Free Energy= 0.461976
Sum of electronic and zero-point Energies= -3729.684419
Sum of electronic and thermal Energies= -3729.644608
Sum of electronic and thermal Enthalpies= -3729.643663
Sum of electronic and thermal Free Energies= -3729.761313
Low frequencies --- -2.0669 -1.3307 -0.0040 -0.0029 0.0020 2.2275
Low frequencies --- 8.3674 10.4769 12.2033

[UMAPAH_CoIII_AcO_OH]_Isomer3_1C1M.gjf.log

#N Geom=AllCheck Guess=TCheck SCRF=Check GenChk RB3LYP/def2SVP Freq
Charge = 1 Multiplicity = 1
Zero-point correction= 0.538448 (Hartree/Particle)
Thermal correction to Energy= 0.578407
Thermal correction to Enthalpy= 0.579352
Thermal correction to Gibbs Free Energy= 0.462219
Sum of electronic and zero-point Energies= -3729.671814
Sum of electronic and thermal Energies= -3729.631855
Sum of electronic and thermal Enthalpies= -3729.630911
Sum of electronic and thermal Free Energies= -3729.748043
Low frequencies --- -2.2574 -1.7536 -0.0116 -0.0045 -0.0030 0.5338
Low frequencies --- 5.6435 13.3261 15.0810

[UMAPAH_CoIII_AcO_OH]_Isomer4_1C1M.gjf.log

#N Geom=AllCheck Guess=TCheck SCRF=Check GenChk RB3LYP/def2SVP Freq
Charge = 1 Multiplicity = 1
Zero-point correction= 0.538692 (Hartree/Particle)
Thermal correction to Energy= 0.578471
Thermal correction to Enthalpy= 0.579415
Thermal correction to Gibbs Free Energy= 0.464436
Sum of electronic and zero-point Energies= -3729.666043
Sum of electronic and thermal Energies= -3729.626263
Sum of electronic and thermal Enthalpies= -3729.625319
Sum of electronic and thermal Free Energies= -3729.740298
Low frequencies --- -1.8696 -0.0031 0.0042 0.0062 0.9352 2.3612
Low frequencies --- 11.2153 14.0469 19.6003

[UMAPAH_CoIII_AcO_OH]_LowestE_1C1M.gjf.log

#N Geom=AllCheck Guess=TCheck SCRF=Check GenChk RB3LYP/def2SVP Freq
Charge = 1 Multiplicity = 1
Zero-point correction= 0.538089 (Hartree/Particle)
Thermal correction to Energy= 0.577974
Thermal correction to Enthalpy= 0.578918
Thermal correction to Gibbs Free Energy= 0.459266
Sum of electronic and zero-point Energies= -3729.692085
Sum of electronic and thermal Energies= -3729.652199
Sum of electronic and thermal Enthalpies= -3729.651255
Sum of electronic and thermal Free Energies= -3729.770907
Low frequencies --- -1.5017 -0.5889 -0.0049 -0.0037 0.0009 1.7064
Low frequencies --- 7.2282 8.5434 10.6375

[UMAPAH_CoIII_AcO_OH]_triplet_1C3M.gjf.log

#N Geom=AllCheck Guess=TCheck SCRF=Check GenChk UB3LYP/def2SVP Freq
Charge = 1 Multiplicity = 3
Zero-point correction= 0.537096 (Hartree/Particle)
Thermal correction to Energy= 0.577647
Thermal correction to Enthalpy= 0.578592
Thermal correction to Gibbs Free Energy= 0.456063
Sum of electronic and zero-point Energies= -3729.662845
Sum of electronic and thermal Energies= -3729.622293
Sum of electronic and thermal Enthalpies= -3729.621349
Sum of electronic and thermal Free Energies= -3729.743878
Low frequencies --- -1.9141 -1.2094 -0.0036 -0.0028 -0.0001 2.0060
Low frequencies --- 6.7923 8.5301 11.2110

[UMAPAH_CoIII_AcO_OOH]_Isomer1_1C1M.gjf.log

#N Geom=AllCheck Guess=TCheck SCRF=Check GenChk RB3LYP/def2SVP Freq
Charge = 1 Multiplicity = 1
Zero-point correction= 0.542472 (Hartree/Particle)
Thermal correction to Energy= 0.583301
Thermal correction to Enthalpy= 0.584245
Thermal correction to Gibbs Free Energy= 0.463069
Sum of electronic and zero-point Energies= -3804.768456
Sum of electronic and thermal Energies= -3804.727628
Sum of electronic and thermal Enthalpies= -3804.726684
Sum of electronic and thermal Free Energies= -3804.847860
Low frequencies --- -1.6613 -0.7842 -0.0033 -0.0027 0.0049 2.4060
Low frequencies --- 9.0103 10.2421 10.9995

[UMAPAH_CoIII_AcO_OOH]_Isomer2_1C1M.gjf.log

#N Geom=AllCheck Guess=TCheck SCRF=Check GenChk RB3LYP/def2SVP Freq
Charge = 1 Multiplicity = 1
Zero-point correction= 0.542120 (Hartree/Particle)
Thermal correction to Energy= 0.583267
Thermal correction to Enthalpy= 0.584211
Thermal correction to Gibbs Free Energy= 0.463032

Sum of electronic and zero-point Energies= -3804.760104
Sum of electronic and thermal Energies= -3804.718957
Sum of electronic and thermal Enthalpies= -3804.718013
Sum of electronic and thermal Free Energies= -3804.839192
Low frequencies --- -2.1875 -0.7875 0.0024 0.0034 0.0042 1.7737
Low frequencies --- 7.6345 9.6202 11.5761

[UMAPAH_CoIII_AcO_OOH]_Isomer3_1C1M.gjf.log

#N Geom=AllCheck Guess=TCheck SCRF=Check GenChk RB3LYP/def2SVP Freq
Charge = 1 Multiplicity = 1
Zero-point correction= 0.542359 (Hartree/Particle)
Thermal correction to Energy= 0.583539
Thermal correction to Enthalpy= 0.584483
Thermal correction to Gibbs Free Energy= 0.463880
Sum of electronic and zero-point Energies= -3804.754657
Sum of electronic and thermal Energies= -3804.713477
Sum of electronic and thermal Enthalpies= -3804.712533
Sum of electronic and thermal Free Energies= -3804.833136
Low frequencies --- -0.0088 0.0016 0.0024 0.2388 2.0001 3.3358
Low frequencies --- 7.9171 10.4270 12.0223

[UMAPAH_CoII_Cl]_Dublet_1C2M.gjf.log

#N Geom=AllCheck Guess=TCheck SCRF=Check GenChk UB3LYP/def2SVP Freq
Charge = 1 Multiplicity = 2
Zero-point correction= 0.472263 (Hartree/Particle)
Thermal correction to Energy= 0.508097
Thermal correction to Enthalpy= 0.509041
Thermal correction to Gibbs Free Energy= 0.397620
Sum of electronic and zero-point Energies= -3885.745531
Sum of electronic and thermal Energies= -3885.709698
Sum of electronic and thermal Enthalpies= -3885.708753
Sum of electronic and thermal Free Energies= -3885.820174
Low frequencies --- -2.9135 -0.0067 -0.0042 -0.0040 0.6762 2.2295
Low frequencies --- 6.9947 9.0674 11.4211

[UMAPAH_CoII_Cl]_Isomer2_1C2M.gjf.log

#N Geom=AllCheck Guess=TCheck SCRF=Check GenChk UB3LYP/def2SVP Freq
Charge = 1 Multiplicity = 2
Zero-point correction= 0.470835 (Hartree/Particle)
Thermal correction to Energy= 0.506976
Thermal correction to Enthalpy= 0.507920
Thermal correction to Gibbs Free Energy= 0.397117
Sum of electronic and zero-point Energies= -3885.734191
Sum of electronic and thermal Energies= -3885.698050
Sum of electronic and thermal Enthalpies= -3885.697106
Sum of electronic and thermal Free Energies= -3885.807909
Low frequencies --- -0.0058 -0.0035 0.0020 1.2757 2.6452 3.8224
Low frequencies --- 9.6202 11.6822 12.0122

[UMAPAH_CoII_Cl]_Isomer3_1C4M.gjf.log

#N Geom=AllCheck Guess=TCheck SCRF=Check GenChk UB3LYP/def2SVP Freq
Charge = 1 Multiplicity = 4
Zero-point correction= 0.469782 (Hartree/Particle)
Thermal correction to Energy= 0.506501
Thermal correction to Enthalpy= 0.507446
Thermal correction to Gibbs Free Energy= 0.394332
Sum of electronic and zero-point Energies= -3885.747038
Sum of electronic and thermal Energies= -3885.710319
Sum of electronic and thermal Enthalpies= -3885.709375
Sum of electronic and thermal Free Energies= -3885.822488
Low frequencies --- -1.2419 -0.0069 -0.0038 -0.0021 0.8465 1.9927
Low frequencies --- 11.0502 11.4616 13.5038

[UMAPAH_CoII_Cl]_LowestE_1C4M.gjf.log

#N Geom=AllCheck Guess=TCheck SCRF=Check GenChk UB3LYP/def2SVP Freq
Charge = 1 Multiplicity = 4
Zero-point correction= 0.471061 (Hartree/Particle)
Thermal correction to Energy= 0.507507
Thermal correction to Enthalpy= 0.508452
Thermal correction to Gibbs Free Energy= 0.394755
Sum of electronic and zero-point Energies= -3885.766104
Sum of electronic and thermal Energies= -3885.729658
Sum of electronic and thermal Enthalpies= -3885.728713
Sum of electronic and thermal Free Energies= -3885.842410
Low frequencies --- -1.2048 -0.5828 -0.0053 -0.0045 -0.0038 1.4156
Low frequencies --- 8.0963 9.6267 11.8566

[UMAPA_CoIII_OOH]_Isomer2_1C1M.gjf.log

#N Geom=AllCheck Guess=TCheck SCRF=Check GenChk RB3LYP/def2SVP Freq
Charge = 1 Multiplicity = 1
Zero-point correction= 0.477588 (Hartree/Particle)
Thermal correction to Energy= 0.513463
Thermal correction to Enthalpy= 0.514407
Thermal correction to Gibbs Free Energy= 0.405178
Sum of electronic and zero-point Energies= -3575.865826
Sum of electronic and thermal Energies= -3575.829952
Sum of electronic and thermal Enthalpies= -3575.829008
Sum of electronic and thermal Free Energies= -3575.938236
Low frequencies --- -0.0083 -0.0020 0.0033 0.4059 1.5389 2.2354
Low frequencies --- 7.5692 9.8988 12.0626

[UMAPA_CoIII_OOH]_Isomer3_1C1M.gjf.log

#N Geom=AllCheck Guess=TCheck SCRF=Check GenChk RB3LYP/def2SVP Freq
Charge = 1 Multiplicity = 1
Zero-point correction= 0.477769 (Hartree/Particle)
Thermal correction to Energy= 0.513513
Thermal correction to Enthalpy= 0.514457
Thermal correction to Gibbs Free Energy= 0.406028

Sum of electronic and zero-point Energies= -3575.866191
Sum of electronic and thermal Energies= -3575.830447
Sum of electronic and thermal Enthalpies= -3575.829503
Sum of electronic and thermal Free Energies= -3575.937932
Low frequencies --- -0.0047 -0.0035 -0.0026 1.4212 2.3438 3.6239
Low frequencies --- 8.3736 11.8599 14.7351

[UMAPA_CoIII_OOH]_Isomer4_1C1M.gjf.log

#N Geom=AllCheck Guess=TCheck SCRF=Check GenChk RB3LYP/def2SVP Freq
Charge = 1 Multiplicity = 1
Zero-point correction= 0.478413 (Hartree/Particle)
Thermal correction to Energy= 0.513477
Thermal correction to Enthalpy= 0.514421
Thermal correction to Gibbs Free Energy= 0.407424
Sum of electronic and zero-point Energies= -3575.970279
Sum of electronic and thermal Energies= -3575.935216
Sum of electronic and thermal Enthalpies= -3575.934271
Sum of electronic and thermal Free Energies= -3576.041269
Low frequencies --- -2.4832 -0.5924 -0.0007 0.0017 0.0103 2.6744
Low frequencies --- 6.3672 11.5740 12.9699

[UMAPA_CoIII_OOH]_LowestE_1C1M.gjf.log

#N Geom=AllCheck Guess=TCheck SCRF=Check GenChk RB3LYP/def2SVP Freq
Charge = 1 Multiplicity = 1
Zero-point correction= 0.478015 (Hartree/Particle)
Thermal correction to Energy= 0.513608
Thermal correction to Enthalpy= 0.514552
Thermal correction to Gibbs Free Energy= 0.407761
Sum of electronic and zero-point Energies= -3575.873755
Sum of electronic and thermal Energies= -3575.838162
Sum of electronic and thermal Enthalpies= -3575.837218
Sum of electronic and thermal Free Energies= -3575.944009
Low frequencies --- -3.4799 -2.3204 -0.0047 -0.0016 0.0032 1.4971
Low frequencies --- 8.8370 12.7605 15.0402

[UMAPA_CoIII_OOH]_triplet_1C3M.gjf.log

#N Geom=AllCheck Guess=TCheck SCRF=Check GenChk UB3LYP/def2SVP Freq
Charge = 1 Multiplicity = 3
Zero-point correction= 0.475515 (Hartree/Particle)
Thermal correction to Energy= 0.512075
Thermal correction to Enthalpy= 0.513019
Thermal correction to Gibbs Free Energy= 0.402405
Sum of electronic and zero-point Energies= -3575.853951
Sum of electronic and thermal Energies= -3575.817390
Sum of electronic and thermal Enthalpies= -3575.816446
Sum of electronic and thermal Free Energies= -3575.927060
Low frequencies --- -2.2807 -0.0076 -0.0030 -0.0023 1.1433 1.9931
Low frequencies --- 9.5664 12.5706 15.5912
